# Effects of ultra-processed foods on the liver: insights from gut microbiome and metabolomics studies in rats

**DOI:** 10.3389/fnut.2024.1503879

**Published:** 2025-01-22

**Authors:** Liping Shi, Zhuoyuan Li, Xiaojun Ma, Junru Wang, Yueping Wu, Yongbin Zhu, Yanrong Wang, Yue Yang, Minxiu Luo, Jiangping Li, Xian Sun, Shulan He

**Affiliations:** ^1^Department of Epidemiology and Health Statistics, School of Public Health, Ningxia Medical University, Yinchuan, China; ^2^Department of Biochemistry and Molecular Biology, School of Basic Medical Sciences, Ningxia Medical University, Yinchuan, China; ^3^Key Laboratory of Environmental Factors and Chronic Disease Control, Ningxia Medical University, Yinchuan, China

**Keywords:** ultra-processed foods, gut microbiome, metabolomics, liver, rat

## Abstract

**Purpose:**

High consumption of Ultra-processed foods (UPF) have been identified as a potential risk factor for Non-alcoholic fatty liver disease (NAFLD). Nevertheless, there is limited empirical evidence regarding the impact of UPF, which are typical combination of processed foods, on liver health through alterations in gut microbiota and metabolic processes. We aim to examine the potential impact of UPF on liver health and to explore the role of gut microbiota and metabolites.

**Methods:**

This study used Sprague–Dawley rats to mimic modern UPF diets for 90 days. Some serum biochemical indices, inflammatory factors, oxidative stress markers, hematoxylin–eosin (HE) staining of the liver, 16S ribosomal RNA (rRNA) and Liquid chromatography-mass spectrometry (LC–MS) of rat feces were detected.

**Results:**

The UPF diet-induced simple steatosis of the liver in rats without affecting the levels of IL-6, GSH, MDA, and SOD. Additionally, it modified the gut microbiota, increasing potentially harmful bacteria, such as *norank_f__Desulfovibrionaceae* and *Staphylococcus*, while also elevating the relative abundance of potentially beneficial bacteria, including *Dubosiella* and *Allobaculum*. Furthermore, the consumption of UPF led to a metabolomic disorder characterized by disruptions in the sphingolipid signaling pathway, sulfur relay system, and arachidonic acid metabolism.

**Conclusion:**

In conclusion, the findings of this study indicate that the consumption of UPF influences the development of simple hepatic steatosis, potentially through alterations in gut microbiota and metabolomics.

## 1 Introduction

The global intake of UPF worldwide has risen significantly (1). According to the Nova classification system, UPF encompasses industrially manufactured products such as beverages, baked goods, snacks, and ready-to-eat meals. These items are composed of food-derived ingredients, and additives, characterized by high energy density, elevated levels of salt, sugars, unhealthy fats, and refined carbohydrates (2). These attributes contribute to the palatability, attractiveness, and potential addictiveness of UPF (3). A cross-sectional study conducted at multiple time points revealed an increase in the proportion of total energy intake derived from UPF among U.S. adolescents between the ages of 2 and 19, rising from 61.4 to 67.0%. Conversely, the percentage of energy obtained from unprocessed or minimally processed foods decreased from 28.8 to 23.5% (1). Furthermore, UPF sales are rapidly increasing in Asia (4). Specifically, in China, the per capita sales of dried and prepared baby foods for children under 5 years of age increased between 2014 and 2019 (5). According to An et al., there was a notable upward trend in the consumption of UPF among children across different age groups (6). Specifically, the proportion of UPF intake was 73.8% among children aged 6 to 24 months, which increased to 98.2% for those between aged 25 to 36 months. In China, pastries represented the largest share of children’s UPF consumption at 63.5%, followed by dairy products in solid or semi-solid form at 58.8%, and reconstituted meat items at 56.4% (6).

Epidemiological studies have demonstrated an association between the intake of UPF and numerous adverse health outcomes, such as obesity, hypertension, diabetes, and increased mortality (7). An expanding body of evidence indicates that UPF consumption exerts a significant impact on liver health. NAFLD includes a spectrum of hepatic alterations that may progress to severe illness and potentially result in mortality, has an estimated global prevalence of 32.4% among the adult population (8). Moreover, NAFLD impacts various metabolic pathways and is intricately linked to metabolic syndrome (MetS), insulin resistance (IR), type 2 diabetes (T2D), and obesity. A systematic review of 15 studies, encompassing 52,885 participants, indicated that UPF may not only serve as a risk factor for NAFLD but is also associated with its primary risk factors, including obesity, T2D, and MetS (9). Additionally, a recent investigation from the PREDIMED-Plus cohort demonstrated that increased UPF consumption correlates with heightened visceral and total fat accumulation, as well as elevated NAFLD-related biomarkers in older adults with overweight or obesity (10, 11). The Tianjin Chronic Low-Grade Systemic Inflammation and Health Cohort Study (TCLSIH), indicates a positive correlation between the consumption of UPF and the prevalence of NAFLD (12). However, the specific mechanisms by which UPF affects liver health are not yet fully understood. Although research has independently examined the effects of excessive intake of fats and free sugars, as well as inadequate fiber consumption, on liver health, there is a paucity of studies that explicitly characterize the impact of UPF exposure, particularly in terms of common food combinations.

The gut microbiota is integral to the regulation of host health, influencing numerous functions of the gastrointestinal tract, including dietary digestion, nutrient absorption, immunity, hormone synthesis, and neural conduction (13, 14). A compromised gut barrier and disrupted gut-liver axis are common features of many liver diseases (15). Diet is recognized as a critical factor in modulating the composition of the gut microbiota and, consequently, affecting microbial metabolites (16). Food additives, such as emulsifiers, sweeteners, colorants, microparticles, and nanoparticles, commonly found in UPF, can impact the gut microbiome (17). A conducted study on mice fed UPF from a well-known fast food chain, in comparison to those fed standard chow, demonstrated a reduction in beta diversity and an increased abundance of *Bifidobacterium* and *Parasutterella* in the UPF group (18). Additionally, rodents subjected to a thermally processed diet have been reported to exhibit heightened intestinal permeability and elevated production of advanced glycation end products, which are commonly associated with UPF (19). These findings suggest that the effects of UPF on gut microbiome and metabolism may be a causal mechanism underlying the increased risk of liver health issues.

In this study, Sprague–Dawley rats were used as a model to simulate contemporary Chinese dietary patterns. The effects of the UPF diet were assessed through biochemical analysis, inflammatory markers, oxidative stress, HE staining of the liver, intestinal microbiome analysis, and metabolomics techniques, to explore the relationships between UPF, the gut microbiome, metabolomics, and liver health.

## 2 Methods and materials

### 2.1 Experiment design

Forty Sprague–Dawley rats (specific pathogen-free grad, 3 weeks old) were procured from the Experimental Animal Center at Ningxia Medical University located in Ningxia, China. The study protocol received approval from the Ethics Committee of Ningxia Medical University (Approval Number: 2022-N020). The rats were kept with standard conditions (temperature 22 ± 1°C, relative humidity 50 ± 20%, and 12-h light/dark cycle). Following a one-week acclimation period, the rats were randomly assigned to four groups: the beverage group, the high-frequency UPF group, the low-frequency UPF group, and the control group. Each group comprised five male and five female rats, housed two or three per cage. Both groups were fed for 90 days, with the specific experimental process demonstrated in Supplementary Figure S1. Throughout the feeding period, the rats had ad libitum access to food and water. Body weight and feed consumption were recorded every 10 days while fasting blood glucose levels were measured every 30 days.

### 2.2 UPF diet

Based on a market report on the consumption of UPF in China, several UPF with high consumption rates among children were selected to replicate contemporary Chinese dietary patterns (5). Packaged ready-to-eat potato chips, waffles, pork jerky, and melon seeds were procured from a snack shop, homogenized, formed into rod-shaped pieces, sterilized using cobalt 60 lamp irradiation, and stored at 4°C. The diet had an energy density of 4.84 kcal/g, with 12.1% of calories from protein, 41.9% from complex carbohydrates, and 46.0% from fat (20). The experiment included four distinct diet groups, which were provided as follows:

(1) Beverage group (UPD) (*n* = 10): A mixture of 22 g of milk tea powder and 120 mL hot water was prepared following the manufacturer’s instructions for milk tea preparation. Following autoclave sterilization, it was sealed and stored at 4°C. The rats consumed the beverage for one-sixth of the 6 days, while distilled water was provided for five-sixths of the time (i.e., one-sixth beverage / five-sixths distilled water), with a standard diet provided throughout.
(2) High-frequency UPF group (HUPF) (*n* = 10): The rats were fed a standard diet for 70% of the 10 days, with UPF constituting the remaining 30% of their intake (i.e., 70% standard diet / 30% UPF), with distilled water provided throughout.
(3) Low-frequency UPF group (LUPF) (*n* = 10): The rats were fed a standard diet for 90% of the 10 days, with UPF constituting the remaining 10% of their intake (i.e., 90% standard diet / 10% UPF), with distilled water provided throughout.
(4) The control group (*n* = 10): The rats received a standard diet, purchased from Jiangsu Xietong Pharmaceutical Bio-engineering Co., Ltd. This diet had an energy density of 3.8 kcal/g, comprising 22.9% of calories from protein, 66.0% from complex carbohydrates, and 11.1% from fat, with distilled water provided throughout.

### 2.3 Sample collection

After a 90-day exposure, fecal samples were collected from individual fasted rats housed in metabolic cages with access to water on the final day of the feeding regimen. These samples were stored in sterile cryopreservation tubes. Following anesthesia and dissection, serum was isolated by centrifuging blood obtained from the abdominal aorta at 3000 xg for 15 min at 4°C, and the serum was stored at −80°C.

### 2.4 Histological analysis

Livers from rats in various experimental groups were excised and fixed with 4% paraformaldehyde for 24 h. The fresh tissues were then embedded in paraffin, dehydrated using graded ethanol, and sectioned into 5 μm slices for H-E staining to be employed to evaluate tissue structure.

### 2.5 Biochemical analysis of serum, assay of inflammatory factors, and oxidative stress

The quantification of alanine aminotransferase (ALT), aspartate aminotransferase (AST), cholesterol (CHOL), triglycerides (TG), alkaline phosphatase (ALP), total protein (TP), albumin (ALB), Globulin (GLO), and glucose (GLU) in thawed serum were conducted with an automatic biochemical analyzer (UC400, Olympus Corporation, Japan). In addition, the levels of the inflammatory marker interleukin-6 (IL-6), along with glutathione (GSH), superoxide dismutase (SOD), and malondialdehyde (MDA), were determined using an enzyme-linked immunosorbent assay (ELISA) kit.

### 2.6 16S rRNA sequencing analysis of feces

Genomic DNA from microbial sources was isolated from fecal specimens utilizing the PF Mag-Bind Stool DNA Kit (Omega Bio-tek, United States), following the manufacturer’s guidelines. The quality of the extracted DNA was evaluated through 1% agarose gel electrophoresis, while its concentration and purity were quantified using a NanoDrop2000 (Thermo Scientific, USA). Amplification of the V3-V4 region of the 16S rRNA gene was conducted via PCR using the primers 338F and 806R, with each reaction conducted in triplicate. The sequencing of the purified DNA libraries was performed using an Illumina MiSeq PE300 platform (Majorbio Bio-Pharm Technology, China). Amplicon Sequence Variants (ASVs) were categorized utilizing QIIME2 (version 2020.2) against the Silva138/6S_bacteria Escherichia database. Further details on the PCR conditions and data analysis are provided in the Supplementary Table S1.

### 2.7 LC–MS untargeted metabolomics analysis of feces

The metabolites were extracted by combining a 50 mg fecal sample with a 6 mm grinding bead in a 2 mL centrifuge tube and adding 400 μL of an extraction solution (methanol: water = 4:1 (v/v)) containing 0.02 mg/mL of L-2-chlorophenyl alanine serving as the internal standard. The mixture was ground for 6 min at −10°C and 50 Hz, then subjected to cryo-sonication at 5°C and 40 kHz for 30 min. Subsequently, the samples were incubated at −20°C for 30 min and centrifuged at 13,000 g for 15 min at 4°C. The supernatant was collected and transferred into injection vials for further analysis. A quality control (QC) sample was generated by combining equal volumes from all samples, with QC analysis conducted for every 5–15 samples. LC–MS analysis was executed using a UPLC-Triple TOF system (Thermo Fisher Scientific, United States). Further details regarding instrument conditions and data analysis can be found in the Supplementary material.

### 2.8 Statistical analysis

The analysis was performed using SPSS 26.0 and presented as mean + standard error of the mean (S.E.M). For data exhibiting normal distribution and homogeneity of variances, one-way ANOVA was conducted, followed by Tukey’s *post hoc* test. In cases of non-parametric data, Kruskal-Wallis and Dunn’s test were used. Visualization was performed with R 4.4.0. A *p*-value of less than 0.05 was deemed statistically significant.

## 3 Result

### 3.1 Impact of UPF on growth performance, liver histology

**Figure 1:**
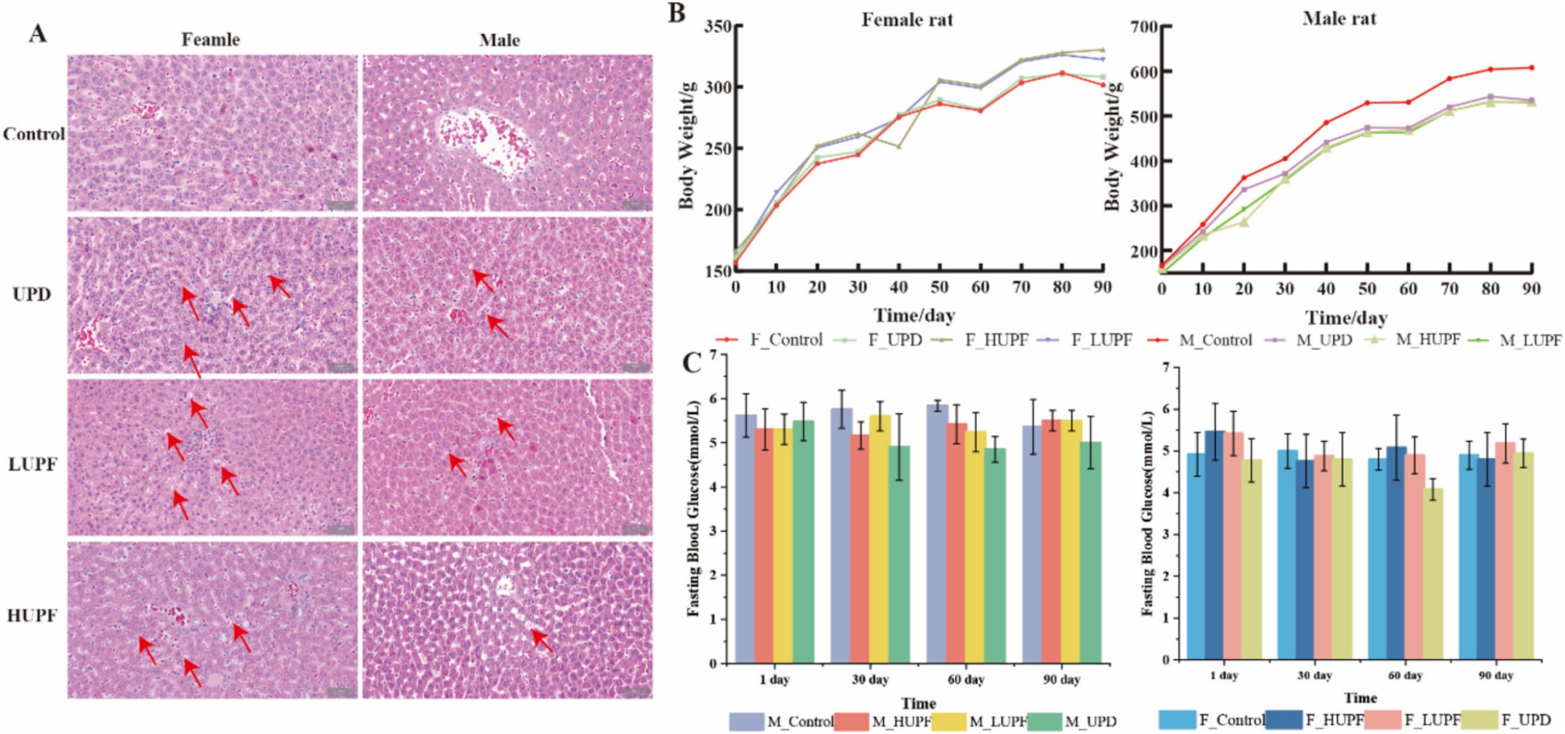
H&E staining image of the female and male rats’ liver in each group (scale bar, 50 μm) **(A)**, Body weight of rats **(B)** and Fasting blood glucose **(C)**. Red arrows show lipid droplets. F_Control: Control group in female rats; F_UPD: Beverage group in female rats; F_HUPF: High-frequency UPF group in female rats; F_LUPF: Low-frequency UPF group in female rats; M_Control: Control group in male rats; M_UPD: Beverage group in male rats; M_HUPF: High-frequency UPF group in male rats; M_LUPF: Low-frequency UPF group in male rats.

As illustrated in Figure 1B, all groups demonstrated an upward trend for body weight in female and male rats following a 90-day intervention period. However, no statistically significant differences were detected between the groups. Furthermore, fasting blood glucose levels exhibit no significant differences among the rat groups at 1, 30, 60, and 90 days when compared to the control group (*p* > 0.05; Figure 1C). HE staining was performed on liver tissue to assess the histopathological changes in liver tissue following the intervention with UPF. The findings demonstrate that the consumption of UPF by both male and female rats resulted in the presence of lipid droplets and steatosis within hepatic cells, as compared to the control group (Figure 1A). The control group exhibited a higher average feed intake and energy intake compared to the HUPF group, with difference values of 3231.19 g and 6628.95 kcal, respectively, and the LUPF group, with difference values of 1496.5 g and 3808.86 kcal, as illustrated in Supplementary Figure S2.

### 3.2 Impact of UPF on biochemical indices, inflammatory factors, and oxidative stress

**Figure 2:**
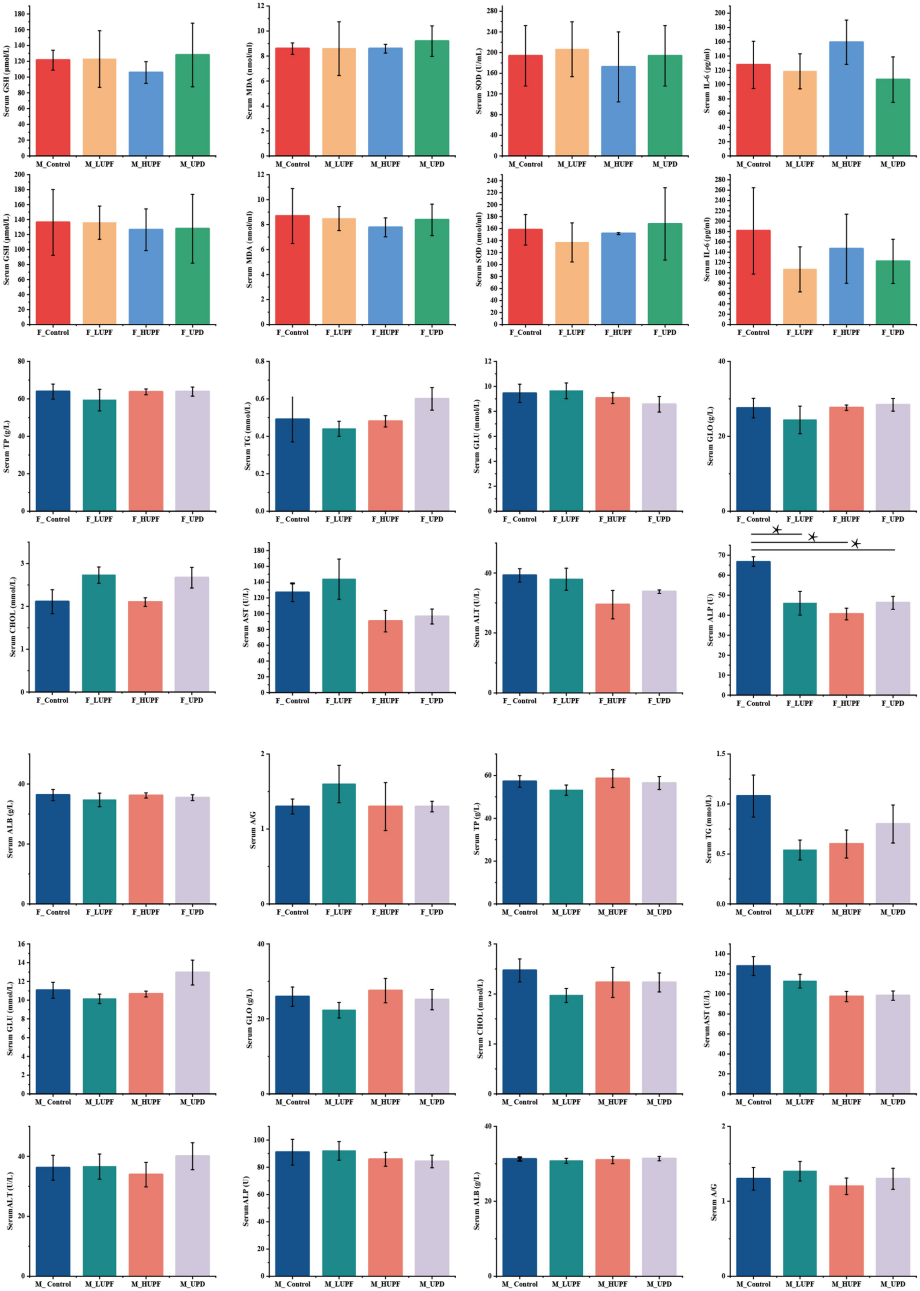
Biochemical indices, inflammatory factors, and oxidative stress. A/G stands for albumin to globulin. Differences between groups were determined using a one-way ANOVA (for normally distributed data with equal variances), followed by Tukey’s multiple comparison test. For non-parametric data, the Kruskal-Wall test was applied, with pairwise comparisons conducted using Dunn’s Test, *p < 0.05 represent compared with the control group. F_Control: Control group in female rats; F_UPD: Beverage group in female rats; F_HUPF: High-frequency UPF group in female rats; F_LUPF: Low-frequency UPF group in female rats; M_Control: Control group in male rats; M_UPD: Beverage group in male rats; M_HUPF: High-frequency UPF group in male rats; M_LUPF: Low-frequency UPF group in male rats.

As illustrated in Figure 2, in female rats, the serum level of ALP was significantly reduced across all three intervention groups in comparison to the control group (*p* < 0.05). Conversely, no significant differences were observed in the serum level of TP, ALB, GLO, ALT, CHOL, AST, GLU, A/G, and TG among the three intervention groups relative to the control group (*p* > 0.05). In male rats, no significant differences were found in the serum level of TP, ALB, TG, GLO, ALT, CHOL, GLU, AST, A/G, and ALP among the intervention groups compared to the control group (*p* > 0.05). Additionally, serum concentrations of IL-6, GSH, MDA, and SOD showed no significant changes across the groups in both male and female rats (*p* > 0.05).

### 3.3 Impact of UPF on gut microbiome community diversity and richness

**Figure 3:**
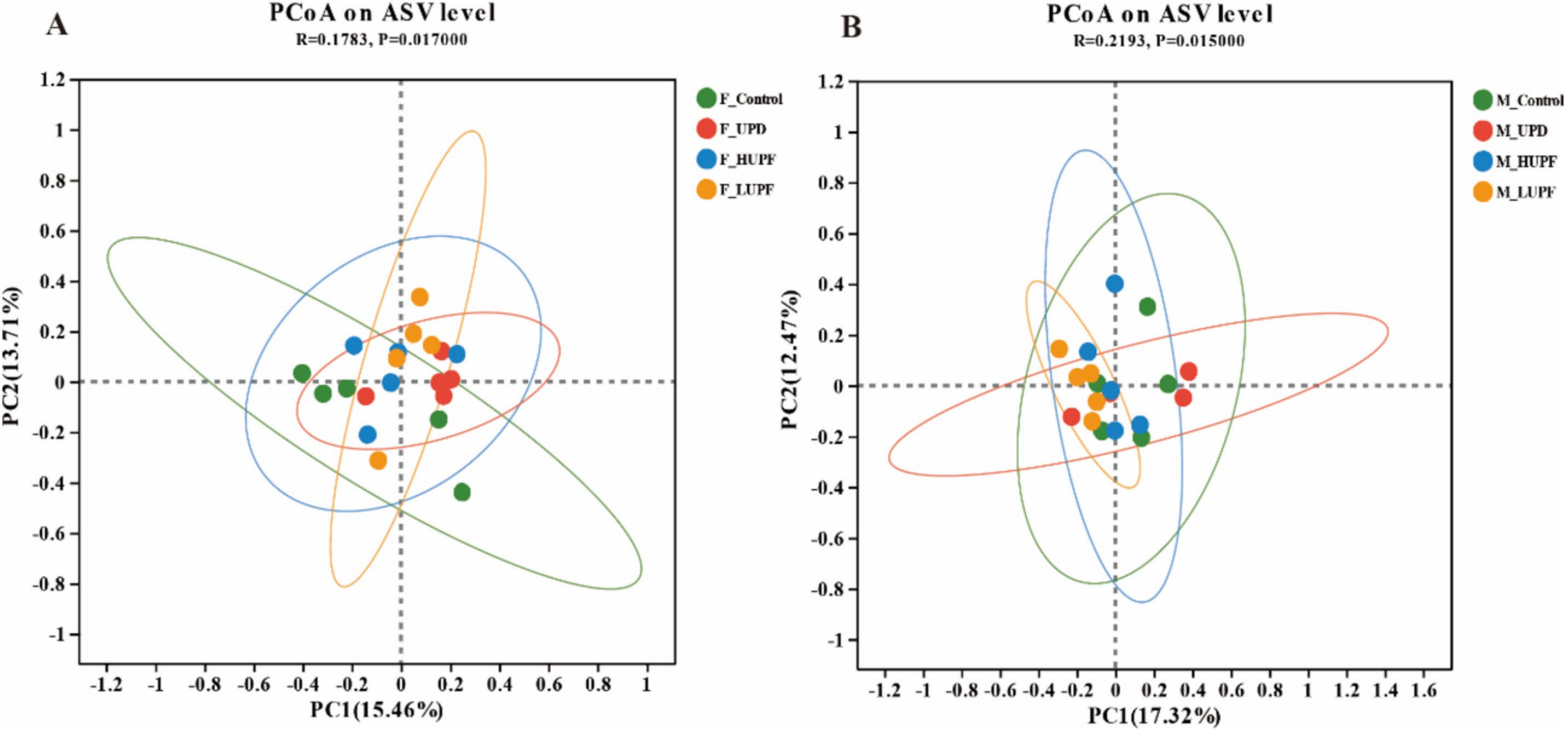
**(A)** PCoA based on ASV level in female rats. **(B)** PCoA based on ASV level in male rats. F_Control: Control group in female rats; F_UPD: Beverage group in female rats; F_HUPF: High-frequency UPF group in female rats; F_LUPF: Low-frequency UPF group in female rats; M_Control: Control group in male rats; M_UPD: Beverage group in male rats; M_HUPF: High-frequency UPF group in male rats; M_LUPF: Low-frequency UPF group in male rats.

The impact of UPF exposure on the gut microbiome was evaluated using 16S rRNA sequencing. To quantify community diversity and richness, alpha diversity metrics were employed. The analysis of alpha diversity showed no significant differences in each group at the ASV level (*p >* 0.1), with detailed results available in the Supplementary Table S2. Principal coordinate analysis (PCoA) of similarity and permutational multivariate analysis of variance (PERMANOVA, 9999 permutations) were conducted to measure the *β*-diversity. As depicted in Figures 3A,B, the samples clustered within each group and were separated between different groups (*p* < 0.05).

### 3.4 Impact of UPF on compositions of the gut microbiome

The optimized sequences obtained from 39 subjects produced a total of 3,149,671 sequences, encompassing 1,306,334,472 bases, which corresponds to an average sequence length of 415 base pairs. Taxonomic classification of the species annotation results indicated the presence of the following categories: Domain: 1; Kingdom: 1; Phylum: 11; Class: 18; Order: 52; Family: 97; Genus: 226; Species: 423; and Amplicon Sequence Variants (ASVs): 22,043. To identify key characteristics of the gut microbiota, ranging from phylum to genus. Linear discriminant analysis effect size (LEfSe) was employed (LDA score > 2.0). At the genus level, the predominant bacterial taxa identified in the control group include *Fournierella, Anaeroplasma, Candidatus_Saccharimona, Rikenellaceae_RC9_gut_group, Butyricimonas, nclassified_f__Erysipelatoclostridiaceaeandu, Lachnospiraceae_UCG-001,* among other. In contrast, the interventional group was characterized by the presence of *Allobaculum, Dubosiella, nclassified_c__Bacilli, unclassified_c__Clostridia, Ruminiclostridium,* and others. This is illustrated in Supplementary Figure S3.

**Figure 4:**
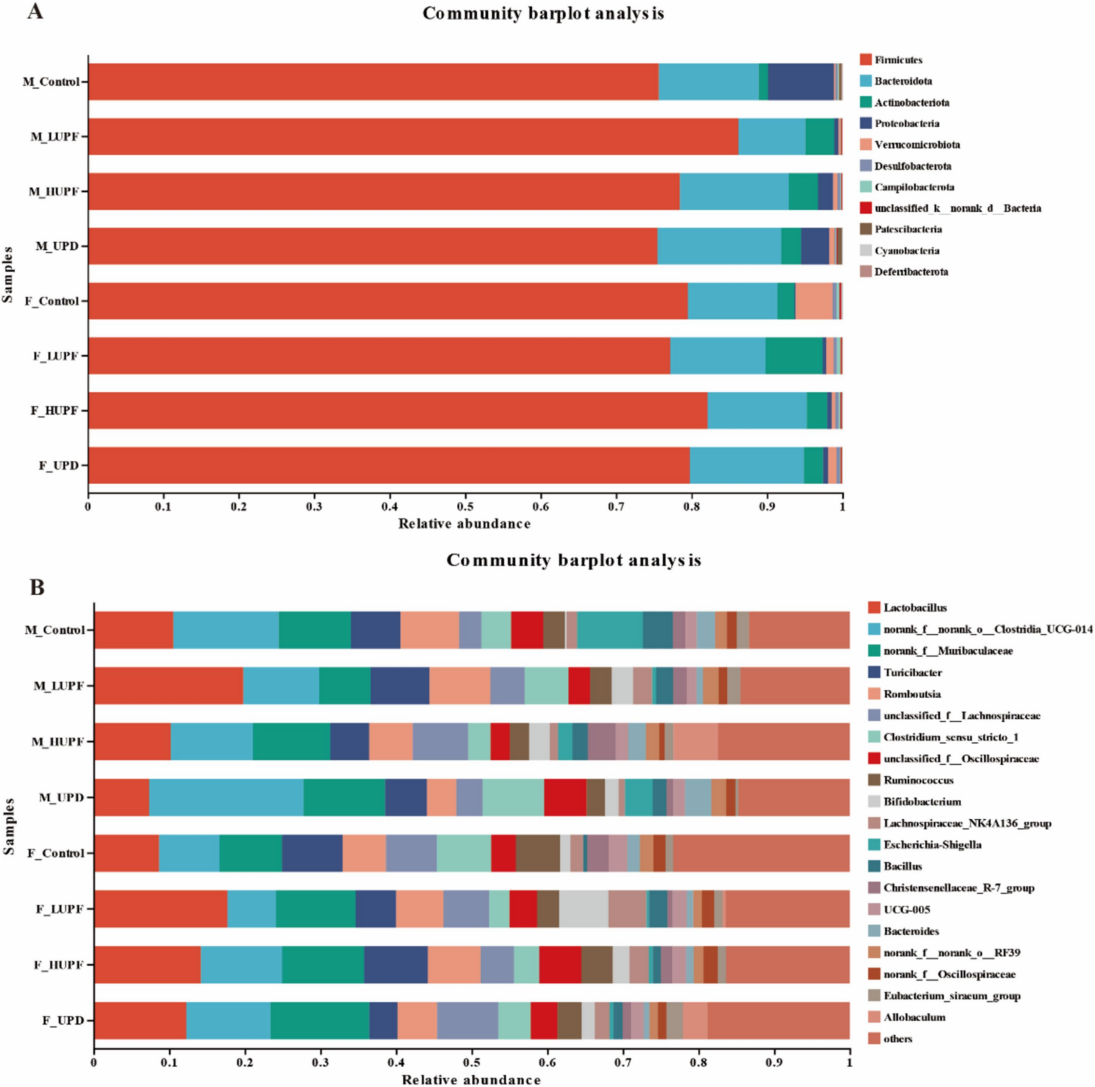
Alterations in gut microbiota composition in male and female rats. **(A)** At the Phylum level, **(B)** At the Genus level. F_Control: Control group in female rats; F_UPD: Beverage group in female rats; F_HUPF: High-frequency UPF group in female rats; F_LUPF: Low-frequency UPF group in female rats; M_Control: Control group in male rats; M_UPD: Beverage group in male rats; M_HUPF: High-frequency UPF group in male rats; M_LUPF: Low-frequency UPF group in male rats.

The alterations in specific bacterial groups following the UPF intervention are depicted in Figure 4, which depicts the community composition at both the phylum and genus levels for each group. *Firmicutes and Bacteroidota* remained the dominant bacterial phyla, with their relative abundances varying among the groups. The *Firmicutes* to *Bacteroidota* (F/B) ratio was elevated was lower in the F_LUPF group (6.09), F_HUPF group (6.26), and F_UPD group (5.28) in comparison to the F_Control group (6.68). The F/B ratio was observed to be higher in the M_LUPF group (9.07) compared to the M_Control group (5.79), while it was lower in both the M_UPD group (4.58) and the M_HUPF group (5.43) relative to the M_Control group (5.79). We concentrated on the top eight bacterial genera based on their relative abundance at the genus level, with some of these listed in Supplementary Table S3.

**Figure 5:**
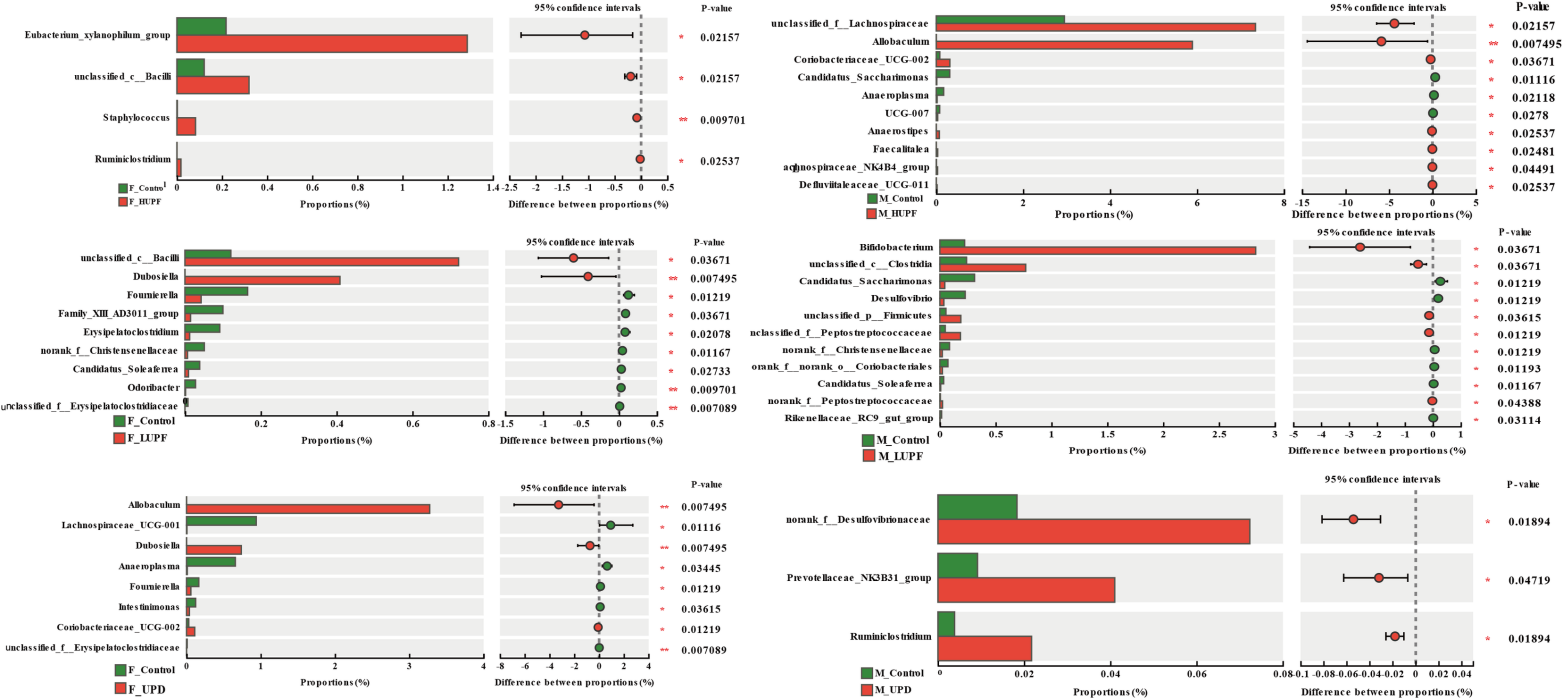
Genus-level bacteria showing notable shifts in relative abundance across different groups in both male and female rats. F_Control: Control group in female rats; F_UPD: Beverage group in female rats; F_HUPF: High-frequency UPF group in female rats; F_LUPF: Low-frequency UPF group in female rats; M_Control: Control group in male rats; M_UPD: Beverage group in male rats; M_HUPF: High-frequency UPF group in male rats; M_LUPF: Low-frequency UPF group in male rats.

Using the Wilcoxon rank sum test, we identified significant differences in gut microbiota composition among the rat groups at the genus level. As illustrated in Figure 5, in female rats, the abundance of *Eubacterium xylanophilum group, unclassified c__Bacilli, Staphylococcus*, and *Ruminiclostridium* increased significantly in the HUPF group. It was found that the LUPF group had significantly increased levels of *unclassified c__Bacilli* and *Dubosiella*, while the experiencing reductions in *Fournierella, Family_XIII_AD3011_ group,* and *Erysipelatoclostridium.* There was an increase in *Allobaculum, Dubosiella,* and *Coriobacteriaceae UCG-002* in the UPD group, whereas *Lachnospiraceae UCG-001, Anaeroplasma*, and *Fournierella* showed significant decreases. In male rats, the HUPF group exhibited a significant increase in *Unclassified_f_Lachnospiraceae* and *Allobaculum*, while *Candidatus_Saccharimonas* and *Anaeroplasma* were significantly decreased. Conversely, the LUPF group showed significant increases in *Bifidobacterium* and *unclassified c__Clostridia*. Additionally, the UPD group showed significant increases in *unclassified f__Desulfovibrionaceae* and *Ruminiclostridium.*

### 3.5 Impact of UPF on the metabolome

Untargeted metabolomic analysis of fecal samples was performed using LC–MS, resulting in the identification of 4,938 annotated compounds through the integration of primary and secondary mass spectrometry data and comprehensive library searches. The analysis employed two ESI+ and ESI- ionization modes, detecting 3,099 metabolites in the ESI+ mode and 1,839 metabolites in the ESI- mode.

**Figure 6:**
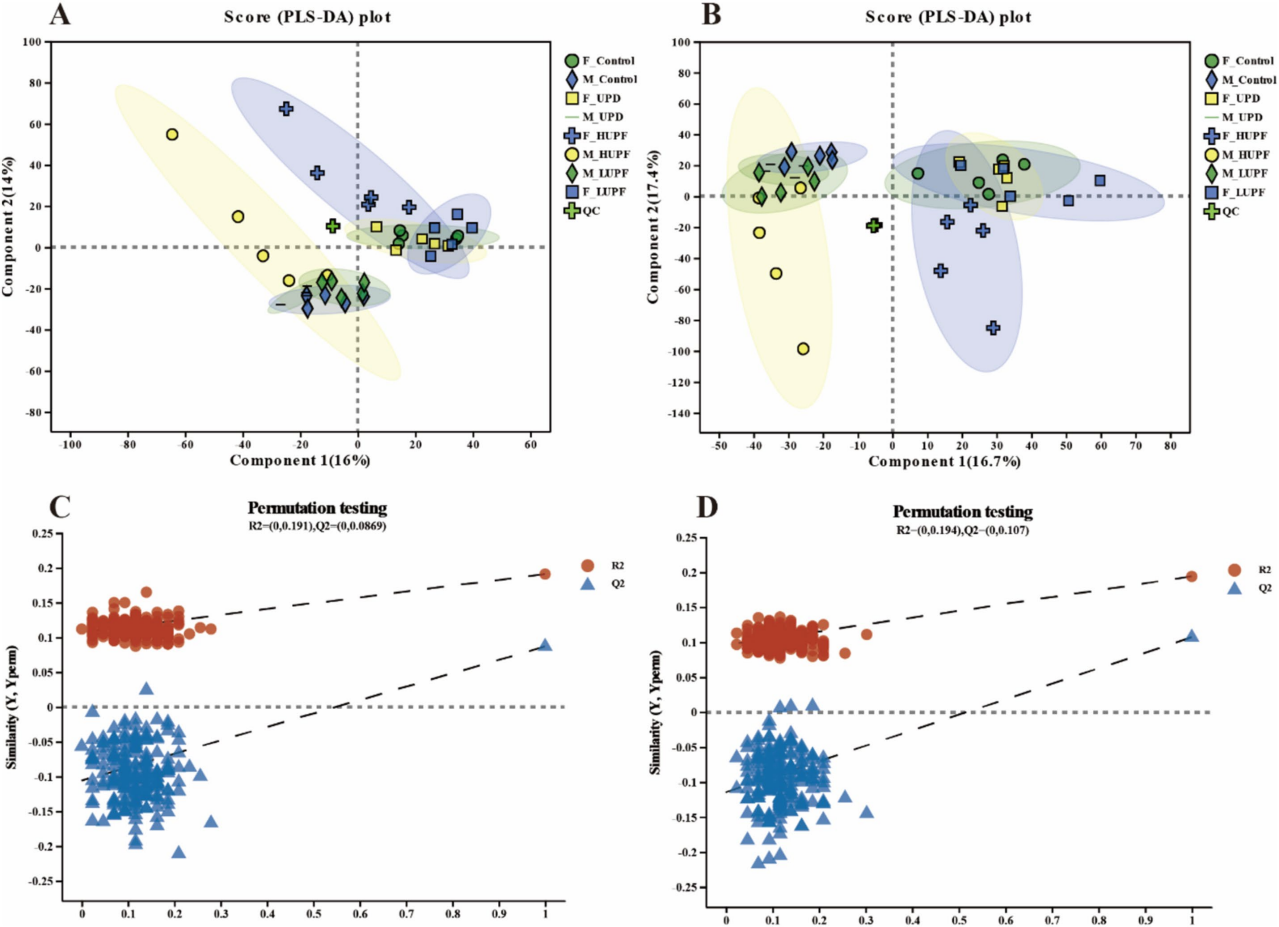
PLS-DA score plots for samples across groups in the ESI+ model **(A)** and ESI- model **(B)** for male and female rats. PLS-DA permutation tests for the positive ion mode **(C)** and negative ion mode **(D)**. F_Control: Control group in female rats; F_UPD: Beverage group in female rats; F_HUPF: High-frequency UPF group in female rats; F_LUPF: Low-frequency UPF group in female rats; M_Control: Control group in male rats; M_UPD: Beverage group in male rats; M_HUPF: High-frequency UPF group in male rats; M_LUPF: Low-frequency UPF group in male rats.

PLS-DA of all detected metabolites demonstrated strong clustering near the origin of the QC samples plot, thereby, confirming the stability and reproducibility of the instrument, as shown in Figure 6. Moreover, a clear separation was observed between the control groups and other experimental groups in both the ESI+ and ESI- modes, with sample points within each group concentrated within a 95% confidence interval. The validation of the PLS-DA model demonstrated that the R^2^ values in both ionization modes exceeded Q^2^, and the intercepts of the Q^2^ regression line on the vertical axis (Y) were below zero. This indicates a well-fitting model with high predictive accuracy, making it suitable for further data analysis.

**Figure 7:**
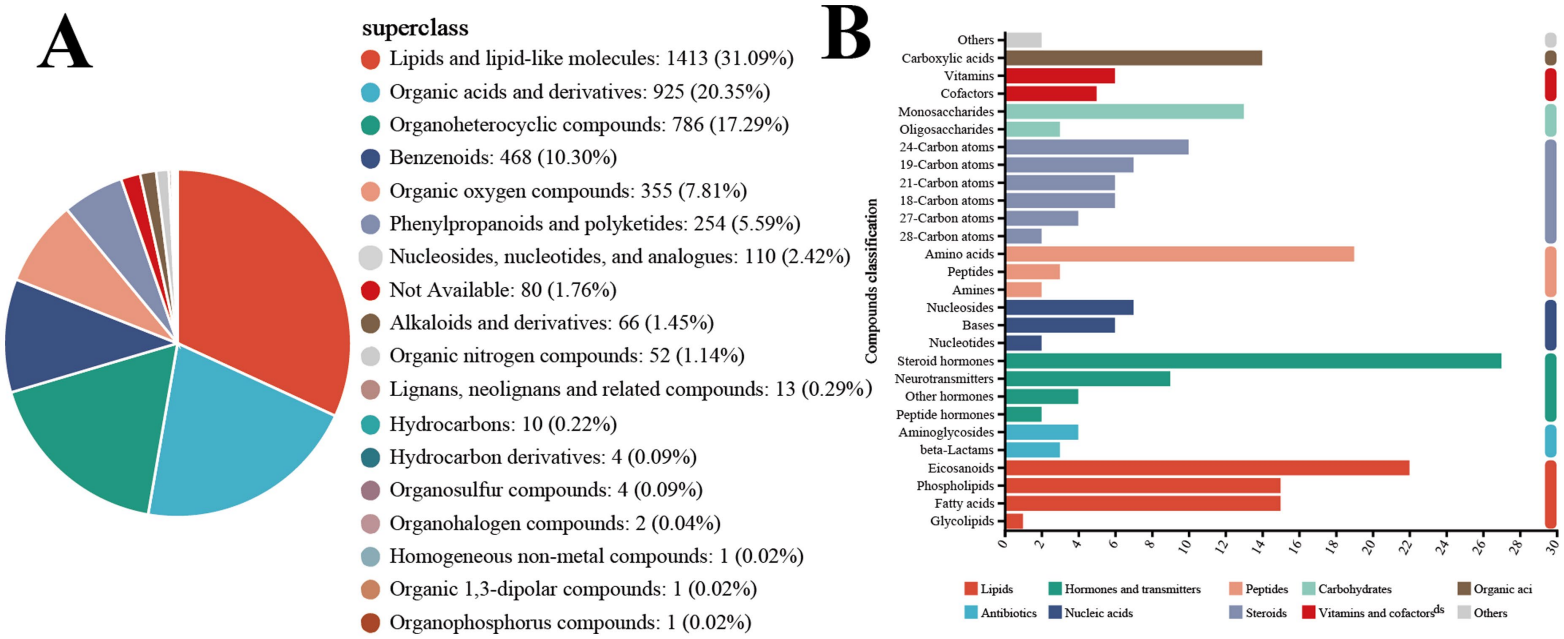
**(A)** KEGG compound classification. **(B)** HMDB compound classification.

Subsequently, the classification and annotation of identified metabolites were performed using the KEGG and HMDB databases to elucidate the biological pathways and functions associated with each metabolite, as shown in Figure 7. The KEGG Compound classification organizes metabolites by compounds with biological roles, with Lipids being the largest group, comprising 53 compounds. Following the lipids, steroids accounted for 35 compounds, hormones and transmitters for 42, nucleic acids for 15, peptides for 24, carbohydrates for 16, and finally, vitamins and cofactors for 11. In HMDB 4.0 compounds are categorized according to the Superclass hierarchy. The primary categories include lipids and lipid-like molecules at 31.09% (1,413 compounds), Organic acids and derivatives at 20.35% (925 compounds), and organoheterocyclic compounds representing 17.29% (768 compounds). These are followed by benzenoids at 10.30%, and organic oxygen compounds at 7.81%.

**Figure 8:**
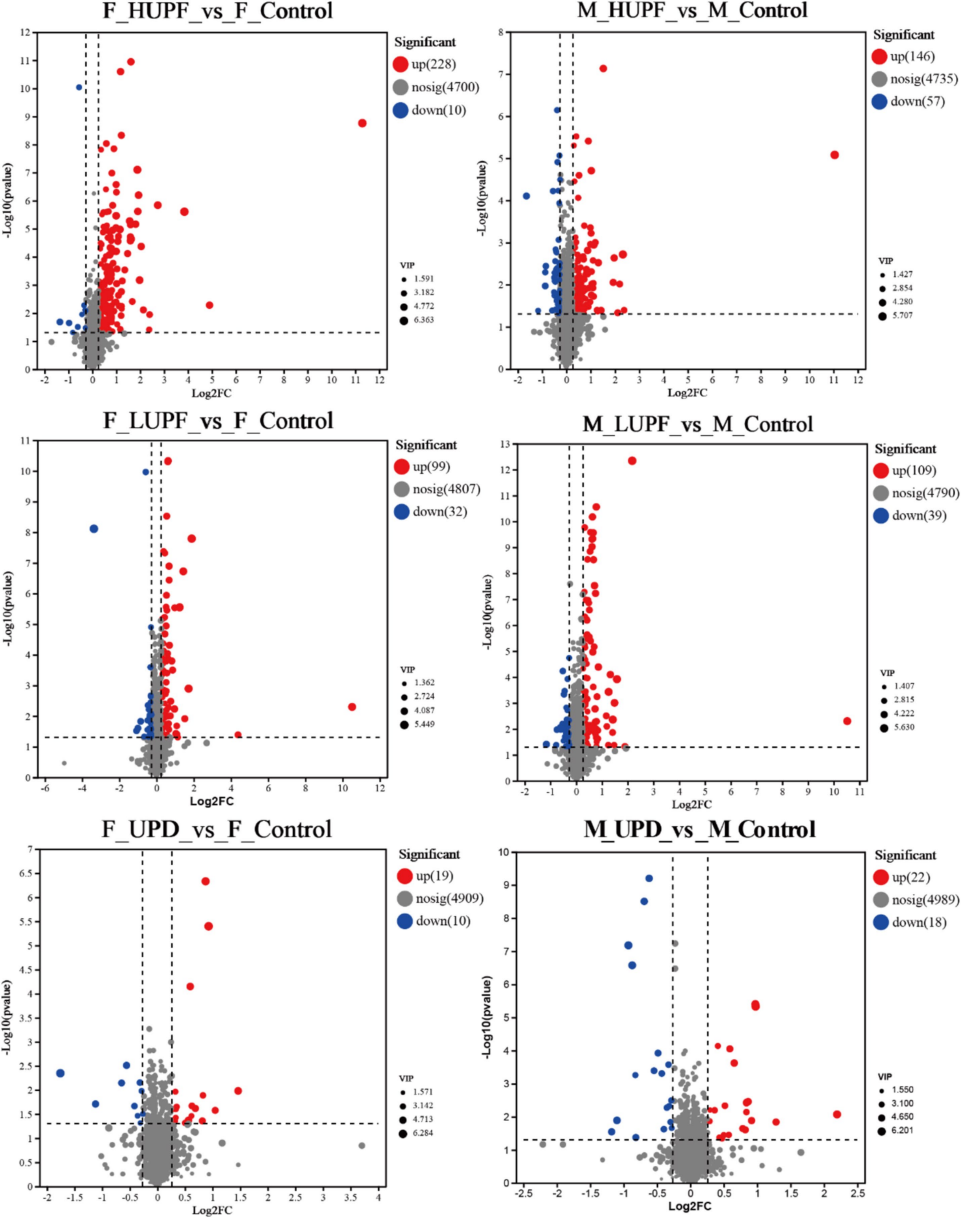
Volcano map of metabolites in male and female rats. F_Control: Control group in female rats; F_UPD: Beverage group in female rats; F_HUPF: High-frequency UPF group in female rats; F_LUPF: Low-frequency UPF group in female rats; M_Control: Control group in male rats; M_UPD: Beverage group in male rats; M_HUPF: High-frequency UPF group in male rats; M_LUPF: Low-frequency UPF group in male rats.

The metabolites that exhibited differences among various UPF groups were further analyzed using variable importance in projection (VIP) as a threshold in this study (21). Metabolites that satisfied the criteria of *p* < 0.05, VIP > 1, and a fold change of ≤0.83 or ≥ 1.2 were identified as differential metabolites, as shown in Figure 8. In female rats, the UPD group revealed 29 differential metabolites, with 10 exhibiting decreased levels and 19 increased levels. In contrast, in the HUPF group, 238 differential metabolites were found, comprising 10 that were downregulated and 228 upregulated metabolites. The LUPF group demonstrated 131 differential metabolites, with 32 downregulated and 99 upregulated. Similarly, in male rats, the UPD group showed 40 differential metabolites, with 18 metabolites downregulated and 22 upregulated. The HUPF group revealed 203 differential metabolites, including 56 downregulated and 146 upregulated metabolites. Additionally, the LUPF group showed 148 differential metabolites, with 39 metabolites downward and 109 metabolites upward. Detailed results of the differential metabolite screening for each group are presented in Supplementary Table S4.

**Figure 9:**
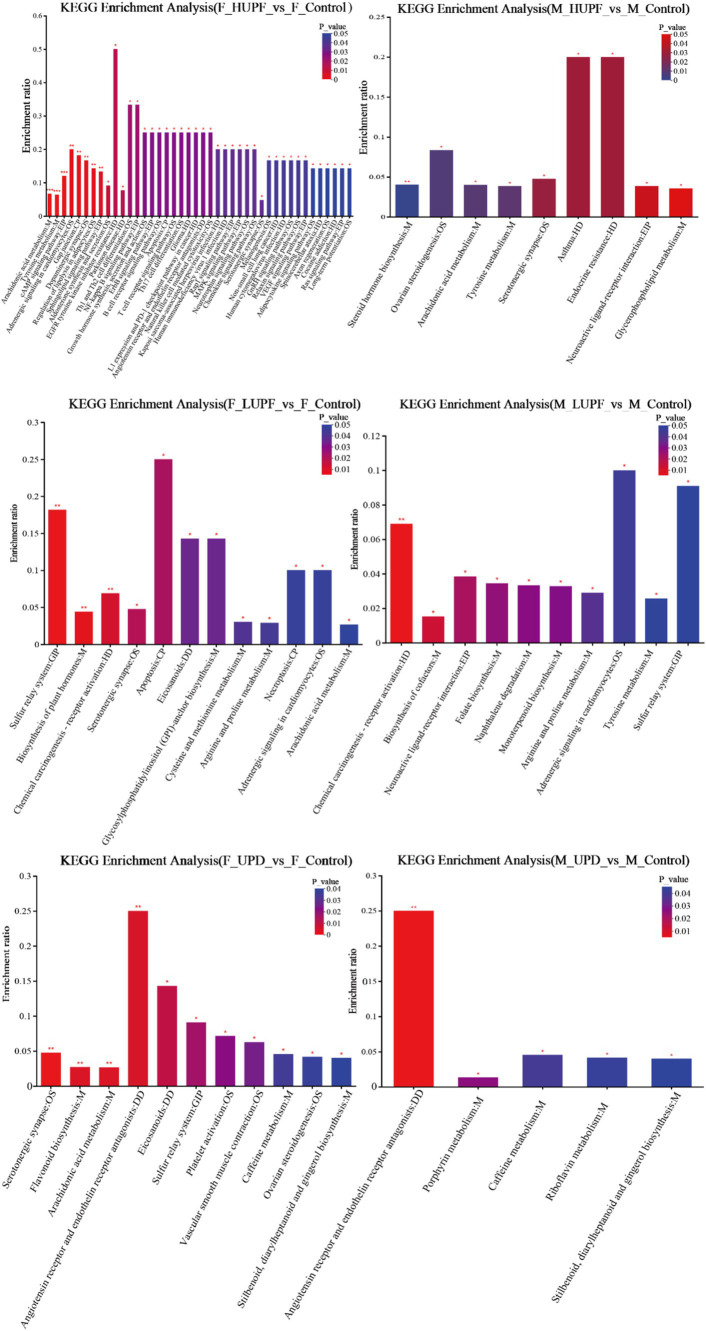
KEGG enrichment analysis across different groups in male and female rats. F_Control: Control group in female rats; F_UPD: Beverage group in female rats; F_HUPF: High-frequency UPF group in female rats; F_LUPF: Low-frequency UPF group in female rats; M_Control: Control group in male rats; M_UPD: Beverage group in male rats; M_HUPF: High-frequency UPF group in male rats; M_LUPF: Low-frequency UPF group in male rats.

To further elucidate the metabolic pathways associated with UPF, the KEGG database was utilized to input the differential metabolites for pathway construction and analysis, as illustrated in Figure 9. In male rats, UPF intervention significantly enriched several metabolic pathways of Vascular smooth muscle contraction, Tyrosine metabolism, Sulfur relay system, Stilbenoid, diarylheptanoid, and gingerol biosynthesis, Steroid hormone biosynthesis, Serotonergic synapse, Platelet activation, Ovarian steroidogenesis, Neuroactive ligand-receptor interaction, Naphthalene degradation, Monoterpenoid biosynthesis, Glycerophospholipid metabolism, Folate biosynthesis, Arachidonic acid metabolism, Angiotensin receptor and endothelin, Adrenergic signaling in cardiomyocytes and so on. In female rats, the UPD group exhibited significant enrichment in 11 metabolic pathways, including Serotonergic synapse, Stilbenoid, diarylheptanoid, and gingerol biosynthesis, Arachidonic acid metabolism, Caffeine metabolism, Eicosanoids, Flavonoid biosynthesis, Sulfur relay system, Ovarian steroidogenesis, Angiotensin receptor and endothelin receptor antagonists, Vascular smooth muscle contraction, and Platelet activation. In addition, there was a significant concentration in the LUPF group in 12 metabolic pathways including Serotonergic synapse, Cysteine and methionine metabolism, Apoptosis, Arachidonic acid metabolism, Necroptosis, Arginine and proline metabolism, Eicosanoids, Sulfur relay system, Glycosylphosphatidylinositol (GPI)-anchor biosynthesis, Adrenergic signaling in cardiomyocytes, Biosynthesis of plant hormones, and Chemical carcinogenesis - receptor activation. In examining the HUPF group 42 metabolic pathways were enriched, as follows: Tyrosine metabolism, Arachidonic acid metabolism, Serotonergic synapse, Sphingolipid signaling pathway, Angiotensin receptor and endothelin receptor antagonists, Regulation of lipolysis in adipocytes and other related pathways.

### 3.6 Potential correlations between the gut microbiota and metabolites

**Figure 10:**
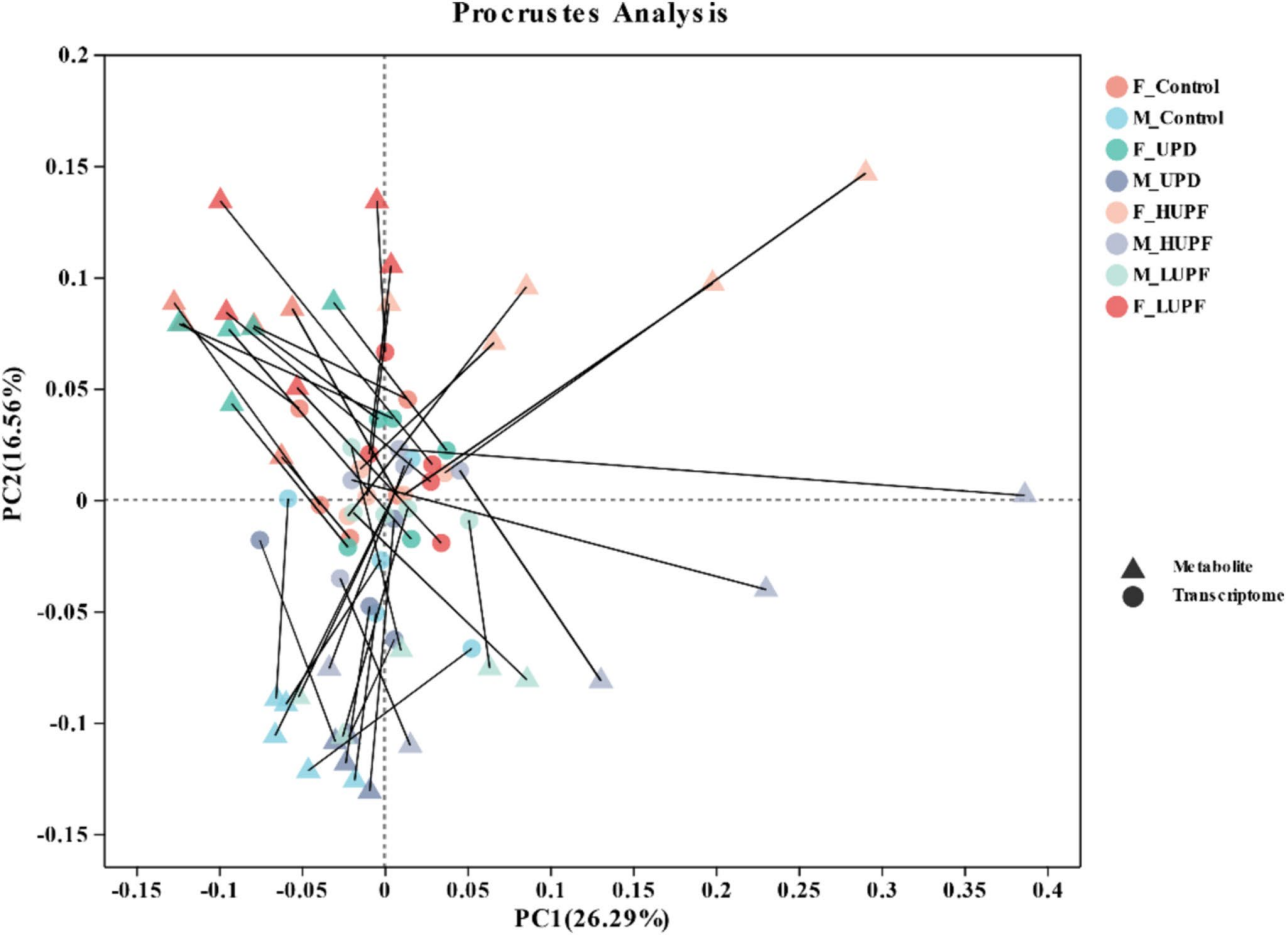
Procrustes analysis of gut microbiome and metabolomics.

We employed the Procrustes correlation to assess the association between metabolite and microbiota data at the genus level, as shown in Figure 10. The analysis yielded a statistically significant result (Monte Carlo *p*-value = 0.036), indicating that fecal metabolites function as substrates or byproducts of the microbiota.

**Figure 11:**
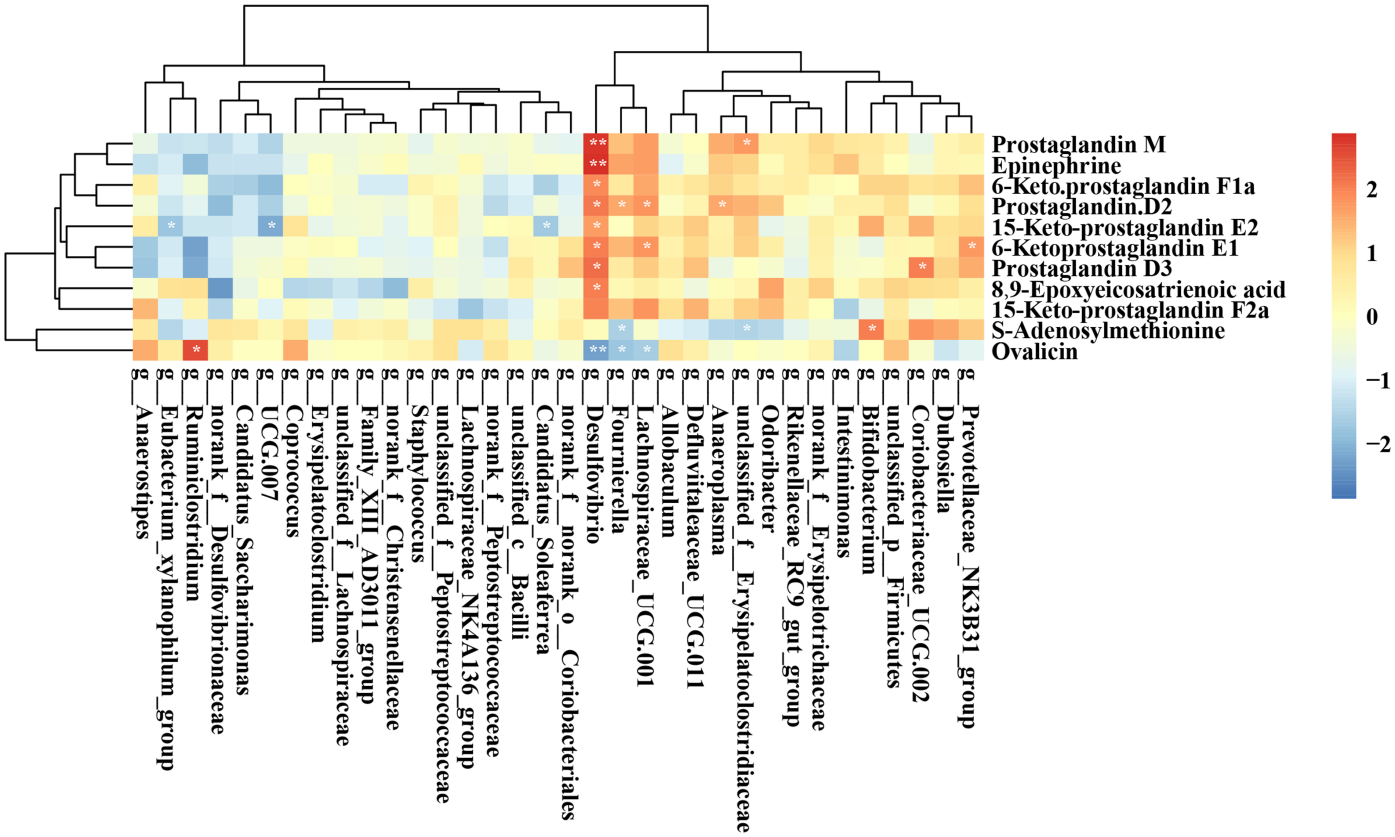
Heatmap displaying the correlation between the abundances of key bacteria and key metabolites. *p < 0.05; **p < 0.01; ***p < 0.001.

Figure 11 illustrates the correlation between key bacteria and key metabolites in rats. *Desulfovibrio* exhibited a significant positive correlation with several metabolites, including Prostaglandin M, Epinephrine, 6-keto prostaglandin E1, 15-keto-prostaglandin E2, Prostaglandin D2, Prostaglandin D3, 6-keto-prostaglandin F1a, and 8,9-Epoxyeicosatrienoic acid. Additionally, *Ruminiclostridiu*m demonstrated a significant positive association with *Ovalicin.* While *Bifidobacterium* showed a significant positive association with S-Adenosylmethionine.

### 3.7 Potential correlations between the gut microbiota and serum biochemical indices

**Figure 12:**
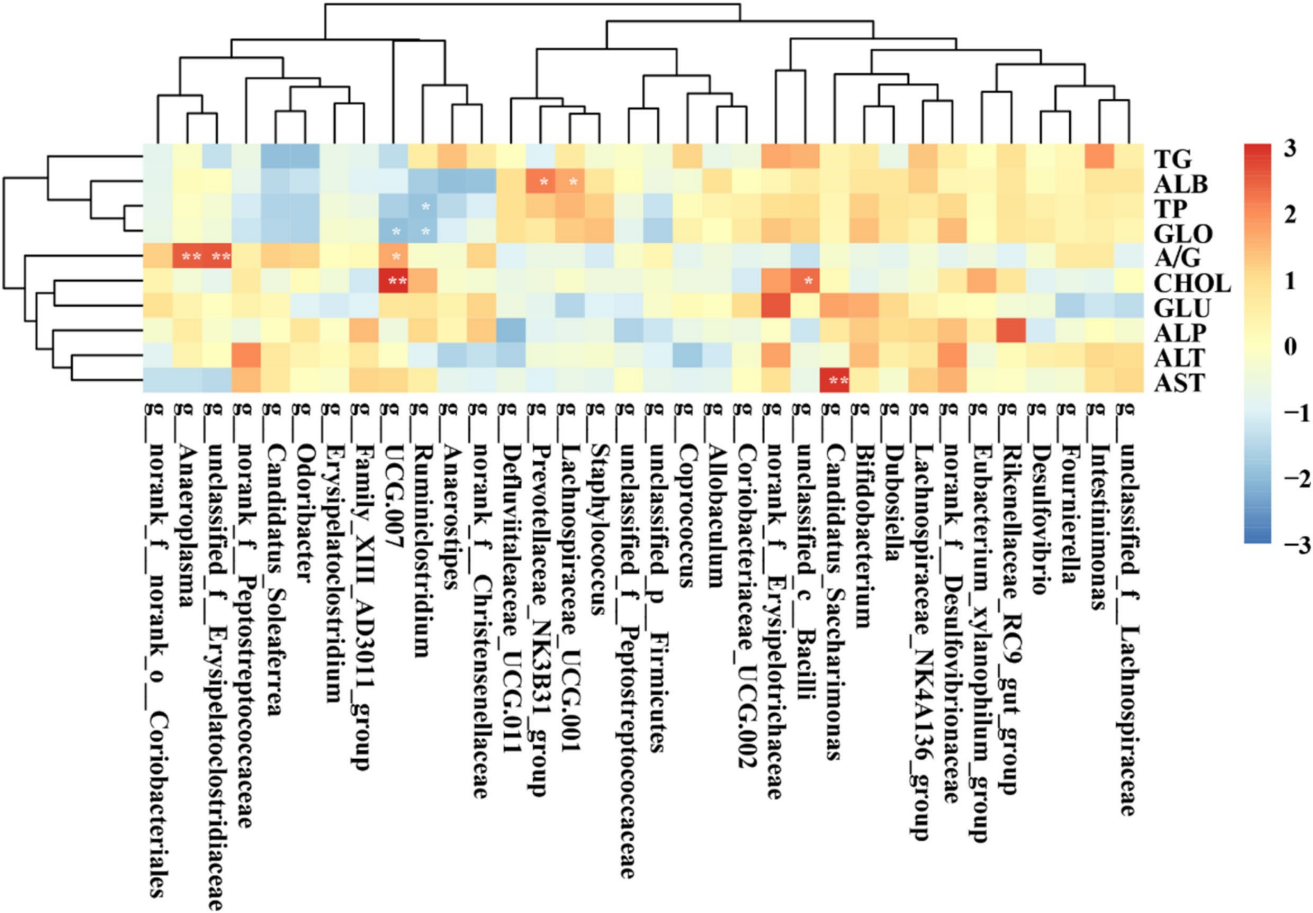
Heatmap showing the correlation between the abundances of key gut microbiota and serum markers. *p < 0.05; **p < 0.01; ***p < 0.001.

Figure 12 illustrates the correlation between key gut microbiota and serum marker parameters in rats. Specifically, *unclassified_c__Bacilli* and *UCG 007* were significantly positively correlated with CHOL, while *Candidtus_Saccharimonas* demonstrated a significant positive association with AST. *Ruminiclostridium* showed a significant negative correlation with TP and GLO.

## 4 Discussion

In our research, we simulated modern Chinese eating habits by intermittently feeding SD rats with UPF for 90 days to explore the impact of UPF on liver health. The results indicate that short-term and intermittent oral exposure to UPF induces hepatic alterations, possibly linked to alterations in intestinal microbial composition and metabolites.

UPF poses a global threat to public health and contributes to the overall disease burden. A prospective analysis of older adults with metabolic syndrome, utilizing data from the PREDIMED-Plus trial, revealed that increased UPF consumption is correlated with heightened levels of biomarkers indicative of NAFLD in individuals who are overweight or obese and have metabolic syndrome (11). Furthermore, a prospective cohort study involving United Kingdom Biobank participants demonstrated that high UPF intake is associated with detrimental liver outcomes, resulting in elevated levels of ALP, AST, *γ*-glutamyl transferase, and TG as well as decreased cholesterol (22). In this study, rats were fed UPF either three times, once every 10 days, or once every 6 days over 90 days to assess the effects on liver health. HE staining indicated that rats consuming UPF developed hepatocyte steatosis. Despite the observed accumulation of liver fat in UPF-fed rats, there were no statistically significant differences in the levels of TP, ALB, GLO, ALT, CHOL, GLU, AST, and A/G compared to the control group in both female and male rats. NAFLD is primarily characterized by fat accumulation in hepatocytes, which can lead to hepatocyte damage, oxidative stress, inflammation, insulin resistance, and subsequent pathological cascades, representing a crucial transitional stage for NAFLD to advance to cirrhosis and liver cancer (23). Hepatic steatosis, characterized by the accumulation of lipid droplets within liver parenchyma, serves as a precursor to the inflammatory processes observed in steatohepatitis, fibrosis, and end-stage liver disease. The accumulation of lipids in hepatocytes can disrupt the metabolism of xenobiotics and endogenous compounds, while simultaneously activating cellular mechanisms that facilitate (24). Previous studies have indicated that steatosis can be observed in diseases induced by high-fat diets and dietary protein deficiencies (12, 25). In NAFLD, lipotoxicity occurs when the influx of free fatty acids (FFAs) surpasses the liver’s capacity to metabolize, store, or export them, resulting in mitochondrial and endoplasmic reticulum stress, hepatocyte apoptosis, and the release of pro-inflammatory cytokines (26). This study found no significant differences in fasting blood glucose levels, IL-6, GSH, MDA, and SOD between the experimental groups and the control group in both male and female rats. A study examining the morphological and histopathological changes in the livers of mice subjected to a high-fat diet throughout 1 to 12 months indicated that microvesicular steatosis of hepatocytes was evident after 1 to 2 months without lobular inflammation. Notably, after 4 months, there was a significant increase in hepatocyte fat accumulation, accompanied by hepatocellular ballooning and intralobular inflammation occurring locally (27). Thus, the lack of inflammatory cytokines may be attributed to the early stage of NAFLD. In the experimental murine model fed a Western diet (WD), significant lipid accumulation and inflammation were observed by the eighth week (28). Previous studies have also proposed that NAFLD lesions initially manifest as simple steatosis, followed by non-alcoholic steatohepatitis (NASH) (27). The present study’s findings indicate that the livers of the rats, irrespective of sex, are likely still in the simple steatosis stage and have not advanced to NASH. Future studies with extended intervention durations are necessary to ascertain whether UPF can induce inflammatory responses.

Emerging evidence suggests that alterations in gut microbiome composition may contribute to the onset of obesity and its related metabolic disorders, such as disruptions in liver lipid metabolism and lipid distribution abnormalities (29). This study assessed the impact of UPF on the gut microbiota composition in rats using 16S rRNA sequencing. Notably, there was a significant increase in the abundance of *norank_f__Desulfovibrionaceae* in the M_UPD group compared to the M_Control group. *Desulfovibrionaceae (Desulfovibrionaceae family)* is the primary sulfate-reducing bacteria in the gut, typically converting sulfate into hydrogen sulfide (H₂S) (30) and metabolizing choline into trimethylamine (TMA), which is subsequently converted into trimethylamine-N-oxide (TMAO) by hepatic monooxygenase enzymes. The presence of TMAO is associated with adverse health effects, including the promotion of hyperlipidemia and the development of fatty liver disease (31). *Staphylococcus* has been identified as an important pathogen in patients with chronic liver diseases and is closely associated with the development of cystic fibrosis (32). It is significantly enriched in the F_HUPF group compared to the F_Control group. *Coriobacteriaceae UCG-002 (Coriobacteriaceae)* could produce phenol and cresol, which could promote intestinal inflammation and epithelial permeability, compounds are cytotoxic and weaken intestinal barrier function (33). This bacterium has significant abundance in the M_ HUPF group compared to the M_Control group. The F/B ratio is generally recognized as playing a crucial role in preserving normal intestinal homeostasis (34). Several studies have indicated that a lower F/B ratio is linked to NASH (35). The intake of UPF can reduce the F/B ratio in female rats. Interestingly, the abundances of short-chain fatty acid (SCFA)-producing species, including *Allobaculum*, *Dubosiella*, *unclassified_f_Lachnospiracea*, *Lachnospiraceae UCG-001*, *unclassified_c_Bacilli*, *[Eubacterium]_xylanophilum group*, *Provotellaceae _NK3B31_group*, and *Ruminiclostridium* were significantly increased following the introduction of UPF across various groups. SCFA play a crucial role in maintaining intestinal barrier integrity, offering anti-inflammatory benefits, and enhancing glucose tolerance and insulin sensitivity by exerting positive effects on liver and adipose tissue function (36). *Allobaculum (Erysipelotrichaceae)* is known to produce butyric acid, and recent research reveals that it is abundant in the gut microbiome of mice fed a Western diet. Furthermore, *Allobaculum* shows a positive correlation with increased mRNA and protein expression levels of angiogenin-like protein 4 in NAFLD, suggesting a potential involvement in the gut-liver axis (37). Notably, its abundance is significantly increased in the F_UPD group and M_HUPF group. Additionally, research by Li et al. has indicated a marked increase in the abundance of *unclassified_f_Lachnospiraceae* in a mouse model exhibiting glucose metabolic disorders induced by a high-fat diet (38). *Dubosiella (Erysipelotrichaceae family)* has been shown to induce glucose intolerance in the host by producing homocysteine (39). Furthermore, a significant increase in *Dubosiella* populations has been observed in mice with colitis and those fed a high-fat diet (HFD) (40). The *Provotellaceae _NK3B31_group (Bacteroidales)* is an acetic acid-producing genus. Studies have suggested that the *Prevotellaceae_NK3B31_group* is associated with anti-inflammatory effects (41). Despite alterations in dietary habits or substantial disruptions, the gut microbiota generally preserves relative stability and is capable of restoring its functional state in a healthy host, attributed to its remarkable capacity for self-regeneration, a phenomenon termed resilience (42). These findings indicate that UPF disrupts the composition of the gut microbiome, leading to an increase in the abundance of both harmful and potentially beneficial bacteria. Nevertheless, owing to the microbiota’s resilience, there is an observed increase in bacteria that produce SCFA. The long-term effects of UPF on the gut microbiome remain to be fully elucidated.

UPF intake has the potential to disrupt metabolic processes through alterations in the gut microbiota, which generates a range of bioactive metabolites that can affect liver health. LC–MS is a powerful tool for identifying specific metabolites and predicting metabolic pathways associated with disease phenotypes. This technique facilitates the elucidation of potential mechanistic links through comprehensive analyses integrating the microbiome, metabolome, and host phenotypes (43). The metabolites that exhibited significant differences among these groups were predominantly associated with lipid metabolism (including arachidonic acid metabolism, glycerophospholipid metabolism, and steroid hormone biosynthesis), amino acid metabolism (specifically tyrosine metabolism and arginine and proline metabolism), the biosynthesis of other secondary metabolites (such as stilbenoid, diarylheptanoid, and gingerol biosynthesis, caffeine metabolism, and flavonoid biosynthesis), the sphingolipid signaling pathway, the sulfur relay system, and other metabolic pathways.

The liver plays a crucial role in the metabolism of S-adenosylmethionine (SAM), the primary biological methyl donor produced in all mammalian cells (44). SAM is a pivotal sulfonium that participates in various biochemical processes, including transmethylation, transsulfuration, and polyamine synthesis. In animal studies, SAM has consistently been implicated in NAFLD, but elucidating the molecular mechanisms is challenging due to SAM’s involvement in numerous metabolic pathways (45). A decreased level of SAM is a risk factor for liver injury and exacerbation of chronic cirrhosis (46). Previous research has found that reduced SAM levels in the alcohol-induced rat model of hepatic steatosis are strongly associated with the increased serum alanine aminotransferase level and the degree of liver lipid accumulation, indicating that the decrease of SAM may lead to alcoholic steatosis (47). In our study, we observed a significant upregulation of SAM levels in the M_LUPF, M_UPD, and F_LUPF groups. Thus, we surmise that UPF may disrupt the sulfur relay system, potentially triggering a compensatory mechanism that stimulates the liver to produce more SAM. Nonetheless, additional research is necessary to elucidate the precise mechanisms underlying this effect. Sphingosine, a sphingolipid, plays a key role in the development of NASH and can undergo phosphorylation to form sphingosine-1-phosphate. This process may contribute to the advancement of fibrosis in hepatic injury by promoting bile overproduction (48). In our study, sphingolipid levels were significantly downregulated in both the F_HUPF and F_LUPF groups this downregulation may serve a compensatory role, UPF promotes simple steatosis in the liver and activates sphingosine signaling pathways. The activation of these pathways results in decreased sphingosine levels, which may confer protective effects against further hepatic damage. Prostaglandins (PG), including PGE2, PGF2a, and PGD2, are vital bioactive lipid mediators produced from arachidonic acid (AA) through the involvement of cyclooxygenases 1 and 2 (COX1, COX2) and specific prostaglandin synthases (49). In the context of liver injury, the increased hydrolysis of arachidonic acid (an unsaturated fatty acid) by PLA2 activates COX, resulting in elevated PGE2 levels and related downstream signaling pathways (50). The levels of 15-keto PGE2, a downstream metabolite of PGE2, were increased in the F_LUPF, F_HUPF, M_LUPF, and M_HUPF groups. Catecholamines are implicated in the development of hepatic steatosis. Elevated sympathetic nerve activity has been documented in individuals with metabolic syndrome patients and rodent models of high-fat diet-induced obesity (51, 52). This heightened activity has been linked to increased liver triglyceride levels and lipid droplet accumulation. Lelou et al. have similarly identified disruptions in sympathetic nerve fibers in the livers of mice exhibiting steatosis, as well as in humans with steatohepatitis (53). In our study, we investigated the level of catecholamines (epinephrine) upward in F_LUPF, F_HUPF, M_LUPF, and M_HUPF groups.

Liver pathophysiology exhibits sexual dimorphism, as evidenced by studies demonstrating that males are more likely to experience advanced stages of NAFLD compared to females (54). Furthermore, the interplay between the microbiome and hormonal factors exerts a substantial impact on host metabolism. Research indicates that sex-related differences in gut microbiota are accentuated during enteric infections (55). In particular, the adult male and female mice demonstrate distinct bacterial taxonomic compositions and diversity, irrespective of dietary influences. An analysis of the gut microbiota in patients with enteric infections and their healthy family members revealed that sex significantly affects the overall abundance of microbial taxa. Specifically, females exhibited a marginally higher abundance of Bacteroides, while males showed a slightly elevated presence of Escherichia (56). In our investigation, which concentrates on the initial phases of hepatic steatosis, no significant sex differences were detected. This lack of observed differences may be attributed to the relatively early exposure period utilized in our experimental design. Consequently, further research involving extended exposure durations is necessary to more comprehensively examine the sexually dimorphic aspects of liver pathology.

Another potential mechanism responsible for the association between UPF and liver health may involve the presence of additives (emulsifiers, sodium nitrate, and artificial sweeteners) or food contaminants (trans fats or acrylamide, Microparticles, and nanoparticles, etc), these substances may have detrimental effect on liver health. Emulsifiers, which are extensively utilized by the food industry to improve organoleptic properties and extend the shelf-life, with the most commonly used being lecithin, monoglycerides, carboxymethylcellulose (CMC), etc. could lead to transaminitis, steatosis, and toxicity in the liver of rodents (57). A randomized controlled feeding study demonstrated that the consumption of CMC modestly altered gut microbiota composition, leading to reduced microbial diversity and notable changes in the fecal metabolome. These changes were particularly characterized by decreases in SCFA and free amino acids, which may contribute to the rising prevalence of various chronic inflammatory diseases (58). Furthermore, nanoparticles can be present in food products either intentionally, as a result of food additives or supplements, or unintentionally, due to migration from food packaging (59). The most commonly used microparticles are inorganic compounds of titanium dioxide (TiO_2_), TiO_2_ is absorbed by intestinal epithelial cells and macrophages, triggering the release of pro-inflammatory cytokines (60). Other studies, involving nanoparticle intervention in mice, reported long-term oral exposure to leachate from boiled-water-treated plastic products might have affected the diversity and composition of gut microbiota. Specifically, there was an observed increase in Escherichia-Shigella and Alistipes populations, while Lactobacillus, Parabacteroides, Escherichia-Shigella, and Staphylococcus populations decreased. Furthermore, alterations were observed in the quantity of metabolites and the enrichment of metabolic pathways associated with inflammatory responses and immune function. These changes were accompanied by inflammation and morphological alterations in liver cells (61). It is imperative to investigate the potential effects of combined exposure to UPF on hepatic function.

To the best of our knowledge, this study is the first to employ 16S rRNA and LC–MS analysis to investigate the impact of UPF exposure on intestinal microbiota and their metabolic profiles. However, the present study has several limitations. First, the experimental design involved feeding rats a diet consisting of packaged ready-to-eat potato chips, waffles, pork jerky, melon seeds, and milk tea for 90 days to simulate contemporary Chinese dietary patterns and assess the impact of UPF on liver health. Nonetheless, extended exposure durations and frequencies are required to evaluate long-term liver damage, supported by mechanistic studies to establish causality.

## 5 Conclusion

In conclusion, this study, employing comprehensive 16S rRNA and LC–MS analyses, suggests that short-term consumption of UPF may influences simple hepatic steatosis without inducing oxidative stress or inflammation. This effect may be mediated through alterations in the gut microbiota, characterized by an increase in potentially harmful bacteria, such as *norank_f__Desulfovibrionaceae* and *Staphylococcus*, alongside an elevation in the relative abundance of potentially beneficial bacteria, such as *Dubosiella* and *Allobaculum*. UPF is implicated in metabolomic disorders characterized by disruptions in the sphingolipid signaling pathway, sulfur relay system, and arachidonic acid metabolism. Considering the global concern regarding the rising consumption of UPF and its substantial public health implications, it is imperative to further investigate its effects on liver disease.

## Data Availability

The data analyzed in this study is subject to the following licenses/restrictions: the data presented in this study are available on request from the corresponding author. Requests to access these datasets should be directed to Shulan He, heshulan0954@163.com.
