# Supplementary material for: Effects of ultra-processed foods on the liver: insights from gut microbiome and metabolomics studies in rats
Source: Front Nutr. 2025 Jan 22;11:1503879. doi: 10.3389/fnut.2024.1503879 (PMC11794082; doi:10.3389/fnut.2024.1503879)
Supplement: Supplementary file 1 [file Table_1.DOCX]

Supplementary Material

# Supplementary methods and materials

**Methods and materials**

PCR amplification of the 16S rRNA gene was performed as follows: initial denaturation at 95℃ for 3 min, followed by 30 cycles of denaturation at 95℃ for 30s, annealing at 55℃ for 30s and extension at 72℃ for 45, and single extension at 72℃ for 10mins, and end at 10℃.

**Table S1 The Reaction system of PCR amplification (20μL)**

| **Component** | **Volume (or mass)** |
| --- | --- |
| 5 × TransStart FastPfu buffer | 4μL |
| dNTPs (2.5mM) | 2μL |
| orward primer (5μM) | 0.8μL |
| reverse primer (5μM) | 0.8μL |
| ransStart FastPfu DNA Polymerase | 0.4μL |
| Template DNA | 10ng |
| dd H2O | XμL |
| Total | 20 μL |

**16SrRNA data analysis process:**

The Alpha diversity index Chao 1 and Shannon index were calculated by Mothur software (http://www.mothur.org/wiki/Calculators,versionv1.30.2), and the difference of Alpha diversity between groups was analyzed by the Wilcoxon rank sum test. PCoA analysis (principal coordinate analysis) based on the Bray-Curtis distance algorithm was used to test the similarity of microbial community structure between samples, and the PERMANOVA nonparametric test was used to analyze whether the difference of microbial community structure between samples was significant. R software was utilized to draw a Community bar plot to visually present the dominant species and relative abundance of each dominant species at different taxonomic levels.

Linear discriminant analysis Effect Size ( <http://huttenhower.sph.harvard.edu/LEfSe>) (LDA>2, P<0.05) was used to determine the bacterial groups with significant differences in abundance from phylum to genus level among different groups. The Kruskal-Wallis rank sum test was applied to detect species with different abundance in gut microbial communities of different groups.

**Chromatographic conditions:**

Chromatographic column: ACQUITY UPLC HSS T3 (100 mm × 2.1 mm i.d., 1.8μm; waters, Milford, USA); the mobile phase A was 95 % water + 5 % acetonitrile (containing 0.1 % formic acid), the mobile phase B was 47.5 % acetonitrile + 47.5 %, isopropanol + 5 % water (containing 0.1 % formic acid), the injection volume was 3μL, and the column temperature was 40 °C.

**Mass spectrometry conditions:**

The samples were ionized by electrospray ionization, and the mass spectrometry signals were collected by positive and negative ion scanning modes, respectively. The mass range (m/z) was set at 70–1050 in full scan resolution mode. The sheath gas flow rate and Aux gas flow rate were 50rab and 13rab, the heating temperature was 425 ℃, Capillary temp was 325 ℃. Positive spray voltage 3500 V, negative spray voltage 3500 V， S-Lens RF Level50, Collision energy 20ev, 40ev, 60ev.Resolution7500 MS2 and 60000.

**LC-MS data analysis process:**

The raw data of LC-MS were imported into Progenesis QI (Waters Corporation, Milford, USA) for baseline filtering, peak identification, integration, retention time correction, peak alignment, and finally a data matrix of retention time, mass-to-charge ratio, and peak intensity was obtained. The identification score threshold was 80%. Samples were normalized by summing (dividing all metabolite levels in a sample by the total signal observed in that sample) to make each sample comparable to the other. Variables with relative standard deviation (RSD) > 30% in the QC samples were also removed and the data were converted to a log 10 scale to obtain a final data matrix for subsequent analysis. Then, the obtained compounds were identified by searching MS / MS spectra in the Human Metabolome Database (HMDB) (http://www.hmdb.ca/)) and METLIN (https://metlin.scripps.edu/). Then, the R package “ropls” (Version 1.6.2) was used to perform principal component analysis (PCA) and orthogonal least partial squares discriminant analysis (OPLS-DA), and 7-cycle interactive validation evaluating the stability of the model. The significant differential metabolites were selected based on the variable weight value (VIP) obtained by the OPLS-DA model and the student's t-test p-value, Metabolites with VIP> 1, p-value <0.05 and fold change ≤ 0.83 or ≥ 1.2 were identified as significantly different metabolites. Differential metabolites among two groups were mapped into their biochemical pathways through metabolic enrichment and pathway analysis based on the KEGG database (http://www. genome.jp/kegg/)

# Supplementary Figures and Tables


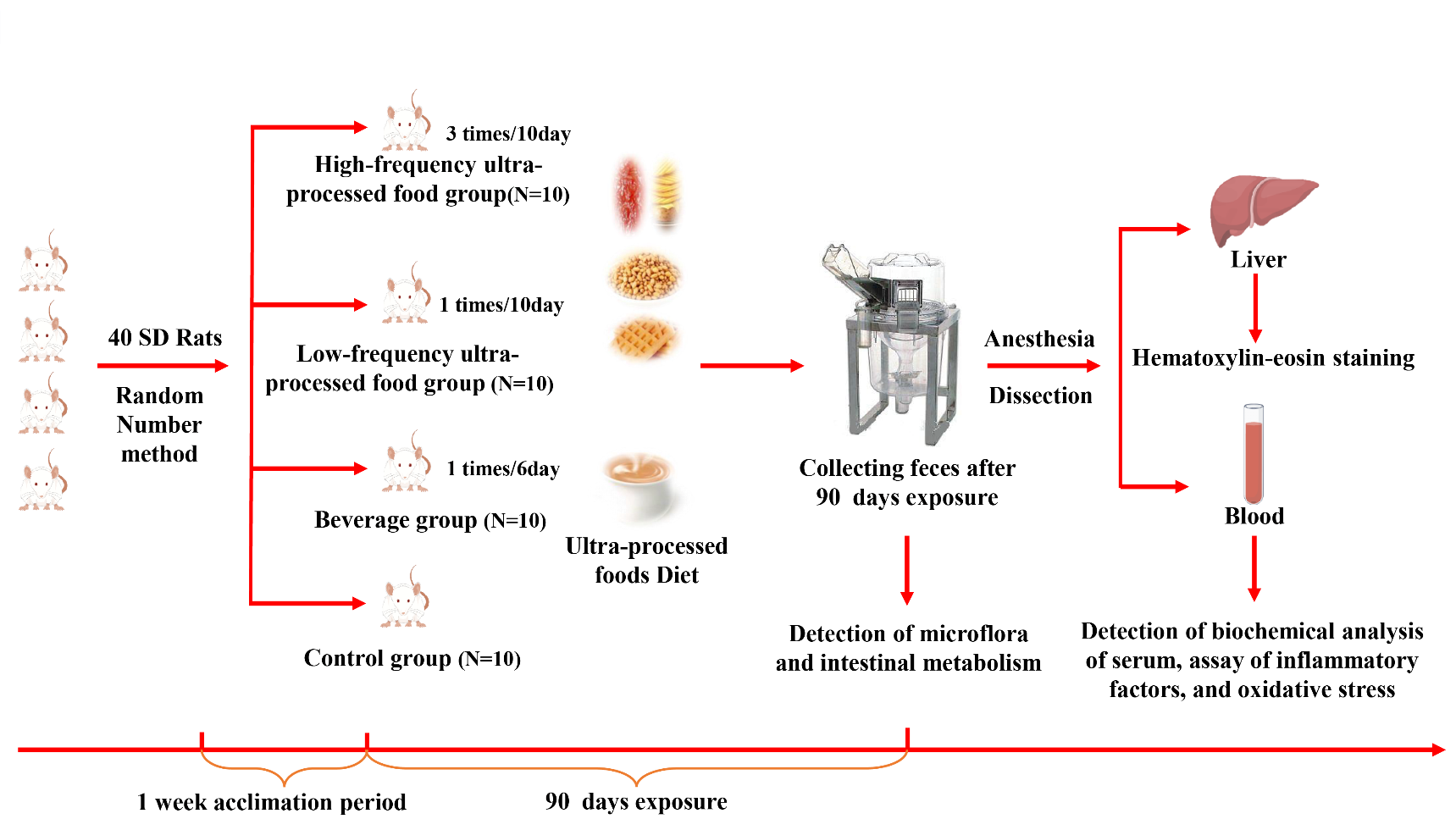


**Fig S1. The diagram of different feeding groups**

**
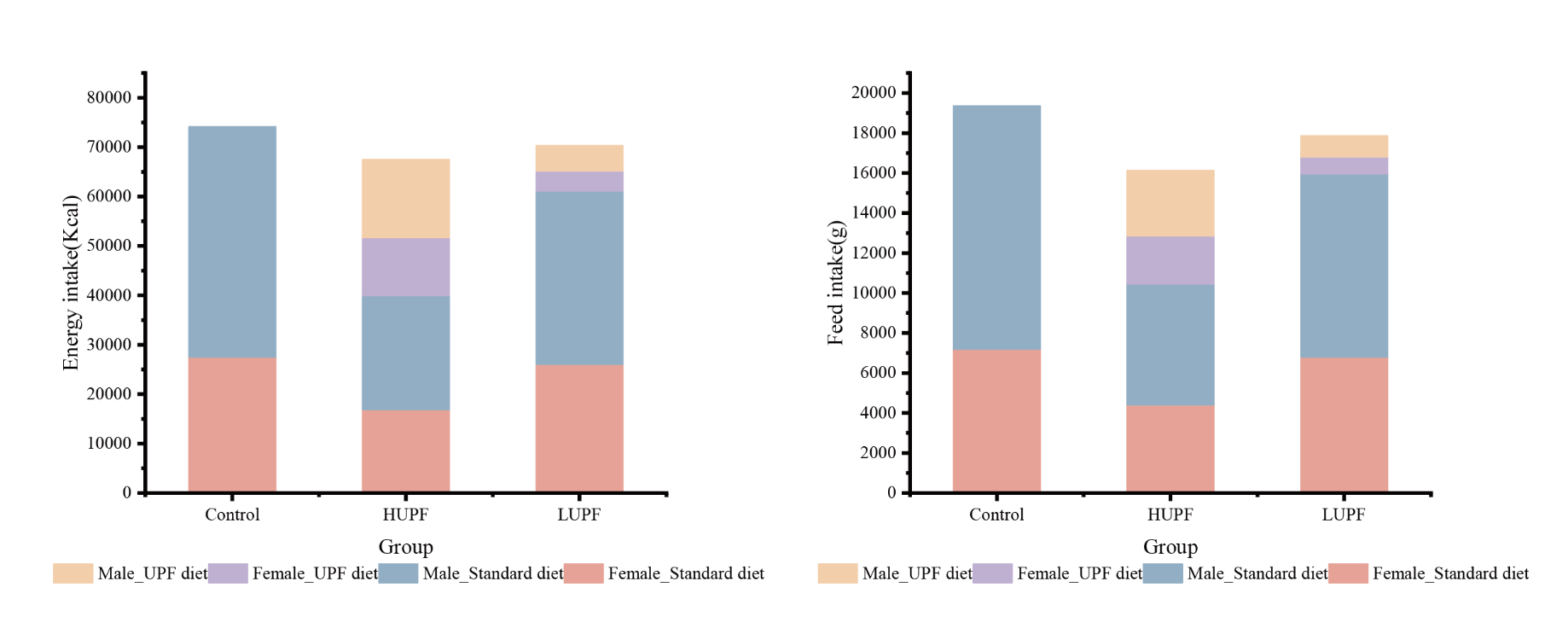
**

**Fig S2 Feed consumption and Energy intake (Day=90, N=10)**


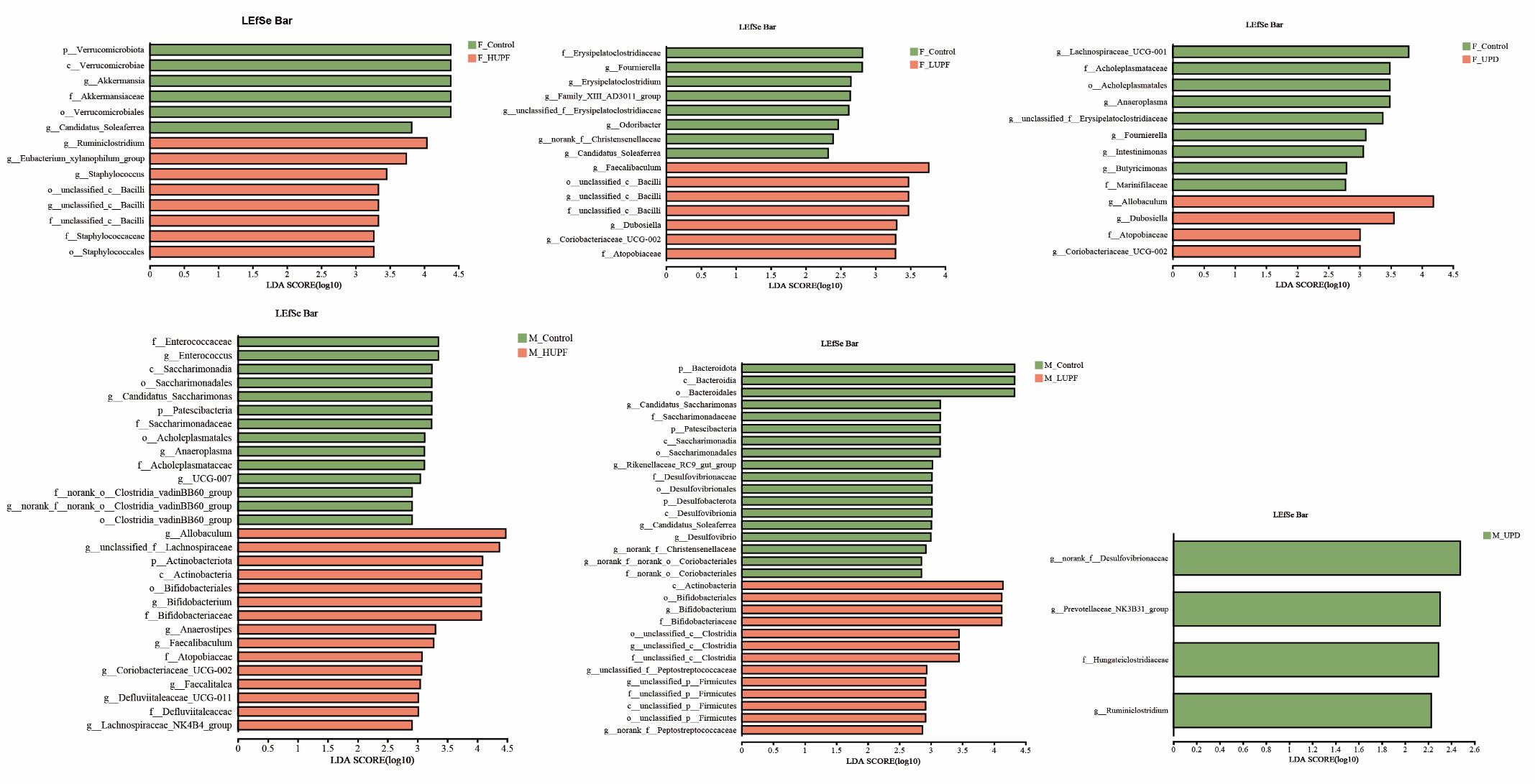


**Fig.S3 Cladogram visualizing the output of the LEfSe analysis. The most significant difference of gut microbial taxa among groups after LDA in female and male rats. The abbreviations are as follows: p, phylum; c, class; o, order; f, family; and g, genus.**

**Table S2 Alpha diversity index of gut microorganisms (Mean± SEM)**

|  | **Estimators** | **sobs** | **Chao** | **ace** | **Shannon** | **Simpson** | **Coverage** |
| --- | --- | --- | --- | --- | --- | --- | --- |
| At ASV level | F_HUPF | 1059.60±319.15 | 1059.60±319.15 | 1059.60±319.15 | 4.81±0.68 | 0.04±0.02 | 1±0 |
|  | F_LUPF | 1047.00±521.34 | 1047.00±521.34 | 1047.00±521.34 | 4.64±0.78 | 0.04±0.03 | 1±0 |
|  | F_UPD | 1020.20±291.66 | 1020.20±291.66 | 1020.20±291.66 | 4.89±0.36 | 0.02±0.01 | 1±0 |
|  | F_Control | 1182.80± 631.45 | 1182.80±631.45 | 1182.80± 631.45 | 4.89±0.63 | 0.03±0.02 | 1±0 |
|  | M_HUPF | 867.00±195.55 | 867.00±195.55 | 867.00±195.55 | 4.78±0.49 | 0.03±0.02 | 1±0 |
|  | M_LUPF | 1009.40±264.97 | 1009.40±264.97 | 1009.40±264.97 | 4.54±0.69 | 0.06±0.05 | 1±0 |
|  | M_UPD | 937.75±188.65 | 937.75±188.65 | 937.75±188.65 | 4.84±0.59 | 0.03±0.03 | 1±0 |
|  | M_Control | 785.40±210.00 | 785.40±210.00 | 785.40±210.00 | 4.62±0.46 | 0.04±0.02 | 1±0 |
|  | *P*_(F_HUPF_-_Fcontrol)_ | ≥ 0.1 | ≥ 0.1 | ≥ 0.1 | ≥ 0.1 | ≥ 0.1 | ≥ 0.1 |
|  | *P*_(F_LUPF-Fcontrol)_ | ≥ 0.1 | ≥ 0.1 | ≥ 0.1 | ≥ 0.1 | ≥ 0.1 | ≥ 0.1 |
|  | *P*_(F_UPD-Fcontrol)_ | ≥ 0.1 | ≥ 0.1 | ≥ 0.1 | ≥ 0.1 | ≥ 0.1 | ≥ 0.1 |
|  | *P*_(M_HUPF-Mcontrol)_ | ≥ 0.1 | ≥ 0.1 | ≥ 0.1 | ≥ 0.1 | ≥ 0.1 | ≥ 0.1 |
|  | *P*_(M_LUPF-Mcontrol)_ | ≥ 0.1 | ≥ 0.1 | ≥ 0.1 | ≥ 0.1 | ≥ 0.1 | ≥ 0.1 |
|  | *P*_(M_UPD-Mcontrol)_ | ≥ 0.1 | ≥ 0.1 | ≥ 0.1 | ≥ 0.1 | ≥ 0.1 | ≥ 0.1 |

**Table S3 Relative abundance of gut microbiota at the genus level**

| **Genus** | **Relative abundance (%)** | | | | | | | |
| --- | --- | --- | --- | --- | --- | --- | --- | --- |
|  | **F_HUPF** | **F_LUPF** | **F_UPD** | **F_Control** | **M_HUPF** | **M_LUPF** | **M_UPD** | **M_Control** |
| Lactobacillus | 14.19 | 17.71 | 12.26 | 8.67 | 10.22 | 19.81 | 7.36 | 10.53 |
| norank_f__Muribaculaceae | 10.86 | 10.56 | 13.07 | 8.26 | 10.24 | 6.82 | 10.18 | 9.57 |
| Turicibacter | 8.34 | 5.34 | 3.69 | 8.02 | 5.13 | 7.79 | 5.52 | 6.53 |
| norank_f__norank_o__Clostridia_UCG-014 | 10.75 | 6.41 | 11.15 | 8.0 | 10.58 | 14.10 | 20.93 | 13.49 |
| Clostridium_sensu_stricto_1 | 3.30 | 2.68 | 4.29 | 7.17 | 2.94 | 5.74 | <0.01 | 3.94 |
| unclassified_f__Lachnospiraceae | 4.44 | 6.03 | 8.09 | 6.37 | 7.34 | 4.54 | 3.44 | 2.95 |
| Escherichia-Shigella | 0.52 | 0.36 | 0.52 | 0.06 | 1.87 | 0.52 | 3.70 | 8.69 |
| Romboutsia | 6.97 | 6.27 | 5.26 | 5.73 | 5.77 | 8.07 | 3.89 | 7.76 |

| **Table S4 Screening results of differential metabolites** | | | | | | | |
| --- | --- | --- | --- | --- | --- | --- | --- |
| **Group** | **Metabolite** | **Mode** | **Formula** | **VIP** | **FC** | **P_value** | **Regulate** |
| F_LUPF | Cromakalim | pos | C16H18N2O3 | 5.4488 | 0.0971 | 7.67E-09 | down |
| F_LUPF | Momelotinib | neg | C23H22N6O2 | 5.2329 | 3.7155 | 1.61E-08 | up |
| F_LUPF | Paliperidone | pos | C23H27FN4O3 | 4.9669 | 3.3115 | 0.001267 | up |
| F_LUPF | Bursopoietin | pos | C14H25N7O3 | 4.9091 | 2.366 | 2.82E-06 | up |
| F_LUPF | Phendimetrazine | neg | C12H17NO | 4.8329 | 1469.8997 | 0.005004 | up |
| F_LUPF | 4-Methylumbelliferone sulfate | neg | C10H8O6S | 4.6534 | 2.7179 | 1.88E-07 | up |
| F_LUPF | Samidorphan | pos | C21H26N2O4 | 4.1404 | 1.9545 | 0.005839 | up |
| F_LUPF | Guanidine, N-((2S,3S,4R)-6-amino-2-(dimethoxymethyl)-3,4-dihydro-3-hydroxy-2-methyl-2H-1-benzopyran-4-yl)-N'-cyano-N''-(phenylmethyl)- | pos | C22H27N5O4 | 4.0698 | 1.5411 | 4.64E-11 | up |
| F_LUPF | 6-Deoxy-4-O-(3,6-di-O-methyl-beta-D-glucopyranosyl)-2,3-di-O-methyl-alpha-L-mannopyranose | pos | C16H30O10 | 3.9794 | 1.7663 | 0.0001587 | up |
| F_LUPF | Jangomolide | neg | C26H28O8 | 3.8913 | 2.066 | 0.03726 | up |
| F_LUPF | 2-Propenamide, 2-cyano-3-(4-hydroxy-3,5-bis(1-methylethyl)phenyl)- | pos | C16H20N2O2 | 3.8822 | 1.5979 | 1.28E-07 | up |
| F_LUPF | 5-Oxo-prolyl-glycyl-arginine-4-nitroanilide | neg | C19H26N8O6 | 3.7781 | 1.6191 | 4.87E-05 | up |
| F_LUPF | 1-(4-Aminophenyl)-7,8-dimethoxy-3,5-dihydro-2,3-benzodiazepin-4-one | neg | C17H17N3O3 | 3.7431 | 2.8728 | 0.01216 | up |
| F_LUPF | 3,5,6-Trihydroxy-5-(hydroxymethyl)-2-methoxy-2-cyclohexen-1-one | pos | C8H12O6 | 3.7322 | 2.3827 | 2.70E-06 | up |
| F_LUPF | 3-[5-(Dimethylcarbamoyl)pyrrolidin-3-yl]sulfanyl-6-(1-hydroxyethyl)-4-methyl-7-oxo-1-azabicyclo[3.2.0]hept-2-ene-2-carboxylic acid | pos | C17H25N3O5S | 3.6963 | 1.7012 | 0.003279 | up |
| F_LUPF | Cefroxadine | pos | C16H19N3O5S | 3.6893 | 1.5269 | 4.98E-11 | up |
| F_LUPF | Clofibryl glucuronide | neg | C16H19ClO9 | 3.6835 | 1.8224 | 0.0003144 | up |
| F_LUPF | Armillatin | pos | C38H58O6 | 3.6226 | 1.3679 | 2.09E-05 | up |
| F_LUPF | (R)-1-O-[b-D-Glucopyranosyl-(1->6)-b-D-glucopyranoside]-1,3-octanediol | pos | C20H38O12 | 3.6124 | 1.4661 | 2.98E-09 | up |
| F_LUPF | 2,6-Dimethoxy-1,4-benzoquinone | pos | C8H8O4 | 3.5529 | 1.9743 | 2.91E-06 | up |
| F_LUPF | Gly-Pro-Arg-Pro-Lys | pos | C24H43N9O6 | 3.5389 | 1.4513 | 1.14E-06 | up |
| F_LUPF | Ethyl (S)-3-hydroxybutyrate glucoside | pos | C12H22O8 | 3.5126 | 1.5109 | 9.08E-05 | up |
| F_LUPF | 15-Keto-prostaglandin E2 | pos | C20H30O5 | 3.5009 | 20.878 | 0.04091 | up |
| F_LUPF | 5-Sulfosalicylic acid | neg | C7H6O6S | 3.4803 | 1.6031 | 3.64E-07 | up |
| F_LUPF | Hetacillin | neg | C19H23N3O4S | 3.4795 | 0.5002 | 0.02474 | down |
| F_LUPF | Phenobarbital | pos | C12H12N2O3 | 3.4615 | 1.4293 | 1.14E-05 | up |
| F_LUPF | 3beta-hydroxy-4beta-methyl-5alpha-cholest-7-ene-4alpha-carboxylate | pos | C29H47O3- | 3.4415 | 1.6803 | 0.00981 | up |
| F_LUPF | Dodeca-2E,4E-Dienoic Acid Isobutylamide | pos | C16H29NO | 3.4209 | 0.5541 | 0.01483 | down |
| F_LUPF | Delavirdine | pos | C22H28N6O3S | 3.4172 | 1.4356 | 1.15E-05 | up |
| F_LUPF | Dihydro-beta-erythroidine | pos | C16H21NO3 | 3.4044 | 1.4336 | 0.0001597 | up |
| F_LUPF | 3-Carboxy-4-methyl-5-propyl-2-furanpropionic acid | pos | C12H16O5 | 3.3936 | 1.477 | 0.0003916 | up |
| F_LUPF | Netilmicin | pos | C21H41N5O7 | 3.3392 | 1.4734 | 3.52E-06 | up |
| F_LUPF | 4-Methylthiobenzamide-S-oxide | neg | C8H9NOS | 3.3329 | 1.5436 | 0.0001339 | up |
| F_LUPF | 8,9-Epoxyeicosatrienoic acid | pos | C20H32O3 | 3.3265 | 0.4751 | 0.03013 | down |
| F_LUPF | Ethyl 2-furanyl diketone | pos | C8H8O3 | 3.2343 | 1.355 | 5.95E-06 | up |
| F_LUPF | Loganate | pos | C16H23O10- | 3.2132 | 1.4305 | 2.76E-06 | up |
| F_LUPF | 25-Acetyl-6,7-didehydrofevicordin F 3-[glucosyl-(1->6)-glucoside] | neg | C43H62O18 | 3.1788 | 1.3383 | 0.0001403 | up |
| F_LUPF | Leucopelargonidin | pos | C15H14O6 | 3.1773 | 2.1287 | 0.02089 | up |
| F_LUPF | (2S)-2-Cyclopentyl-2-[4-[(2,4-dimethylpyrido[2,3-b]indol-9-yl)methyl]phenyl]-N-[(1S)-2-hydroxy-1-phenylethyl]acetamide | pos | C35H37N3O2 | 3.1678 | 1.3662 | 4.79E-08 | up |
| F_LUPF | Gamma-Aminobutyric acid glutamate | neg | C9H16N2O5 | 3.1507 | 1.4371 | 0.0007876 | up |
| F_LUPF | Dide-O-methylsimmondsin | pos | C14H21NO9 | 3.1451 | 1.5717 | 0.01749 | up |
| F_LUPF | (3s)-3-(Benzyloxy)-L-Aspartic Acid | pos | C11H13NO5 | 3.1174 | 1.4465 | 0.001529 | up |
| F_LUPF | Gamma-Glutamylcysteinylserine | pos | C11H19N3O7S | 3.1022 | 1.5578 | 0.005184 | up |
| F_LUPF | Hexahydro-6,7-dihydroxy-5-(hydroxymethyl)-3-(2-hydroxyphenyl)-2H-pyrano[2,3-d]oxazol-2-one | pos | C13H15NO7 | 3.0867 | 1.4501 | 0.006341 | up |
| F_LUPF | Baohuoside I | neg | C27H30O10 | 3.0862 | 1.5384 | 0.02413 | up |
| F_LUPF | Sparfloxacin | pos | C19H22F2N4O3 | 3.0648 | 1.2482 | 6.74E-06 | up |
| F_LUPF | Amikacin | pos | C22H43N5O13 | 3.0313 | 1.3017 | 4.19E-08 | up |
| F_LUPF | Fumitremorgin B | pos | C27H33N3O5 | 3.0181 | 1.4783 | 0.04115 | up |
| F_LUPF | Sphingosine | pos | C18H37NO2 | 3.0181 | 0.6706 | 1.08E-10 | down |
| F_LUPF | M-Coumaric acid | neg | C9H8O3 | 3.0163 | 1.5352 | 0.01016 | up |
| F_LUPF | Chenodeoxycholyltyrosine | neg | C33H49NO6 | 2.9892 | 1.4103 | 0.04331 | up |
| F_LUPF | 4-N-Methyllyaloside | pos | C28H33N2O9+ | 2.9793 | 2.2097 | 0.04912 | up |
| F_LUPF | Biliverdin | neg | C33H34N4O6 | 2.956 | 2.1645 | 0.03925 | up |
| F_LUPF | Arginyl-glycyl-glutamyl-serine | pos | C16H29N7O8 | 2.9471 | 0.7264 | 0.00444 | down |
| F_LUPF | S-Adenosylmethionine | pos | C15H23N6O5S+ | 2.9457 | 1.3312 | 0.001611 | up |
| F_LUPF | 4-(Glutamylamino) butanoate | neg | C9H16N2O5 | 2.9343 | 1.4022 | 0.001897 | up |
| F_LUPF | Clitocine | pos | C9H13N5O6 | 2.8899 | 1.5962 | 0.02746 | up |
| F_LUPF | Spirorenone | neg | C24H28O3 | 2.8846 | 1.2822 | 4.23E-05 | up |
| F_LUPF | Bakers yeast extract | pos | C19H14O2 | 2.8411 | 1.336 | 6.17E-06 | up |
| F_LUPF | Pyridin-4-ylmethyldiazene | pos | C6H7N3 | 2.8403 | 0.7177 | 0.01378 | down |
| F_LUPF | 1H-Pyrrole-3-carboxamide, 5-((5-fluoro-1,2-dihydro-2-oxo-3H-indol-3-ylidene)methyl)-N-((2S)-2-hydroxy-3-(4-morpholinyl)propyl)-2,4-dimethyl- | pos | C23H27FN4O4 | 2.8206 | 1.2144 | 9.55E-05 | up |
| F_LUPF | 1-(4-ethoxyphenyl)-3-[2-(1-ethylindol-3-yl)-2-pyridin-3-ylethyl]urea | pos | C26H28N4O2 | 2.8115 | 1.2061 | 0.0001027 | up |
| F_LUPF | (2R,4S)-4-Carbamimidamido-3-acetamido-2-((1R,2R)-2-hydroxy-1-methoxy-3-(octanoyloxy)propyl)-3,4-dihydro-2H-pyran-6-carboxylic acid | pos | C21H36N4O8 | 2.8045 | 1.2133 | 0.0002102 | up |
| F_LUPF | Gravacridonol | pos | C19H17NO4 | 2.7767 | 1.2998 | 0.0003303 | up |
| F_LUPF | Uridine diphosphate-N-acetylglucosamine | neg | C17H27N3O17P2 | 2.758 | 1.3038 | 0.01145 | up |
| F_LUPF | Phenmetrazine | neg | C11H15NO | 2.7573 | 1.3202 | 0.001387 | up |
| F_LUPF | Pregnenolone sulfate | neg | C21H32O5S | 2.751 | 1.2834 | 0.0001878 | up |
| F_LUPF | S-Allylcysteine | pos | C6H11NO2S | 2.7428 | 0.7522 | 0.006079 | down |
| F_LUPF | 2-[1-[(2S)-2-[[4-[(E)-N'-Hydroxycarbamimidoyl]benzoyl]amino]propanoyl]piperidin-4-yl]oxyacetic acid | neg | C18H24N4O6 | 2.7302 | 1.2326 | 9.03E-05 | up |
| F_LUPF | (R)-2,3-Dihydro-3,5-dihydroxy-2-oxo-3-indoleacetic acid | neg | C10H9NO5 | 2.6925 | 1.3653 | 0.01117 | up |
| F_LUPF | DG(2:0/20:3(8Z,11Z,14Z)-2OH(5,6)/0:0) | pos | C25H42O7 | 2.6618 | 1.2124 | 0.0001531 | up |
| F_LUPF | N-(N-L-gamma-Glutamyl-S-nitroso-L-cysteinyl)glycine | neg | C10H16N4O7S | 2.6581 | 1.4338 | 0.01812 | up |
| F_LUPF | Policapram | pos | C6H13NO | 2.6564 | 1.3757 | 0.008901 | up |
| F_LUPF | Strigol | pos | C19H22O6 | 2.6279 | 1.2673 | 0.00144 | up |
| F_LUPF | 7-[(1R,2R,3R,5S)-3,5-Dihydroxy-2-[(3R)-3-hydroxy-5-phenylpentyl]cyclopentyl]-5-heptenoic acid propan-2-yl ester | neg | C26H40O5 | 2.6211 | 0.8266 | 0.009027 | down |
| F_LUPF | Edetic Acid | neg | C10H16N2O8 | 2.6175 | 0.6333 | 0.04641 | down |
| F_LUPF | Limonoate a-ring-lactone | pos | C26H32O9 | 2.6054 | 0.7964 | 0.0002526 | down |
| F_LUPF | Histidinal | pos | C6H9N3O | 2.5528 | 0.8063 | 0.00227 | down |
| F_LUPF | Antramycin | pos | C16H17N3O4 | 2.5434 | 1.227 | 0.0001342 | up |
| F_LUPF | (1R,2R,3S,5S)-3-Hydroxy-4-(4-hydroxybenzoyl)-8-methyl-8-azabicyclo[3.2.1]octane-2-carboxylic acid | neg | C16H19NO5 | 2.5357 | 1.3037 | 0.0406 | up |
| F_LUPF | 5,10-Pentadecadien-1-ol | pos | C15H28O | 2.5309 | 1.3481 | 0.01917 | up |
| F_LUPF | Cyclo(Arg-Gly-Asp-D-Phe-Val) | neg | C26H38N8O7 | 2.5238 | 1.2053 | 0.0002663 | up |
| F_LUPF | Besifloxacin | pos | C19H21ClFN3O3 | 2.519 | 1.3466 | 0.04196 | up |
| F_LUPF | Thiamine | pos | C12H17N4OS+ | 2.5019 | 0.8272 | 0.009028 | down |
| F_LUPF | Melphalan | pos | C13H18Cl2N2O2 | 2.4844 | 0.7583 | 0.02514 | down |
| F_LUPF | Methylmalonic acid | pos | C4H6O4 | 2.4773 | 1.2988 | 0.04124 | up |
| F_LUPF | Methysticin | pos | C15H14O5 | 2.4765 | 1.2896 | 0.01118 | up |
| F_LUPF | 4-(3,4-Dihydroxyphenyl)-2,3-dihydro-2,3-dihydroxy-1H-phenalen-1-one | neg | C19H14O5 | 2.4764 | 1.2501 | 0.0004226 | up |
| F_LUPF | Isomaltoside | neg | C14H26O10 | 2.4721 | 1.2025 | 0.0001144 | up |
| F_LUPF | 4-amino-4-deoxychorismate | pos | C10H11NO5 | 2.4715 | 1.2613 | 0.009453 | up |
| F_LUPF | 4-Hydroxy-3,6,9-Trimethyl-3,3a,4,5,9a,9b-hexahydroazuleno[4,5-b]furan-2,7-Dione | neg | C15H18O4 | 2.4673 | 0.8036 | 0.002117 | down |
| F_LUPF | Deoxycholyltyrosine | pos | C33H49NO6 | 2.4422 | 1.2108 | 0.009259 | up |
| F_LUPF | 2-Aminoethyl hydrogen sulfate | neg | C2H7NO4S | 2.4315 | 1.3134 | 7.25E-05 | up |
| F_LUPF | Melleolide H | pos | C24H30O7 | 2.4128 | 1.2385 | 0.01374 | up |
| F_LUPF | Nalmefene | pos | C21H25NO3 | 2.4115 | 0.815 | 1.25E-05 | down |
| F_LUPF | Rutacultin | pos | C16H18O4 | 2.3875 | 0.7688 | 0.009274 | down |
| F_LUPF | Epinephrine | pos | C9H13NO3 | 2.3831 | 1.2782 | 0.01038 | up |
| F_LUPF | Coformycin | neg | C11H16N4O5 | 2.3697 | 1.2478 | 0.00243 | up |
| F_LUPF | P-Methylhippuric acid | pos | C10H11NO3 | 2.3633 | 1.3646 | 0.003848 | up |
| F_LUPF | 4-Oxo-1,4-dihydroquinoline-3-carboxylic acid | neg | C10H7NO3 | 2.3353 | 0.7968 | 0.00357 | down |
| F_LUPF | Hydroxyprolyl-Proline | neg | C10H16N2O4 | 2.3336 | 0.7651 | 0.01086 | down |
| F_LUPF | N-[(2R)-4-(Hydroxymethyl)-3-oxo-1-sulfanylhex-4-en-2-yl]acetamide | neg | C9H15NO3S | 2.3195 | 0.7708 | 0.03913 | down |
| F_LUPF | DG(20:4(5Z,8Z,11Z,14Z)-OH(17)/i-13:0/0:0) | neg | C36H62O6 | 2.3138 | 0.8267 | 0.02661 | down |
| F_LUPF | Creatinine oxalate | neg | C6H5N3O5 | 2.3003 | 1.2433 | 0.03295 | up |
| F_LUPF | Aminodeoxykanamycin | pos | C18H37N5O10 | 2.2876 | 0.8245 | 0.02596 | down |
| F_LUPF | 1-[2,3-Dimethyl-2-(2-methylbut-3-en-2-yl)furan-3-yl]-3,5-dihydroxy-4-methoxyhexan-2-one | neg | C18H30O5 | 2.2755 | 0.709 | 0.02795 | down |
| F_LUPF | Deoxycholic acid 3-glucuronide | pos | C30H48O10 | 2.2684 | 0.7995 | 0.01674 | down |
| F_LUPF | Dantron | pos | C14H8O4 | 2.2455 | 1.2559 | 0.02542 | up |
| F_LUPF | DG(14:0/18:1(9Z)-O(12,13)/0:0) | neg | C35H64O6 | 2.2261 | 0.827 | 0.04592 | down |
| F_LUPF | Coniferaldehyde | pos | C10H10O3 | 2.2128 | 1.226 | 0.01788 | up |
| F_LUPF | M-Cresol | neg | C7H8O | 2.1839 | 1.2375 | 0.0004561 | up |
| F_LUPF | 7-Hydroxyefavirenz | neg | C14H9ClF3NO3 | 2.1381 | 1.2433 | 0.02838 | up |
| F_LUPF | (S)-3-Sulfonatolactate | neg | C3H6O6S | 2.1367 | 1.2425 | 0.04892 | up |
| F_LUPF | (Z)-Resveratrol 3-(4''-sulfoglucoside) | neg | C20H22O11S | 2.1225 | 1.2085 | 0.03918 | up |
| F_LUPF | Threonylisoleucine | neg | C10H20N2O4 | 2.0961 | 0.8286 | 0.03853 | down |
| F_LUPF | Valine-betaxanthin | pos | C14H18N2O6 | 2.0942 | 1.2279 | 0.03939 | up |
| F_LUPF | Lamivudine | pos | C8H11N3O3S | 2.0678 | 1.206 | 0.008809 | up |
| F_LUPF | 5-methyl-3-phenyl-N-propan-2-yl-4H-1,2-oxazole-5-carboxamide | pos | C14H18N2O2 | 2.0559 | 0.8145 | 0.02338 | down |
| F_LUPF | Laninamivir | pos | C13H22N4O7 | 2.0422 | 1.2489 | 0.04417 | up |
| F_LUPF | Merodesmosine | pos | C18H34N4O6 | 2.0365 | 0.7706 | 0.03098 | down |
| F_LUPF | Prostaglandin G2 | neg | C20H32O6 | 2.0203 | 0.802 | 0.03504 | down |
| F_LUPF | Leu-Pro-Ile | pos | C17H31N3O4 | 2.0107 | 1.2025 | 0.04326 | up |
| F_LUPF | Furylacryloylalanyllysine | pos | C16H23N3O5 | 1.9979 | 0.7995 | 0.02889 | down |
| F_LUPF | 1-Tert-Butyl 4-ethyl 3-oxopiperidine-1,4-dicarboxylate | neg | C13H21NO5 | 1.9541 | 1.2067 | 0.04889 | up |
| F_LUPF | N-acetyl-S-(3-oxo-3-carboxy-n-propyl)cysteine | pos | C9H13NO6S | 1.9424 | 1.2299 | 0.03995 | up |
| F_LUPF | Maleylacetoacetic acid | pos | C8H8O6 | 1.9299 | 1.2177 | 0.01844 | up |
| F_LUPF | Isopentenyl pyrophosphate | neg | C5H12O7P2 | 1.9145 | 1.2558 | 0.0363 | up |
| F_LUPF | 2-(1,2,3,4-Tetrahydroxybutyl)thiazolidine-4-carboxylic acid | pos | C8H15NO6S | 1.8704 | 1.2097 | 0.0377 | up |
| F_LUPF | Thr Phe | pos | C13H18N2O4 | 1.8679 | 1.2104 | 0.01919 | up |
| F_LUPF | Benzeneacetic acid, 4-(2-(diethylamino)-2-oxoethoxy)-3-ethoxy-, propyl ester | neg | C19H29NO5 | 1.842 | 0.7429 | 0.04889 | down |
| F_LUPF | Propofol glucuronide | neg | C18H26O7 | 1.7596 | 0.8288 | 0.008095 | down |
| F_HUPF | Phendimetrazine | neg | C12H17NO | 6.3627 | 2508.7793 | 1.74E-09 | up |
| F_HUPF | Pisatoside | pos | C10H15NO7 | 6.2576 | 14.4302 | 2.47E-06 | up |
| F_HUPF | 4-N-Methyllyaloside | pos | C28H33N2O9+ | 5.6908 | 3.6901 | 7.94E-08 | up |
| F_HUPF | (S)-[8]-Gingerol | neg | C19H30O4 | 5.2826 | 6.6704 | 1.44E-06 | up |
| F_HUPF | 1-(4-Aminophenyl)-7,8-dimethoxy-3,5-dihydro-2,3-benzodiazepin-4-one | neg | C17H17N3O3 | 5.2219 | 3.9383 | 0.0006731 | up |
| F_HUPF | 3-Carboxy-4-methyl-5-propyl-2-furanpropionic acid | pos | C12H16O5 | 5.1784 | 1.995 | 3.45E-06 | up |
| F_HUPF | Gamma-Glutamylcysteinylserine | pos | C11H19N3O7S | 5.1508 | 2.2283 | 1.06E-05 | up |
| F_HUPF | 2,6-Dimethoxy-1,4-benzoquinone | pos | C8H8O4 | 5.1119 | 2.9542 | 5.30E-06 | up |
| F_HUPF | 24(28)-Dehydromakisterone | pos | C28H44O7 | 5.102 | 3.0807 | 2.32E-05 | up |
| F_HUPF | Hexahydro-6,7-dihydroxy-5-(hydroxymethyl)-3-(2-hydroxyphenyl)-2H-pyrano[2,3-d]oxazol-2-one | pos | C13H15NO7 | 5.0265 | 1.9943 | 6.36E-05 | up |
| F_HUPF | Silidianin | neg | C25H24O10 | 5.0088 | 3.8206 | 6.35E-07 | up |
| F_HUPF | (3s)-3-(Benzyloxy)-L-Aspartic Acid | pos | C11H13NO5 | 4.9725 | 1.9857 | 9.39E-06 | up |
| F_HUPF | Loganoside | pos | C17H26O10 | 4.9723 | 3.0136 | 2.66E-05 | up |
| F_HUPF | Sar-Pro-Arg-pNA | neg | C20H30N8O5 | 4.9079 | 3.7378 | 2.41E-06 | up |
| F_HUPF | Ptelatoside A | neg | C19H26O10 | 4.8229 | 4.1241 | 4.27E-05 | up |
| F_HUPF | Cephalexin | pos | C16H17N3O4S | 4.7978 | 2.2667 | 2.54E-11 | up |
| F_HUPF | 3-(4-Carboxybenzylidene)-6-hydroxycamphor | pos | C18H20O4 | 4.7862 | 3.0191 | 1.96E-05 | up |
| F_HUPF | 2'-Deoxymugineic acid | neg | C12H20N2O7 | 4.7729 | 3.5114 | 6.86E-06 | up |
| F_HUPF | 4-Methylumbelliferone sulfate | neg | C10H8O6S | 4.7363 | 3.0614 | 1.13E-11 | up |
| F_HUPF | Methysticin | pos | C15H14O5 | 4.7266 | 1.8026 | 1.13E-05 | up |
| F_HUPF | 15-Keto-prostaglandin E2 | pos | C20H30O5 | 4.6885 | 29.9076 | 0.005247 | up |
| F_HUPF | 3,5,6-Trihydroxy-5-(hydroxymethyl)-2-methoxy-2-cyclohexen-1-one | pos | C8H12O6 | 4.6285 | 3.0304 | 7.16E-06 | up |
| F_HUPF | Bursopoietin | pos | C14H25N7O3 | 4.4932 | 2.0954 | 2.81E-05 | up |
| F_HUPF | Gamma-glutamyl-L-putrescine | pos | C9H19N3O3 | 4.4275 | 2.0227 | 0.0008986 | up |
| F_HUPF | Clofibryl glucuronide | neg | C16H19ClO9 | 4.4122 | 2.3215 | 4.71E-09 | up |
| F_HUPF | Ethyl 2-hydroxy-3-(3-indolyl)propanoate glucoside | pos | C19H25NO8 | 4.4047 | 2.7822 | 7.64E-05 | up |
| F_HUPF | 4-amino-4-deoxychorismate | pos | C10H11NO5 | 4.4035 | 1.664 | 8.54E-05 | up |
| F_HUPF | Epinephrine | pos | C9H13NO3 | 4.3947 | 1.734 | 6.94E-05 | up |
| F_HUPF | Glycerol 1-(5-hydroxydodecanoate) | pos | C15H30O5 | 4.3796 | 2.3234 | 0.00173 | up |
| F_HUPF | 4-Methylthiobenzamide-S-oxide | neg | C8H9NOS | 4.3536 | 1.9945 | 2.67E-07 | up |
| F_HUPF | (-)-Wikstromol | pos | C20H22O7 | 4.3163 | 4.3899 | 0.007726 | up |
| F_HUPF | Laninamivir | pos | C13H22N4O7 | 4.2226 | 1.7254 | 0.0003176 | up |
| F_HUPF | Advantame | pos | C24H30N2O7 | 4.1976 | 2.1356 | 0.002341 | up |
| F_HUPF | Acetyldeoxynivalenol | neg | C17H22O7 | 4.1787 | 2.3823 | 0.0007214 | up |
| F_HUPF | Phenmetrazine | neg | C11H15NO | 4.167 | 1.8017 | 1.47E-06 | up |
| F_HUPF | Histidylglutamine | neg | C11H17N5O4 | 4.1475 | 2.1342 | 1.87E-05 | up |
| F_HUPF | Cimifugin | pos | C16H18O6 | 4.1474 | 1.9891 | 0.004068 | up |
| F_HUPF | Gynocardin | neg | C12H17NO8 | 4.1467 | 1.9033 | 6.42E-05 | up |
| F_HUPF | (3E)-Glutaconylcarnitin | pos | C12H19NO6 | 4.1096 | 1.6691 | 4.67E-05 | up |
| F_HUPF | Aloesol 7-glucoside | neg | C19H24O9 | 4.1079 | 2.5599 | 0.0002918 | up |
| F_HUPF | Maleylacetoacetic acid | pos | C8H8O6 | 4.0976 | 1.7443 | 4.92E-05 | up |
| F_HUPF | 2-(1,2,3,4-Tetrahydroxybutyl)thiazolidine-4-carboxylic acid | pos | C8H15NO6S | 4.0799 | 1.6762 | 1.89E-05 | up |
| F_HUPF | 10-Methyl-9-(10-methyl-1H-acridin-9-yl)-1H-acridine | neg | C28H24N2 | 4.036 | 2.0474 | 0.000269 | up |
| F_HUPF | 6,7-Dimethyl-8-(1-D-ribityl)lumazine | pos | C13H18N4O6 | 3.9787 | 1.6608 | 0.0006442 | up |
| F_HUPF | Indacrinone | neg | C18H14Cl2O4 | 3.9747 | 1.7622 | 1.04E-07 | up |
| F_HUPF | Ethyl 2-furanyl diketone | pos | C8H8O3 | 3.965 | 1.5015 | 9.23E-09 | up |
| F_HUPF | N-Methyltyramine | pos | C9H13NO | 3.9647 | 3.1842 | 0.003879 | up |
| F_HUPF | 5-Sulfosalicylic acid | neg | C7H6O6S | 3.9401 | 1.8687 | 1.41E-08 | up |
| F_HUPF | Cromakalim | pos | C16H18N2O3 | 3.9267 | 0.3906 | 0.02079 | down |
| F_HUPF | Prostaglandin M | pos | C16H24O7 | 3.8738 | 1.6993 | 0.001878 | up |
| F_HUPF | 6-Acetylmorphine | pos | C19H21NO4 | 3.868 | 2.1709 | 0.005575 | up |
| F_HUPF | 5-Acetyl-3,4-dihydro-2H-pyrrole | neg | C6H9NO | 3.8616 | 2.0105 | 5.00E-07 | up |
| F_HUPF | BL IV | neg | C24H18O10 | 3.8608 | 5.2632 | 0.01137 | up |
| F_HUPF | Glutaminylisoleucine | neg | C11H21N3O4 | 3.7855 | 2.304 | 0.01238 | up |
| F_HUPF | N-Benzoylaspartic acid | neg | C11H11NO5 | 3.7631 | 1.8268 | 0.0001669 | up |
| F_HUPF | 2-Fluoro-2',3'-dideoxyadenosine | neg | C10H12FN5O2 | 3.7587 | 1.6186 | 8.46E-05 | up |
| F_HUPF | Melibiitol | pos | C12H24O11 | 3.7475 | 1.5227 | 0.0003778 | up |
| F_HUPF | Alginic acid | neg | C12H16O12P2 | 3.7409 | 1.7313 | 2.66E-05 | up |
| F_HUPF | FA 15 (antioxidant) | pos | C31H49NO2 | 3.7293 | 1.5766 | 0.0183 | up |
| F_HUPF | Bendiocarb | neg | C11H13NO4 | 3.7098 | 1.6066 | 0.0002367 | up |
| F_HUPF | Tryptophyl-Glutamine | neg | C16H20N4O4 | 3.6858 | 1.6868 | 0.002811 | up |
| F_HUPF | 11,14,15-THETA | pos | C20H34O5 | 3.6717 | 1.6531 | 0.0007696 | up |
| F_HUPF | N-Acetyl-L-Histidine | pos | C8H11N3O3 | 3.6061 | 2.2507 | 0.03784 | up |
| F_HUPF | Pyridoxal 5'-phosphate | pos | C8H10NO6P | 3.5912 | 1.5202 | 0.0007124 | up |
| F_HUPF | Quercetin 3-O-sophoroside | pos | C14H16N2O6 | 3.5687 | 1.8728 | 0.008396 | up |
| F_HUPF | Thermophillin | pos | C8H8O4 | 3.5489 | 1.8861 | 0.004255 | up |
| F_HUPF | 1-Tert-Butyl 4-ethyl 3-oxopiperidine-1,4-dicarboxylate | neg | C13H21NO5 | 3.5359 | 1.5727 | 2.07E-05 | up |
| F_HUPF | 24-Methylenelophenol | pos | C29H48O | 3.5159 | 1.561 | 0.005872 | up |
| F_HUPF | Todatriol glucoside | pos | C17H26O10 | 3.4989 | 1.545 | 0.007348 | up |
| F_HUPF | 1-[(4-Amino-3-methylphenyl)methyl]-5-(2,2-diphenylacetyl)-6,7-dihydro-4H-imidazo[4,5-c]pyridine-6-carboxylic acid | pos | C29H28N4O3 | 3.4984 | 1.7747 | 0.008567 | up |
| F_HUPF | L-Aspoxicillin trihydrate | neg | C21H27N5O7S | 3.4941 | 1.5657 | 1.19E-05 | up |
| F_HUPF | (1R,2R,3S,5S)-3-Hydroxy-4-(4-hydroxybenzoyl)-8-methyl-8-azabicyclo[3.2.1]octane-2-carboxylic acid | neg | C16H19NO5 | 3.4625 | 1.4544 | 0.006863 | up |
| F_HUPF | (8R,9R,10S,13S,17R)-11,17-Dihydroxy-17-(2-hydroxyacetyl)-1,10,13-trimethyl-7,8,9,11,12,14,15,16-octahydro-6H-cyclopenta[a]phenanthren-3-one | pos | C22H30O5 | 3.45 | 1.5798 | 0.002987 | up |
| F_HUPF | Butabarbital | neg | C10H16N2O3 | 3.446 | 1.7518 | 0.0004252 | up |
| F_HUPF | 4beta-hydroxymethyl-4alpha-methyl-5alpha-cholest-7-en-3beta-ol | pos | C29H50O2 | 3.4439 | 1.3669 | 1.33E-05 | up |
| F_HUPF | (2S,3S,5S,8S,9S,10S,13S,14S,17S)-17-Acetyl-2-(2,2-dimethylmorpholino)-3-hydroxy-10,13-dimethylhexadecahydro-11H-cyclopenta[a]phenanthren-11-one | pos | C27H43NO4 | 3.4363 | 0.5097 | 0.02257 | down |
| F_HUPF | Tryptophyl-Arginine | pos | C17H24N6O3 | 3.4331 | 1.5227 | 0.006915 | up |
| F_HUPF | DG(14:1(9Z)/20:5(5Z,8Z,11Z,14Z,17Z)/0:0) | pos | C37H60O5 | 3.428 | 1.5446 | 0.007972 | up |
| F_HUPF | 3'-Hydroxystanozolol | neg | C21H32N2O2 | 3.4083 | 1.7644 | 0.001539 | up |
| F_HUPF | 1,5-Dimethyl citrate | pos | C8H12O7 | 3.4028 | 1.5084 | 2.52E-06 | up |
| F_HUPF | 3beta-hydroxy-4beta-methyl-5alpha-cholest-7-ene-4alpha-carboxylate | pos | C29H47O3- | 3.3829 | 1.6525 | 0.01214 | up |
| F_HUPF | N(6)-6(R,S)-lipoyl-L-lysine | neg | C14H26N2O3S2 | 3.3761 | 1.4951 | 0.0002114 | up |
| F_HUPF | 6-Acetyl-1,2,3,4-tetrahydropyridine | neg | C7H11NO | 3.3216 | 1.7408 | 0.0005811 | up |
| F_HUPF | Oxolinic acid | neg | C13H11NO5 | 3.3201 | 1.6054 | 2.43E-06 | up |
| F_HUPF | 2'-Deoxy-2'-methylenecytidine | pos | C10H13N3O4 | 3.3185 | 1.7551 | 0.0134 | up |
| F_HUPF | Proline betaine | pos | C7H13NO2 | 3.313 | 1.3145 | 0.0008424 | up |
| F_HUPF | 4-Hydroxy-L-phenylglycine | neg | C8H9NO3 | 3.3118 | 1.3831 | 2.58E-06 | up |
| F_HUPF | 1-(3-Pyridinyl)-1,4-butanediol | neg | C9H13NO2 | 3.3066 | 1.5543 | 0.0005513 | up |
| F_HUPF | Cholesterol glutamate | neg | C32H51NO5 | 3.2901 | 2.3457 | 0.006949 | up |
| F_HUPF | Benzamide, 4-chloro-N-(2-(3-oxo-4-morpholinyl)ethyl)- | neg | C13H15ClN2O3 | 3.2801 | 2.349 | 0.005668 | up |
| F_HUPF | N2-(1-Carboxyethyl)-2'-deoxyguanosine | pos | C13H17N5O6 | 3.2772 | 1.5552 | 0.002172 | up |
| F_HUPF | Phosphonol | pos | C6H17N2O3PS | 3.2669 | 1.562 | 0.0004737 | up |
| F_HUPF | L-DOPA 3'-glucoside | neg | C15H21NO9 | 3.2636 | 5.1999 | 0.03896 | up |
| F_HUPF | N-(3-Oxohexanoyl)homoserine Lactone | pos | C10H15NO4 | 3.2518 | 1.5352 | 0.002949 | up |
| F_HUPF | Gibberellin A86 | neg | C19H24O8 | 3.2464 | 1.4278 | 0.0002397 | up |
| F_HUPF | Prunasin | pos | C14H17NO6 | 3.2457 | 1.392 | 8.47E-06 | up |
| F_HUPF | 1-Hexacosanol | pos | C26H54O | 3.2386 | 1.3044 | 3.62E-05 | up |
| F_HUPF | Nona-3,5,7-trienedioylcarnitine | pos | C16H23NO6 | 3.2289 | 1.2906 | 0.002144 | up |
| F_HUPF | Indole-3-acetamide | neg | C10H10N2O | 3.2244 | 1.4561 | 0.0007216 | up |
| F_HUPF | Vanillic acid | pos | C8H8O4 | 3.2106 | 1.7563 | 0.005347 | up |
| F_HUPF | 5-Acetyl-2,3-dihydro-1H-pyrrolizine | neg | C9H11NO | 3.207 | 1.6851 | 0.0002155 | up |
| F_HUPF | N-methylethanolamine phosphate | neg | C3H9NO4P- | 3.1812 | 1.5605 | 0.001596 | up |
| F_HUPF | Resolvin D2 | pos | C22H32O5 | 3.1704 | 1.4128 | 0.003553 | up |
| F_HUPF | Avenic acid A | pos | C12H22N2O8 | 3.1476 | 1.6198 | 0.01028 | up |
| F_HUPF | (S)-(+)-1-(p-Hydroxy-trans-cinnamoyl)-glycerol | neg | C12H14O5 | 3.1464 | 1.758 | 0.02097 | up |
| F_HUPF | 2-Methylhippuric Acid | neg | C10H11NO3 | 3.1439 | 1.408 | 0.0001278 | up |
| F_HUPF | Thr Phe | pos | C13H18N2O4 | 3.1323 | 1.5387 | 0.009825 | up |
| F_HUPF | 10-Acetylpanaxytriol | pos | C19H28O4 | 3.1311 | 1.5156 | 0.006046 | up |
| F_HUPF | 2-Propionylpyrrole | neg | C7H9NO | 3.1291 | 1.5908 | 0.001856 | up |
| F_HUPF | Mycophenolic Acid | pos | C17H20O6 | 3.0797 | 1.4062 | 0.002586 | up |
| F_HUPF | M-Cresol | neg | C7H8O | 3.0782 | 1.4841 | 9.53E-05 | up |
| F_HUPF | Neotussilagine | neg | C10H17NO3 | 3.0761 | 2.0544 | 0.02527 | up |
| F_HUPF | 11-Hydroxy-9-tridecenoic acid | pos | C13H24O3 | 3.0246 | 1.3587 | 0.002236 | up |
| F_HUPF | Sphingosine | pos | C18H37NO2 | 3.0166 | 0.6817 | 9.18E-11 | down |
| F_HUPF | 3-O-Methyl-a-methyldopa | neg | C10H13NO4 | 3.0041 | 1.5245 | 7.68E-06 | up |
| F_HUPF | Z-Leu-Leu-Norvalinal | neg | C25H39N3O5 | 3.0026 | 0.6561 | 0.03122 | down |
| F_HUPF | Glu Tyr | pos | C14H18N2O6 | 2.996 | 1.4125 | 0.001883 | up |
| F_HUPF | 2-Aminoethyl hydrogen sulfate | neg | C2H7NO4S | 2.9752 | 1.6427 | 0.005619 | up |
| F_HUPF | Loline | pos | C8H14N2O | 2.9677 | 1.4336 | 0.003571 | up |
| F_HUPF | 5'-O-beta-D-Glucosylpyridoxine | pos | C14H21NO8 | 2.9604 | 1.37 | 0.00515 | up |
| F_HUPF | 6-Fluoropyridoxol | neg | C8H10FNO3 | 2.9499 | 1.4849 | 3.94E-07 | up |
| F_HUPF | Hyaluronan biosynthesis, precursor 1 | neg | C14H21NO11 | 2.9394 | 1.7972 | 0.04518 | up |
| F_HUPF | Moschamine | pos | C20H20N2O4 | 2.9377 | 1.6355 | 0.02173 | up |
| F_HUPF | 2,1,3-Benzoxadiazole-4-sulfonic acid, 7-fluoro- | neg | C6H3FN2O4S | 2.9236 | 1.4637 | 0.002471 | up |
| F_HUPF | Ethyl benzoate | pos | C9H10O2 | 2.9101 | 1.2838 | 0.004481 | up |
| F_HUPF | 11-Dehydro-thromboxane B2 | neg | C20H32O6 | 2.898 | 1.8839 | 0.008763 | up |
| F_HUPF | Ile-Val-OH | neg | C16H22N2O6 | 2.8925 | 1.43 | 0.001029 | up |
| F_HUPF | Tyrosyl-Proline | pos | C14H18N2O4 | 2.8716 | 1.4688 | 0.02216 | up |
| F_HUPF | Cysteinyl-Arginine | pos | C9H19N5O3S | 2.87 | 1.4453 | 0.01904 | up |
| F_HUPF | Quetiapine | neg | C21H25N3O2S | 2.8467 | 1.504 | 0.004973 | up |
| F_HUPF | 4-Hydroxy Duloxetine | pos | C18H19NO2S | 2.8465 | 1.3317 | 0.004166 | up |
| F_HUPF | Dimethylguanidino valeric acid | neg | C8H15N3O3 | 2.8253 | 1.5024 | 0.01647 | up |
| F_HUPF | 3,4-Methyleneadipic acid | neg | C8H10O4 | 2.8159 | 1.2879 | 1.53E-08 | up |
| F_HUPF | Cinncassiol C2 | pos | C20H28O6 | 2.7988 | 1.2984 | 0.006775 | up |
| F_HUPF | Niazirinin | pos | C16H19NO6 | 2.7875 | 1.2995 | 0.006011 | up |
| F_HUPF | (S)-3-Sulfonatolactate | neg | C3H6O6S | 2.7828 | 1.3196 | 0.01645 | up |
| F_HUPF | Telotristat | neg | C25H22ClF3N6O3 | 2.744 | 1.3456 | 0.002447 | up |
| F_HUPF | Dide-O-methylsimmondsin | pos | C14H21NO9 | 2.7311 | 1.439 | 0.03558 | up |
| F_HUPF | L-cis-Cyclo(aspartylphenylalanyl) | neg | C13H14N2O4 | 2.7251 | 1.3632 | 0.001602 | up |
| F_HUPF | 10-Piperazinylpropylphenothiazine | neg | C19H23N3S | 2.7182 | 1.6804 | 0.01703 | up |
| F_HUPF | Sesamol | neg | C7H6O3 | 2.7159 | 1.2598 | 5.09E-05 | up |
| F_HUPF | Tetraxetan | pos | C16H28N4O8 | 2.7155 | 1.2621 | 0.001994 | up |
| F_HUPF | Gamma-Glutamyldopa | pos | C14H18N2O7 | 2.7021 | 1.2287 | 0.002106 | up |
| F_HUPF | 16-Nitroxystearate | pos | C22H43NO4 | 2.6935 | 1.2843 | 0.001118 | up |
| F_HUPF | Humilixanthin | pos | C14H18N2O7 | 2.691 | 1.3236 | 0.007813 | up |
| F_HUPF | Lenticin | pos | C14H18N2O2 | 2.6893 | 0.7864 | 0.005305 | down |
| F_HUPF | Benzamide | neg | C7H7NO | 2.674 | 1.2691 | 3.21E-05 | up |
| F_HUPF | Gibberellin A5 | neg | C19H22O5 | 2.664 | 1.7675 | 0.01553 | up |
| F_HUPF | 2'-O-Methylcytidine | pos | C10H15N3O5 | 2.6608 | 1.2914 | 0.009443 | up |
| F_HUPF | 18-Hydroxycorticosterone | pos | C21H30O5 | 2.6545 | 1.3005 | 0.01041 | up |
| F_HUPF | Codeine | pos | C18H21NO3 | 2.6499 | 1.3195 | 0.002139 | up |
| F_HUPF | M-Trifluoromethylhippuric acid | neg | C10H8F3NO3 | 2.6434 | 1.6184 | 0.02196 | up |
| F_HUPF | 2,3-Dimethoxyphenol sulfate | neg | C8H10O6S | 2.6428 | 1.5004 | 0.01991 | up |
| F_HUPF | 17-phenyl-18,19,20-trinor-prostaglandin E2 | pos | C23H30O5 | 2.6202 | 1.3062 | 0.0145 | up |
| F_HUPF | 4-Mercaptobutyramidine | neg | C4H10N2S | 2.6164 | 1.3267 | 3.10E-06 | up |
| F_HUPF | D-4-Hydroxyphenylglycine | pos | C8H9NO3 | 2.6151 | 1.2493 | 0.002801 | up |
| F_HUPF | 1-[(2R,4R,5R)-3,4-Dihydroxy-5-(hydroxymethyl)-2-oxolanyl]-2-pyrimidinone | neg | C9H12N2O5 | 2.6077 | 1.5402 | 0.02954 | up |
| F_HUPF | 6-Dehydrotestosterone glucuronide | neg | C25H34O8 | 2.5946 | 1.3376 | 0.003143 | up |
| F_HUPF | Vanillylmandelic acid | neg | C9H10O5 | 2.5789 | 1.3056 | 0.02186 | up |
| F_HUPF | (S,E)-Zearalenone | neg | C18H22O5 | 2.5717 | 1.6311 | 0.03776 | up |
| F_HUPF | 17-HDoHE | pos | C22H32O3 | 2.5678 | 1.2412 | 0.01053 | up |
| F_HUPF | 11-Dehydrocorticosterone | pos | C21H28O4 | 2.5673 | 1.2504 | 0.005711 | up |
| F_HUPF | Terazosin | neg | C19H25N5O4 | 2.5612 | 1.2617 | 0.001385 | up |
| F_HUPF | Calystegine B2 | pos | C7H13NO4 | 2.5408 | 1.3961 | 0.03618 | up |
| F_HUPF | 16-B1-phytoprostane | pos | C18H28O4 | 2.5368 | 1.2302 | 0.007089 | up |
| F_HUPF | Coniferaldehyde | pos | C10H10O3 | 2.5275 | 1.2838 | 0.01001 | up |
| F_HUPF | 3-[(3-(2-Carboxyethyl)-4-methylpyrrol-2-YL)methylene]-2-indolinone | neg | C17H16N2O3 | 2.5246 | 1.2822 | 0.0001792 | up |
| F_HUPF | Boc-D-phenylalanine | pos | C14H19NO4 | 2.5244 | 1.2344 | 0.01173 | up |
| F_HUPF | Tetraphyllin B | pos | C12H17NO7 | 2.5182 | 1.3046 | 0.01376 | up |
| F_HUPF | 2,6-Diamino-9-(2-hydroxyethoxymethyl)purine | pos | C8H12N6O2 | 2.5121 | 1.2803 | 0.02704 | up |
| F_HUPF | 2H-1-Benzopyran-2-one, 7-[[2-(acetylamino)-2-deoxy-beta-D-glucopyranosyl]oxy]-4-methyl- | pos | C18H21NO8 | 2.5032 | 1.3557 | 0.04211 | up |
| F_HUPF | Melleolide H | pos | C24H30O7 | 2.4956 | 1.237 | 0.005564 | up |
| F_HUPF | Taps | neg | C7H17NO6S | 2.4867 | 1.2465 | 0.003285 | up |
| F_HUPF | 15-Keto-prostaglandin F2a | pos | C20H32O5 | 2.4853 | 1.3325 | 0.03536 | up |
| F_HUPF | Riboprine | pos | C15H21N5O4 | 2.4642 | 1.2306 | 0.00548 | up |
| F_HUPF | Dodecanoic acid | pos | C12H24O2 | 2.4618 | 1.264 | 0.02902 | up |
| F_HUPF | 1-[5-(Thiophen-2-ylmethoxy)-1H-indol-3-yl]propan-2-amine | neg | C16H18N2OS | 2.4471 | 0.8245 | 0.008216 | down |
| F_HUPF | N-Decanoyl-DL-Homoserine Lactone | pos | C14H25NO3 | 2.445 | 1.2687 | 0.01175 | up |
| F_HUPF | Agar | pos | C14H24O9 | 2.4425 | 1.2881 | 0.01877 | up |
| F_HUPF | N-[[2-(4-Amino-1,2,5-oxadiazol-3-yl)-1-ethylimidazo[5,4-d]pyridin-7-yl]methyl]piperidin-4-amine | neg | C16H22N8O | 2.4421 | 1.3562 | 0.008439 | up |
| F_HUPF | Gabapentin enacarbil | neg | C16H27NO6 | 2.4403 | 1.279 | 0.01961 | up |
| F_HUPF | 2-Amino-N-[1-[[2-[[1-(2-hydroxyethylamino)-1-oxo-3-phenylpropan-2-yl]-methylamino]-2-oxoethyl]amino]-1-oxopropan-2-yl]-3-(4-hydroxyphenyl)propanamide | pos | C26H35N5O6 | 2.4306 | 1.4573 | 0.04564 | up |
| F_HUPF | Threonylphenylalanine | neg | C13H18N2O4 | 2.4255 | 1.401 | 0.0306 | up |
| F_HUPF | (E)-4,5-Dihydro-6-(2-(4-pyridinyl)ethenyl)-3(2H)-pyridazinone | pos | C11H11N3O | 2.4043 | 1.276 | 0.02611 | up |
| F_HUPF | Diphenylamine | pos | C12H11N | 2.4022 | 0.7411 | 0.01118 | down |
| F_HUPF | (2E,4E,7E)-Nona-2,4,7-trienedioylcarnitine | neg | C16H23NO6 | 2.3995 | 1.3006 | 0.001301 | up |
| F_HUPF | Clomipramine | neg | C19H23ClN2 | 2.3995 | 1.2462 | 0.03662 | up |
| F_HUPF | Cibaric acid | pos | C18H28O5 | 2.3953 | 1.2948 | 0.02847 | up |
| F_HUPF | 2-Phenylaminoadenosine | pos | C16H18N6O4 | 2.3945 | 1.2211 | 0.0007331 | up |
| F_HUPF | Tafluprost acid | pos | C22H28F2O5 | 2.3811 | 1.2149 | 0.01052 | up |
| F_HUPF | Acetamide, 2-((2-hydroxyethyl)thio)-N-(3-(3-(1-piperidinylmethyl)phenoxy)propyl)- | neg | C19H30N2O3S | 2.3761 | 1.4063 | 0.02994 | up |
| F_HUPF | Glycylprolylhydroxyproline | pos | C12H19N3O5 | 2.3542 | 1.28 | 0.02891 | up |
| F_HUPF | Hetacillin | neg | C19H23N3O4S | 2.3412 | 0.5673 | 0.04914 | down |
| F_HUPF | 6-Ketoestriol | pos | C18H22O4 | 2.337 | 1.2493 | 0.006906 | up |
| F_HUPF | 1-O-p-Coumaroyl-(b-D-glucose 6-O-sulfate) | neg | C15H18O11S | 2.3334 | 1.329 | 0.01861 | up |
| F_HUPF | 5,10-Pentadecadien-1-ol | pos | C15H28O | 2.3198 | 1.2622 | 0.01734 | up |
| F_HUPF | Fludrocortisone | pos | C21H29FO5 | 2.3018 | 1.2164 | 0.0194 | up |
| F_HUPF | Taxiphyllin | neg | C14H17NO7 | 2.2962 | 1.311 | 0.004492 | up |
| F_HUPF | 4-O-alpha-D-Galactopyranosylcalystegine B2 | pos | C13H23NO9 | 2.2933 | 1.2216 | 0.01418 | up |
| F_HUPF | Entacapone | neg | C14H15N3O5 | 2.2684 | 1.2888 | 0.008158 | up |
| F_HUPF | Lamivudine | pos | C8H11N3O3S | 2.2474 | 1.2418 | 0.01402 | up |
| F_HUPF | Coniferin | neg | C16H22O8 | 2.243 | 1.2131 | 0.002589 | up |
| F_HUPF | 6-Keto-prostaglandin F1a | pos | C20H34O6 | 2.2387 | 1.2017 | 0.009213 | up |
| F_HUPF | Flavonol 3-O-D-glucoside | neg | C21H20O8 | 2.2227 | 1.2046 | 0.006932 | up |
| F_HUPF | N'-Hydroxyneosaxitoxin | pos | C10H17N7O6 | 2.215 | 1.288 | 0.0351 | up |
| F_HUPF | Genipin 1-gentiobioside | neg | C23H34O15 | 2.2114 | 1.2645 | 0.04079 | up |
| F_HUPF | 15-KETE | pos | C20H30O3 | 2.2068 | 1.2067 | 0.0294 | up |
| F_HUPF | Chlorambucil | pos | C14H19Cl2NO2 | 2.1994 | 1.221 | 0.007144 | up |
| F_HUPF | (x)-2-Heptanol glucoside | pos | C13H26O6 | 2.1838 | 1.276 | 0.04802 | up |
| F_HUPF | 5-Chloro-2-hydroxy-7-methoxy-2H-1,4-benzoxazin-3(4H)-one beta-D-glucopyranoside | neg | C15H18ClNO9 | 2.1808 | 0.8078 | 0.007764 | down |
| F_HUPF | Gibberellin A43 | neg | C20H26O8 | 2.1669 | 1.3624 | 0.03542 | up |
| F_HUPF | Cis-Acetylacrylate | neg | C5H6O3 | 2.1606 | 1.2219 | 0.003761 | up |
| F_HUPF | Serylarginine | neg | C9H19N5O4 | 2.1508 | 1.2748 | 0.01717 | up |
| F_HUPF | (8S,9Z,11E,14Z)-8-Hydroxyicosa-9,11,14-trienoylcarnitine | pos | C27H47NO5 | 2.1488 | 1.2141 | 0.02539 | up |
| F_HUPF | [(3-Hexyl-4-methyl-2-oxo-2H-chromen-7-yl)oxy]acetic acid | neg | C18H22O5 | 2.1463 | 1.2153 | 0.003895 | up |
| F_HUPF | Histidylleucine | pos | C12H20N4O3 | 2.1439 | 0.8128 | 0.03384 | down |
| F_HUPF | 4-Fluoro-L-phenylalanine | pos | C9H10FNO2 | 2.1421 | 1.2585 | 0.02901 | up |
| F_HUPF | Glutarate semialdehyde | pos | C5H8O3 | 2.1359 | 1.2275 | 0.04295 | up |
| F_HUPF | Beta-Leucine | pos | C6H13NO2 | 2.1348 | 1.2025 | 0.02995 | up |
| F_HUPF | Yakuchinone-A | pos | C20H24O3 | 2.1297 | 1.2032 | 0.01727 | up |
| F_HUPF | N-Benzyl-D-glucamine | neg | C13H21NO5 | 2.1219 | 1.2741 | 0.01161 | up |
| F_HUPF | Eicosapentaenoic acid | pos | C20H30O2 | 2.1139 | 1.2014 | 0.02059 | up |
| F_HUPF | 3-[4-(sulfooxy)phenyl]propanoic acid | neg | C9H10O6S | 2.1139 | 1.3491 | 0.04406 | up |
| F_HUPF | Leucyl-Glutamine | pos | C11H21N3O4 | 2.0933 | 1.2221 | 0.0212 | up |
| F_HUPF | N-Myristoyl Glutamine | neg | C19H36N2O4 | 2.0724 | 1.2595 | 0.01715 | up |
| F_HUPF | Oxotremorine | neg | C12H18N2O | 2.0718 | 1.2077 | 0.008887 | up |
| F_HUPF | (2R)-2-Acetamido-6-hydroxy-2-(sulfanylmethyl)hex-3-enoic acid | pos | C9H15NO4S | 2.0673 | 1.2333 | 0.04872 | up |
| F_HUPF | 4-(3,4-Dihydroxyphenyl)-2,3-dihydro-2,3-dihydroxy-1H-phenalen-1-one | neg | C19H14O5 | 2.0159 | 1.2451 | 0.0147 | up |
| F_HUPF | N-Methyl-L-histidine | neg | C7H11N3O2 | 2.0147 | 1.2023 | 0.007091 | up |
| F_HUPF | Asparaginylhydroxyproline | neg | C9H15N3O5 | 1.9744 | 1.2913 | 0.0358 | up |
| F_HUPF | Dopamine | neg | C8H11NO2 | 1.9728 | 1.2261 | 0.004975 | up |
| F_HUPF | Furanone A | neg | C4H4O2 | 1.9526 | 1.2879 | 0.04884 | up |
| F_HUPF | 5-Ethyl-1,2,3,4,5,6-hexahydro-7H-cyclopenta[b]pyridin-7-one | neg | C10H15NO | 1.9249 | 1.3357 | 0.04028 | up |
| F_HUPF | Cyclo(L-Phe-L-Pro) | neg | C14H16N2O2 | 1.9247 | 1.2901 | 0.03632 | up |
| F_HUPF | 3-Methyl-1,2-cyclohexanedione | neg | C7H10O2 | 1.904 | 1.2505 | 0.02231 | up |
| F_HUPF | Lersivirine | neg | C17H18N4O2 | 1.8606 | 1.2575 | 0.03749 | up |
| F_HUPF | Turicine | neg | C7H13NO3 | 1.8133 | 1.2661 | 0.02951 | up |
| F_HUPF | Benzeneacetic acid, 4-(2-(diethylamino)-2-oxoethoxy)-3-ethoxy-, propyl ester | neg | C19H29NO5 | 1.7938 | 1.2435 | 0.03739 | up |
| F_UPD | Beta-L-Fucose | neg | C6H12O5 | 6.284 | 1.9027 | 3.99E-06 | up |
| F_UPD | 1-Hexacosanol | pos | C26H54O | 6.2147 | 0.2957 | 0.004479 | down |
| F_UPD | N-Ethylnorcotinine | neg | C11H14N2O | 5.7866 | 1.8321 | 4.65E-07 | up |
| F_UPD | Momelotinib | neg | C23H22N6O2 | 5.1144 | 2.7567 | 0.01042 | up |
| F_UPD | 1,3,7-Trimethyluric Acid | pos | C8H10N4O3 | 4.934 | 1.5101 | 7.10E-05 | up |
| F_UPD | 8,9-Epoxyeicosatrienoic acid | pos | C20H32O3 | 4.5013 | 0.4599 | 0.01949 | down |
| F_UPD | 3beta-hydroxy-4beta-methyl-5alpha-cholest-7-ene-4alpha-carboxylate | pos | C29H47O3- | 4.4661 | 1.6106 | 0.02409 | up |
| F_UPD | 3'-Deoxyderhamnosylmaysin | neg | C21H18O9 | 4.4229 | 0.679 | 0.003081 | down |
| F_UPD | Fumitremorgin B | pos | C27H33N3O5 | 4.3242 | 1.4718 | 0.04249 | up |
| F_UPD | 1-[(4-Amino-3-methylphenyl)methyl]-5-(2,2-diphenylacetyl)-6,7-dihydro-4H-imidazo[4,5-c]pyridine-6-carboxylic acid | pos | C29H28N4O3 | 4.2864 | 1.7588 | 0.04384 | up |
| F_UPD | FA 15 (antioxidant) | pos | C31H49NO2 | 4.229 | 1.4825 | 0.04206 | up |
| F_UPD | Leucopelargonidin | pos | C15H14O6 | 4.1877 | 2.0666 | 0.02647 | up |
| F_UPD | D-Xylulose | neg | C5H10O5 | 4.1201 | 0.638 | 0.00713 | down |
| F_UPD | M-Coumaric acid | neg | C9H8O3 | 3.8961 | 1.543 | 0.02137 | up |
| F_UPD | Cadabicine | pos | C25H29N3O4 | 3.7022 | 0.8034 | 0.007014 | down |
| F_UPD | Thiodi-glycolic acid | neg | C4H6O6S | 3.5761 | 0.7498 | 0.02154 | down |
| F_UPD | 3-(4-Carboxybenzylidene)-6-hydroxycamphor | pos | C18H20O4 | 3.534 | 1.772 | 0.01291 | up |
| F_UPD | Dukunolide E | neg | C26H28O9 | 3.3338 | 1.2737 | 0.02233 | up |
| F_UPD | N-(N-L-gamma-Glutamyl-S-nitroso-L-cysteinyl)glycine | neg | C10H16N4O7S | 3.324 | 1.4156 | 0.04821 | up |
| F_UPD | Ethyl (S)-3-hydroxybutyrate glucoside | pos | C12H22O8 | 3.0119 | 1.2609 | 0.02492 | up |
| F_UPD | Pantethine | neg | C22H42N4O8S2 | 2.996 | 0.8201 | 0.01045 | down |
| F_UPD | 5-p-Coumaroylquinic acid | neg | C16H18O8 | 2.9947 | 1.2542 | 0.01083 | up |
| F_UPD | S-Adenosylmethionine | pos | C15H23N6O5S+ | 2.9167 | 1.2146 | 0.02503 | up |
| F_UPD | Netilmicin | pos | C21H41N5O7 | 2.8012 | 1.2592 | 0.0375 | up |
| F_UPD | Benzamide, 4-chloro-N-(2-(3-oxo-4-morpholinyl)ethyl)- | neg | C13H15ClN2O3 | 2.7659 | 1.5339 | 0.0345 | up |
| F_UPD | Cefroxadine | pos | C16H19N3O5S | 2.7353 | 1.2496 | 0.04409 | up |
| F_UPD | Merodesmosine | pos | C18H34N4O6 | 2.7027 | 0.78 | 0.03429 | down |
| F_UPD | 4-Hydroxybenzeneacetonitrile | neg | C8H7NO | 2.7015 | 0.8331 | 0.03142 | down |
| F_UPD | Prostaglandin G2 | neg | C20H32O6 | 2.327 | 0.8071 | 0.0483 | down |
| M_HUPF | Phendimetrazine | neg | C12H17NO | 5.7066 | 2118.3547 | 8.34E-06 | up |
| M_HUPF | 1-Hexacosanol | pos | C26H54O | 5.2671 | 5.0168 | 0.001919 | up |
| M_HUPF | Hexahydro-6,7-dihydroxy-5-(hydroxymethyl)-3-(2-hydroxyphenyl)-2H-pyrano[2,3-d]oxazol-2-one | pos | C13H15NO7 | 4.8412 | 2.1811 | 0.001134 | up |
| M_HUPF | Phosphonol | pos | C6H17N2O3PS | 4.4081 | 2.0376 | 1.98E-05 | up |
| M_HUPF | 3-Carboxy-4-methyl-5-propyl-2-furanpropionic acid | pos | C12H16O5 | 4.3732 | 1.8335 | 0.001543 | up |
| M_HUPF | 4-Methylumbelliferone sulfate | neg | C10H8O6S | 4.3398 | 2.8706 | 7.43E-08 | up |
| M_HUPF | 1-(4-Aminophenyl)-7,8-dimethoxy-3,5-dihydro-2,3-benzodiazepin-4-one | neg | C17H17N3O3 | 4.2507 | 3.9117 | 0.002322 | up |
| M_HUPF | 2,6-Dimethoxy-1,4-benzoquinone | pos | C8H8O4 | 4.2448 | 2.4909 | 0.003015 | up |
| M_HUPF | Glycerol 1-(5-hydroxydodecanoate) | pos | C15H30O5 | 4.0591 | 3.8081 | 0.00887 | up |
| M_HUPF | (3s)-3-(Benzyloxy)-L-Aspartic Acid | pos | C11H13NO5 | 4.0037 | 1.7748 | 0.004351 | up |
| M_HUPF | Gamma-glutamyl-L-putrescine | pos | C9H19N3O3 | 3.9054 | 2.0775 | 0.009671 | up |
| M_HUPF | Gamma-Glutamylcysteinylserine | pos | C11H19N3O7S | 3.8981 | 2.0405 | 0.01757 | up |
| M_HUPF | [(2S,3S,4S,5S,6R)-4,5-Dihydroxy-2,6-dimethyloxan-3-yl] hydrogen sulfate | neg | C7H14O7S | 3.8317 | 0.32 | 7.90E-05 | down |
| M_HUPF | 5-Sulfosalicylic acid | neg | C7H6O6S | 3.7596 | 1.8733 | 3.92E-06 | up |
| M_HUPF | 4-Methylthiobenzamide-S-oxide | neg | C8H9NOS | 3.7261 | 2.0249 | 0.0006019 | up |
| M_HUPF | 15-Keto-prostaglandin E2 | pos | C20H30O5 | 3.7174 | 4.3289 | 0.04648 | up |
| M_HUPF | N-Methyltyramine | pos | C9H13NO | 3.7033 | 4.5777 | 0.009688 | up |
| M_HUPF | Gibberellin A5 | neg | C19H22O5 | 3.6805 | 2.2805 | 0.0009994 | up |
| M_HUPF | 4-N-Methyllyaloside | pos | C28H33N2O9+ | 3.6054 | 2.6976 | 0.04045 | up |
| M_HUPF | 3-Isopropylmalic acid | pos | C7H12O5 | 3.5915 | 0.5476 | 0.01074 | down |
| M_HUPF | Ethyl 2-furanyl diketone | pos | C8H8O3 | 3.5587 | 1.4351 | 2.54E-05 | up |
| M_HUPF | 4-amino-4-deoxychorismate | pos | C10H11NO5 | 3.5432 | 1.5293 | 0.007114 | up |
| M_HUPF | 3,5,6-Trihydroxy-5-(hydroxymethyl)-2-methoxy-2-cyclohexen-1-one | pos | C8H12O6 | 3.5054 | 2.0457 | 0.004903 | up |
| M_HUPF | 5-Acetyl-3,4-dihydro-2H-pyrrole | neg | C6H9NO | 3.5012 | 1.9843 | 0.0004439 | up |
| M_HUPF | Clofibryl glucuronide | neg | C16H19ClO9 | 3.4904 | 1.986 | 0.001101 | up |
| M_HUPF | Maleylacetoacetic acid | pos | C8H8O6 | 3.4747 | 1.6133 | 0.00215 | up |
| M_HUPF | Cephalexin | pos | C16H17N3O4S | 3.4738 | 1.8958 | 0.01203 | up |
| M_HUPF | Methysticin | pos | C15H14O5 | 3.4613 | 1.5931 | 0.02019 | up |
| M_HUPF | 24(28)-Dehydromakisterone | pos | C28H44O7 | 3.4317 | 2.443 | 0.04236 | up |
| M_HUPF | Epinephrine | pos | C9H13NO3 | 3.4282 | 1.4817 | 0.002348 | up |
| M_HUPF | (S)-[8]-Gingerol | neg | C19H30O4 | 3.3953 | 2.1418 | 0.012 | up |
| M_HUPF | Chenodeoxycholylalanine | neg | C27H45NO5 | 3.3702 | 0.5573 | 0.003636 | down |
| M_HUPF | N-Acetyl-L-Histidine | pos | C8H11N3O3 | 3.3159 | 1.9666 | 0.02574 | up |
| M_HUPF | Phenmetrazine | neg | C11H15NO | 3.2952 | 1.6389 | 0.00251 | up |
| M_HUPF | L-DOPA 3'-glucoside | neg | C15H21NO9 | 3.2932 | 5.2014 | 0.04037 | up |
| M_HUPF | Aloesol 7-glucoside | neg | C19H24O9 | 3.264 | 2.1626 | 0.00925 | up |
| M_HUPF | 6-Acetylmorphine | pos | C19H21NO4 | 3.2574 | 2.397 | 0.04018 | up |
| M_HUPF | Ptelatoside A | neg | C19H26O10 | 3.2487 | 2.186 | 0.01904 | up |
| M_HUPF | Laninamivir | pos | C13H22N4O7 | 3.2105 | 1.5508 | 0.02027 | up |
| M_HUPF | Benzamide, 4-chloro-N-(2-(3-oxo-4-morpholinyl)ethyl)- | neg | C13H15ClN2O3 | 3.1978 | 2.1408 | 0.002664 | up |
| M_HUPF | Reichstein's substance E | pos | C21H32O5 | 3.1947 | 1.7069 | 0.01331 | up |
| M_HUPF | 6,7-Dimethyl-8-(1-D-ribityl)lumazine | pos | C13H18N4O6 | 3.1851 | 1.6245 | 0.02098 | up |
| M_HUPF | 2'-Deoxymugineic acid | neg | C12H20N2O7 | 3.1275 | 2.7222 | 0.04091 | up |
| M_HUPF | Lys-Ile-OH | pos | C17H25N3O6 | 3.1265 | 1.3955 | 0.002754 | up |
| M_HUPF | Glycylprolylhydroxyproline | pos | C12H19N3O5 | 3.1152 | 1.8774 | 0.03349 | up |
| M_HUPF | 3-O-Methyl-a-methyldopa | neg | C10H13NO4 | 3.1086 | 1.6674 | 0.000399 | up |
| M_HUPF | Avenic acid A | pos | C12H22N2O8 | 3.0849 | 1.6212 | 0.01452 | up |
| M_HUPF | DG(PGD2/8:0/0:0) | neg | C31H52O8 | 3.0774 | 0.6836 | 6.01E-05 | down |
| M_HUPF | Pyridoxal 5'-phosphate | pos | C8H10NO6P | 3.0686 | 1.4934 | 0.01335 | up |
| M_HUPF | Hyaluronan biosynthesis, precursor 1 | neg | C14H21NO11 | 3.045 | 1.745 | 0.005702 | up |
| M_HUPF | 2-(1,2,3,4-Tetrahydroxybutyl)thiazolidine-4-carboxylic acid | pos | C8H15NO6S | 3.0287 | 1.5202 | 0.02362 | up |
| M_HUPF | Methylisopelletierine | pos | C9H17NO | 2.986 | 0.6683 | 0.04129 | down |
| M_HUPF | 10-Piperazinylpropylphenothiazine | neg | C19H23N3S | 2.976 | 1.84 | 0.005671 | up |
| M_HUPF | 2-Benzylidene-1-heptanol | pos | C14H20O | 2.9496 | 0.7775 | 1.24E-05 | down |
| M_HUPF | 1,5-Dimethyl citrate | pos | C8H12O7 | 2.9463 | 1.402 | 8.71E-05 | up |
| M_HUPF | Deoxycholylisoleucine | pos | C30H51NO5 | 2.9405 | 0.7534 | 0.01667 | down |
| M_HUPF | (S)-(+)-1-(p-Hydroxy-trans-cinnamoyl)-glycerol | neg | C12H14O5 | 2.9296 | 1.3984 | 0.004685 | up |
| M_HUPF | 7-(Trifluoromethoxy)indolin-2-one | pos | C9H6F3NO2 | 2.9239 | 0.7077 | 0.006788 | down |
| M_HUPF | 2-Fluoro-2',3'-dideoxyadenosine | neg | C10H12FN5O2 | 2.9225 | 1.4632 | 0.008145 | up |
| M_HUPF | Triterpenoid | pos | C30H48O7S | 2.917 | 0.548 | 0.005092 | down |
| M_HUPF | Sesamol | neg | C7H6O3 | 2.8714 | 1.3274 | 3.04E-06 | up |
| M_HUPF | 2-Phenylaminoadenosine | pos | C16H18N6O4 | 2.8579 | 1.3247 | 0.0009855 | up |
| M_HUPF | Blumealactone C | neg | C17H24O6 | 2.841 | 0.7679 | 7.24E-07 | down |
| M_HUPF | N-Acetyl-1,6-diaminohexane | pos | C8H18N2O | 2.8406 | 0.7487 | 0.00166 | down |
| M_HUPF | 2-Aminoethyl hydrogen sulfate | neg | C2H7NO4S | 2.8286 | 1.681 | 0.007506 | up |
| M_HUPF | 18-Hydroxycorticosterone | pos | C21H30O5 | 2.8284 | 1.3639 | 0.00684 | up |
| M_HUPF | Gibberellin A86 | neg | C19H24O8 | 2.825 | 1.4179 | 0.01153 | up |
| M_HUPF | Alpha-Ionene | pos | C13H18 | 2.8058 | 0.7289 | 0.002772 | down |
| M_HUPF | Deoxycholyltyrosine | pos | C33H49NO6 | 2.7931 | 0.8024 | 0.0008647 | down |
| M_HUPF | 12-Oxo-20-trihydroxy-leukotriene B4 | neg | C20H30O7 | 2.784 | 1.6793 | 0.03774 | up |
| M_HUPF | 9S-hydroxy-11,15-dioxo-5Z,13E-prostadienoic acid | neg | C20H30O5 | 2.7764 | 1.3755 | 0.001935 | up |
| M_HUPF | Indacrinone | neg | C18H14Cl2O4 | 2.7727 | 1.5315 | 0.009399 | up |
| M_HUPF | KOBUSONE | pos | C14H22O2 | 2.7625 | 0.7963 | 0.00438 | down |
| M_HUPF | Cnidilide | pos | C12H18O2 | 2.7624 | 0.8227 | 8.64E-06 | down |
| M_HUPF | N-Methylserotonin | pos | C11H14N2O | 2.7623 | 1.4132 | 0.002463 | up |
| M_HUPF | 3-Sulfopropyl methacrylate | neg | C7H12O5S | 2.751 | 1.7285 | 0.005626 | up |
| M_HUPF | Alginic acid | neg | C12H16O12P2 | 2.7273 | 1.5592 | 0.01932 | up |
| M_HUPF | Histidylglutamine | neg | C11H17N5O4 | 2.7215 | 1.7539 | 0.02994 | up |
| M_HUPF | Tryptophyl-Arginine | pos | C17H24N6O3 | 2.7188 | 1.4713 | 0.04976 | up |
| M_HUPF | Strigol | pos | C19H22O6 | 2.7064 | 1.2842 | 0.001316 | up |
| M_HUPF | Tryptophyl-Glutamine | neg | C16H20N4O4 | 2.6853 | 1.5049 | 0.01608 | up |
| M_HUPF | Cysteinyl-Arginine | pos | C9H19N5O3S | 2.6774 | 1.4628 | 0.04154 | up |
| M_HUPF | Benzeneacetic acid, 4-(2-(diethylamino)-2-oxoethoxy)-3-ethoxy-, propyl ester | neg | C19H29NO5 | 2.6768 | 1.5053 | 0.003897 | up |
| M_HUPF | 5-Acetyl-2,3-dihydro-1H-pyrrolizine | neg | C9H11NO | 2.6718 | 1.6806 | 0.01192 | up |
| M_HUPF | Melibiitol | pos | C12H24O11 | 2.6622 | 1.3567 | 0.02197 | up |
| M_HUPF | Dehydrocurdione | pos | C15H22O2 | 2.6593 | 0.826 | 0.00309 | down |
| M_HUPF | Coutaric acid | neg | C18H27N3O4 | 2.6587 | 1.4382 | 0.005859 | up |
| M_HUPF | N-Benzoylaspartic acid | neg | C11H11NO5 | 2.6481 | 1.6414 | 0.04784 | up |
| M_HUPF | N-Methylisoleucine | pos | C7H15NO2 | 2.6278 | 0.8173 | 0.0001163 | down |
| M_HUPF | 4-Tert-butyl-2-[(tert-butylamino)methyl]-6-(4-chlorophenyl)phenol | pos | C21H28ClNO | 2.61 | 0.7546 | 0.02403 | down |
| M_HUPF | Pyridin-4-ylmethyldiazene | pos | C6H7N3 | 2.6086 | 1.3267 | 0.01425 | up |
| M_HUPF | Oxacillin | neg | C19H19N3O5S | 2.6081 | 0.7138 | 0.005223 | down |
| M_HUPF | Chenodeoxycholyltyrosine | neg | C33H49NO6 | 2.6036 | 0.7663 | 0.004051 | down |
| M_HUPF | Cellobioside | neg | C14H26O10 | 2.5989 | 1.6751 | 0.03832 | up |
| M_HUPF | Bendiocarb | neg | C11H13NO4 | 2.5921 | 1.4223 | 0.01698 | up |
| M_HUPF | Norsanguinarine | neg | C19H11NO4 | 2.5854 | 0.7155 | 0.008184 | down |
| M_HUPF | Riddelliine | neg | C18H23NO6 | 2.5848 | 0.76 | 0.005594 | down |
| M_HUPF | Oxolinic acid | neg | C13H11NO5 | 2.5816 | 1.5142 | 0.01291 | up |
| M_HUPF | N-Succinyl-L,L-2,6-diaminopimelate | neg | C11H18N2O7 | 2.5781 | 1.6529 | 0.03379 | up |
| M_HUPF | S-Formylglutathione | neg | C11H17N3O7S | 2.5734 | 0.7362 | 0.00145 | down |
| M_HUPF | Epothilone B | pos | C27H41NO6S | 2.5664 | 1.3187 | 0.03792 | up |
| M_HUPF | Fenoxaprop | neg | C16H12ClNO5 | 2.5659 | 0.7034 | 0.01504 | down |
| M_HUPF | Tetraphyllin B | pos | C12H17NO7 | 2.562 | 1.3458 | 0.01295 | up |
| M_HUPF | Chlorambucil | pos | C14H19Cl2NO2 | 2.5619 | 1.3335 | 0.01758 | up |
| M_HUPF | Niazirinin | pos | C16H19NO6 | 2.5377 | 1.3221 | 0.02326 | up |
| M_HUPF | 3-(5-Acetamido-2-hydroxyphenyl)sulfanyl-2-oxopropanoic acid | pos | C11H11NO5S | 2.5326 | 0.7122 | 0.0182 | down |
| M_HUPF | Gentamicin C | neg | C19H39N5O7 | 2.5325 | 1.4227 | 0.01616 | up |
| M_HUPF | 3,4-Methyleneadipic acid | neg | C8H10O4 | 2.5224 | 1.2614 | 3.53E-05 | up |
| M_HUPF | Biliverdin | neg | C33H34N4O6 | 2.5204 | 0.4468 | 0.04145 | down |
| M_HUPF | 2-Methylpropyl glucosinolate | neg | C11H21NO9S2 | 2.5138 | 0.73 | 0.008607 | down |
| M_HUPF | 2-Methylhippuric Acid | neg | C10H11NO3 | 2.513 | 1.3903 | 0.01782 | up |
| M_HUPF | Ethyl benzoate | pos | C9H10O2 | 2.4714 | 1.2473 | 0.01896 | up |
| M_HUPF | Oxazepam | pos | C15H11ClN2O2 | 2.4677 | 0.8181 | 0.007734 | down |
| M_HUPF | 1-Tert-Butyl 4-ethyl 3-oxopiperidine-1,4-dicarboxylate | neg | C13H21NO5 | 2.459 | 1.3723 | 0.007533 | up |
| M_HUPF | 4-Hydroxy-alprenolol | neg | C15H23NO3 | 2.4526 | 1.7318 | 0.02159 | up |
| M_HUPF | N-methylethanolamine phosphate | neg | C3H9NO4P- | 2.4458 | 1.418 | 0.009537 | up |
| M_HUPF | Alpha-AMINO-3-HYDROXY-5-METHYL-4-ISOXAZOLEPROPIONIC ACID | pos | C7H10N2O4 | 2.4395 | 0.7983 | 0.00417 | down |
| M_HUPF | 8,9-Epoxyeicosatrienoic acid | pos | C20H32O3 | 2.4386 | 1.2645 | 0.009537 | up |
| M_HUPF | (S,E)-Zearalenone | neg | C18H22O5 | 2.4325 | 1.5402 | 0.03806 | up |
| M_HUPF | Isopentenyladenine-9-N-glucoside | neg | C16H23N5O5 | 2.4309 | 0.7915 | 5.87E-05 | down |
| M_HUPF | L-Aspoxicillin trihydrate | neg | C21H27N5O7S | 2.4259 | 1.4081 | 0.02228 | up |
| M_HUPF | Benzamide | neg | C7H7NO | 2.4174 | 1.2835 | 0.004521 | up |
| M_HUPF | Proline betaine | pos | C7H13NO2 | 2.4107 | 1.2269 | 0.03962 | up |
| M_HUPF | 11-Hydroxy-9-tridecenoic acid | pos | C13H24O3 | 2.4042 | 1.289 | 0.03185 | up |
| M_HUPF | Turicine | neg | C7H13NO3 | 2.4032 | 1.3828 | 0.01251 | up |
| M_HUPF | Darexaban | neg | C27H30N4O4 | 2.4022 | 0.687 | 0.04079 | down |
| M_HUPF | Dynorphin B (10-13) | pos | C20H39N5O6 | 2.4001 | 1.2746 | 0.01719 | up |
| M_HUPF | Indole-3-acetamide | neg | C10H10N2O | 2.3978 | 1.3146 | 0.0243 | up |
| M_HUPF | Eperezolid | neg | C18H23FN4O5 | 2.3963 | 1.2393 | 4.99E-06 | up |
| M_HUPF | S-Allylcysteine | pos | C6H11NO2S | 2.3916 | 1.2686 | 0.01372 | up |
| M_HUPF | 2-Propionylpyrrole | neg | C7H9NO | 2.3915 | 1.4931 | 0.02975 | up |
| M_HUPF | 2-amino-5-(amino-dimethylamino-methylidene)amino-pentanoic acid | pos | C8H18N4O2 | 2.391 | 1.2592 | 0.008943 | up |
| M_HUPF | LysoPC(15:0/0:0) | pos | C23H48NO7P | 2.3713 | 0.8009 | 0.03523 | down |
| M_HUPF | Dihydrocorticosterone | pos | C21H32O4 | 2.3691 | 1.2314 | 0.004067 | up |
| M_HUPF | Dehydromonocrotaline | pos | C16H21NO6 | 2.3623 | 1.2226 | 0.02184 | up |
| M_HUPF | Butabarbital | neg | C10H16N2O3 | 2.3605 | 1.4662 | 0.02406 | up |
| M_HUPF | Androstenedione | pos | C19H26O2 | 2.3598 | 1.301 | 0.0007484 | up |
| M_HUPF | 13-(3-Methyl-5-pentylfuran-2-yl)tridecanoylcarnitine | neg | C30H53NO5 | 2.3569 | 0.739 | 0.03831 | down |
| M_HUPF | N(6)-6(R,S)-lipoyl-L-lysine | neg | C14H26N2O3S2 | 2.3564 | 1.3736 | 0.03773 | up |
| M_HUPF | Prostaglandin D3 | neg | C20H30O5 | 2.3552 | 1.2666 | 0.004485 | up |
| M_HUPF | 6-Ketoprostaglandin E1 | neg | C20H32O6 | 2.3527 | 1.2941 | 0.00353 | up |
| M_HUPF | Glycyl-Histidine | neg | C8H12N4O3 | 2.3522 | 0.7558 | 0.001735 | down |
| M_HUPF | Chenodeoxycholylleucine | neg | C30H51NO5 | 2.3495 | 0.8145 | 0.003538 | down |
| M_HUPF | 2,1,3-Benzoxadiazole-4-sulfonic acid, 7-fluoro- | neg | C6H3FN2O4S | 2.349 | 1.4309 | 0.03836 | up |
| M_HUPF | 6-Acetyl-1,2,3,4-tetrahydropyridine | neg | C7H11NO | 2.34 | 1.5687 | 0.04913 | up |
| M_HUPF | (4Z,9a)-9-(3-Methyl-2-butenoyloxy)-4,10(14)-oplopadien-3-one | pos | C20H28O3 | 2.3364 | 1.303 | 0.04373 | up |
| M_HUPF | Ile Ile Ala | pos | C15H29N3O4 | 2.3334 | 0.8241 | 0.003193 | down |
| M_HUPF | Ovalicin | pos | C16H24O5 | 2.3235 | 0.8333 | 3.23E-05 | down |
| M_HUPF | 12-oxo-20-dihydroxy-leukotriene B4 | pos | C20H30O6 | 2.3147 | 1.2289 | 0.03074 | up |
| M_HUPF | Leu-Gly-Gly | neg | C10H19N3O4 | 2.3132 | 0.7425 | 0.004336 | down |
| M_HUPF | 4-Hydroxy-L-phenylglycine | neg | C8H9NO3 | 2.292 | 1.2263 | 0.001758 | up |
| M_HUPF | Arginine ornithine | pos | C11H24N6O3 | 2.2767 | 0.8288 | 0.003947 | down |
| M_HUPF | 4-Amino-1-[(2R,5R)-5-(aminomethyl)-3,4-dihydroxyoxolan-2-yl]pyrimidin-2-one | pos | C9H14N4O4 | 2.2723 | 0.7881 | 0.02148 | down |
| M_HUPF | [(3-Hexyl-4-methyl-2-oxo-2H-chromen-7-yl)oxy]acetic acid | neg | C18H22O5 | 2.2664 | 1.2918 | 0.02056 | up |
| M_HUPF | 15-Hydroxy-11alpha,9alpha-(epoxymethano)prosta-5,13-dienoic acid | pos | C21H34O4 | 2.2581 | 0.8314 | 0.005779 | down |
| M_HUPF | 11-Dehydrocorticosterone | pos | C21H28O4 | 2.2501 | 1.25 | 0.02868 | up |
| M_HUPF | L-Proline, 1-(1-L-leucyl-L-prolyl)- | pos | C16H27N3O4 | 2.2499 | 1.3828 | 0.04767 | up |
| M_HUPF | Sisomicin sulfate | pos | C19H37N5O7 | 2.2172 | 0.8088 | 0.02719 | down |
| M_HUPF | (3E)-Glutaconylcarnitin | pos | C12H19NO6 | 2.2092 | 1.3075 | 0.03889 | up |
| M_HUPF | 16-B1-phytoprostane | pos | C18H28O4 | 2.2086 | 1.225 | 0.03241 | up |
| M_HUPF | Genipin 1-gentiobioside | neg | C23H34O15 | 2.1895 | 1.3786 | 0.04042 | up |
| M_HUPF | Threonylphenylalanine | neg | C13H18N2O4 | 2.1875 | 1.4292 | 0.02892 | up |
| M_HUPF | M-Cresol | neg | C7H8O | 2.1811 | 1.2729 | 0.008583 | up |
| M_HUPF | Prostaglandin D2 | neg | C20H32O5 | 2.1744 | 1.2754 | 0.02396 | up |
| M_HUPF | Besifloxacin | pos | C19H21ClFN3O3 | 2.1612 | 0.7881 | 0.04069 | down |
| M_HUPF | Aminosalicylic Acid | neg | C7H7NO3 | 2.1324 | 1.2592 | 0.01637 | up |
| M_HUPF | Tyrosyl-Proline | pos | C14H18N2O4 | 2.1292 | 1.2543 | 0.03232 | up |
| M_HUPF | N-(3-Hydroxypropyl)phthalimide | pos | C11H11NO3 | 2.1231 | 0.8167 | 0.01658 | down |
| M_HUPF | 1-Cyclohexyl-2-aziridinemethanol | pos | C9H17NO | 2.1138 | 0.795 | 0.03152 | down |
| M_HUPF | Prunasin | pos | C14H17NO6 | 2.1073 | 1.2137 | 0.009933 | up |
| M_HUPF | S-nirvanol | neg | C11H12N2O2 | 2.0992 | 1.217 | 0.007071 | up |
| M_HUPF | Thermophillin | pos | C8H8O4 | 2.0965 | 1.269 | 0.03092 | up |
| M_HUPF | DIPROTIN B | neg | C16H29N3O4 | 2.0921 | 0.7761 | 0.02924 | down |
| M_HUPF | PGD2 ethanolamide | neg | C22H37NO5 | 2.0827 | 0.8148 | 0.03653 | down |
| M_HUPF | S-(2,2-Dichloro-1-hydroxy)ethyl glutathione | neg | C12H19Cl2N3O7S | 2.0801 | 0.7885 | 0.04178 | down |
| M_HUPF | 5H-Indazole-5,5-dicarboxylicacid, 1,2,3,4,6,7-hexahydro-3-oxo- | neg | C9H10N2O5 | 2.0777 | 0.7789 | 0.0234 | down |
| M_HUPF | 4-Hydroxy Duloxetine | pos | C18H19NO2S | 2.0774 | 1.2153 | 0.02866 | up |
| M_HUPF | 1,6-anhydro-N-acetyl-beta-muramate | pos | C11H16NO7- | 2.0749 | 1.2102 | 0.02138 | up |
| M_HUPF | Coniferin | neg | C16H22O8 | 2.0735 | 1.2623 | 0.03046 | up |
| M_HUPF | Terazosin | neg | C19H25N5O4 | 2.0498 | 1.2695 | 0.0426 | up |
| M_HUPF | Abametapir | neg | C12H12N2 | 2.048 | 1.3071 | 0.02496 | up |
| M_HUPF | Oxymetholone | pos | C21H32O3 | 2.0443 | 1.208 | 0.008918 | up |
| M_HUPF | Ethyl butylacetylaminopropionate | pos | C11H21NO3 | 2.0279 | 1.2382 | 0.03359 | up |
| M_HUPF | Islatravir | neg | C12H12FN5O3 | 2.0237 | 0.827 | 0.01366 | down |
| M_HUPF | Gibberellin A43 | neg | C20H26O8 | 2.0033 | 1.3131 | 0.04473 | up |
| M_HUPF | Entacapone | neg | C14H15N3O5 | 1.9994 | 1.262 | 0.02773 | up |
| M_HUPF | Ile-Val-OH | neg | C16H22N2O6 | 1.9846 | 1.251 | 0.03041 | up |
| M_HUPF | Asparaginylhydroxyproline | neg | C9H15N3O5 | 1.984 | 1.3686 | 0.03945 | up |
| M_HUPF | 7-Hydroxymitragynine | neg | C23H30N2O5 | 1.9819 | 1.2001 | 0.01577 | up |
| M_HUPF | Cycloalliin | neg | C6H11NO3S | 1.9749 | 0.8205 | 0.02345 | down |
| M_HUPF | N-[[2-(4-Amino-1,2,5-oxadiazol-3-yl)-1-ethylimidazo[5,4-d]pyridin-7-yl]methyl]piperidin-4-amine | neg | C16H22N8O | 1.9633 | 1.3279 | 0.04856 | up |
| M_HUPF | 3-[(3-(2-Carboxyethyl)-4-methylpyrrol-2-YL)methylene]-2-indolinone | neg | C17H16N2O3 | 1.9531 | 1.2378 | 0.01551 | up |
| M_HUPF | Chrysaloin | neg | C21H22O8 | 1.9232 | 0.8014 | 0.04862 | down |
| M_HUPF | 3-O-beta-D-Galactopyranosyl-L-arabinose | neg | C11H20O10 | 1.9229 | 0.8179 | 0.03279 | down |
| M_HUPF | (2E,4E,7E)-Nona-2,4,7-trienedioylcarnitine | neg | C16H23NO6 | 1.8948 | 1.2778 | 0.03776 | up |
| M_HUPF | 5-Chloro-2-hydroxy-7-methoxy-2H-1,4-benzoxazin-3(4H)-one beta-D-glucopyranoside | neg | C15H18ClNO9 | 1.883 | 0.8276 | 0.02278 | down |
| M_HUPF | Taps | neg | C7H17NO6S | 1.8801 | 1.2058 | 0.03935 | up |
| M_HUPF | Vulgaxanthin I | neg | C14H17N3O7 | 1.8651 | 0.8283 | 0.02304 | down |
| M_HUPF | Cumi-101 | pos | C19H27N5O3 | 1.848 | 1.2113 | 0.04716 | up |
| M_HUPF | 2-Methylbenzyl alcohol acetate | neg | C10H12O2 | 1.7888 | 0.8227 | 0.04261 | down |
| M_HUPF | Hexanoylglycine | neg | C8H15NO3 | 1.7857 | 0.7721 | 0.04164 | down |
| M_HUPF | Taxiphyllin | neg | C14H17NO7 | 1.758 | 1.2025 | 0.047 | up |
| M_HUPF | Chandalone | neg | C25H24O5 | 1.758 | 1.2038 | 0.04557 | up |
| M_LUPF | Momelotinib | neg | C23H22N6O2 | 5.6295 | 4.518 | 4.57E-13 | up |
| M_LUPF | 3-[5-(Dimethylcarbamoyl)pyrrolidin-3-yl]sulfanyl-6-(1-hydroxyethyl)-4-methyl-7-oxo-1-azabicyclo[3.2.0]hept-2-ene-2-carboxylic acid | pos | C17H25N3O5S | 5.1179 | 2.9981 | 0.0001212 | up |
| M_LUPF | Paliperidone | pos | C23H27FN4O3 | 5.0451 | 2.6813 | 0.004399 | up |
| M_LUPF | Samidorphan | pos | C21H26N2O4 | 4.9713 | 2.3964 | 0.0003739 | up |
| M_LUPF | Phendimetrazine | neg | C12H17NO | 4.9181 | 1474.5828 | 0.00502 | up |
| M_LUPF | ((4-(4-Amidinophenoxy)butanoyl)aspartyl)valine | pos | C20H28N4O7 | 4.6643 | 2.8066 | 0.0009922 | up |
| M_LUPF | (R)-1-O-[b-D-Glucopyranosyl-(1->6)-b-D-glucopyranoside]-1,3-octanediol | pos | C20H38O12 | 4.3489 | 1.6384 | 2.99E-08 | up |
| M_LUPF | 4-Methylumbelliferone sulfate | neg | C10H8O6S | 4.3031 | 2.5091 | 8.07E-05 | up |
| M_LUPF | 2-Propenamide, 2-cyano-3-(4-hydroxy-3,5-bis(1-methylethyl)phenyl)- | pos | C16H20N2O2 | 4.3029 | 1.7185 | 2.76E-11 | up |
| M_LUPF | 5-Oxo-prolyl-glycyl-arginine-4-nitroanilide | neg | C19H26N8O6 | 4.2802 | 1.8183 | 4.12E-05 | up |
| M_LUPF | 6-Deoxy-4-O-(3,6-di-O-methyl-beta-D-glucopyranosyl)-2,3-di-O-methyl-alpha-L-mannopyranose | pos | C16H30O10 | 4.1904 | 1.6629 | 0.001694 | up |
| M_LUPF | 25-Acetyl-6,7-didehydrofevicordin F 3-[glucosyl-(1->6)-glucoside] | neg | C43H62O18 | 4.1541 | 1.5503 | 6.70E-11 | up |
| M_LUPF | Guanidine, N-((2S,3S,4R)-6-amino-2-(dimethoxymethyl)-3,4-dihydro-3-hydroxy-2-methyl-2H-1-benzopyran-4-yl)-N'-cyano-N''-(phenylmethyl)- | pos | C22H27N5O4 | 4.1264 | 1.5343 | 9.44E-10 | up |
| M_LUPF | Netilmicin | pos | C21H41N5O7 | 4.1161 | 1.6844 | 5.96E-08 | up |
| M_LUPF | Ethyl (S)-3-hydroxybutyrate glucoside | pos | C12H22O8 | 4.0385 | 1.5642 | 1.09E-05 | up |
| M_LUPF | Dihydro-beta-erythroidine | pos | C16H21NO3 | 4.0273 | 1.5955 | 2.99E-09 | up |
| M_LUPF | 6alpha-hydroxy-castasterone | pos | C28H50O5 | 4.0107 | 0.4481 | 0.03951 | down |
| M_LUPF | Gly-Pro-Arg-Pro-Lys | pos | C24H43N9O6 | 4.0018 | 1.561 | 4.61E-10 | up |
| M_LUPF | Cefroxadine | pos | C16H19N3O5S | 3.9776 | 1.5851 | 2.73E-10 | up |
| M_LUPF | Armillatin | pos | C38H58O6 | 3.9591 | 1.4195 | 2.88E-06 | up |
| M_LUPF | (2S)-2-Cyclopentyl-2-[4-[(2,4-dimethylpyrido[2,3-b]indol-9-yl)methyl]phenyl]-N-[(1S)-2-hydroxy-1-phenylethyl]acetamide | pos | C35H37N3O2 | 3.8952 | 1.5308 | 4.86E-10 | up |
| M_LUPF | 1-Hexacosanol | pos | C26H54O | 3.8647 | 3.7157 | 0.04793 | up |
| M_LUPF | Hexahydro-6,7-dihydroxy-5-(hydroxymethyl)-3-(2-hydroxyphenyl)-2H-pyrano[2,3-d]oxazol-2-one | pos | C13H15NO7 | 3.7022 | 1.7276 | 0.005152 | up |
| M_LUPF | Baohuoside I | neg | C27H30O10 | 3.6767 | 1.6707 | 0.005407 | up |
| M_LUPF | Phenobarbital | pos | C12H12N2O3 | 3.6694 | 1.4729 | 4.08E-06 | up |
| M_LUPF | Spirorenone | neg | C24H28O3 | 3.641 | 1.4279 | 2.61E-07 | up |
| M_LUPF | 4-Methylthiobenzamide-S-oxide | neg | C8H9NOS | 3.6367 | 1.7526 | 0.0005724 | up |
| M_LUPF | 1-(4-ethoxyphenyl)-3-[2-(1-ethylindol-3-yl)-2-pyridin-3-ylethyl]urea | pos | C26H28N4O2 | 3.6241 | 1.3394 | 6.48E-07 | up |
| M_LUPF | Delavirdine | pos | C22H28N6O3S | 3.6173 | 1.4538 | 1.43E-09 | up |
| M_LUPF | 5-Sulfosalicylic acid | neg | C7H6O6S | 3.5945 | 1.6202 | 6.85E-06 | up |
| M_LUPF | 6-Acetylmorphine | pos | C19H21NO4 | 3.591 | 2.2472 | 0.00314 | up |
| M_LUPF | Glutaminylisoleucine | neg | C11H21N3O4 | 3.5862 | 2.6629 | 0.01376 | up |
| M_LUPF | 1H-Pyrrole-3-carboxamide, 5-((5-fluoro-1,2-dihydro-2-oxo-3H-indol-3-ylidene)methyl)-N-((2S)-2-hydroxy-3-(4-morpholinyl)propyl)-2,4-dimethyl- | pos | C23H27FN4O4 | 3.5714 | 1.3439 | 2.32E-06 | up |
| M_LUPF | Sparfloxacin | pos | C19H22F2N4O3 | 3.5514 | 1.3366 | 2.44E-06 | up |
| M_LUPF | ADP Ribose | neg | C15H23N5O14P2 | 3.5082 | 1.7188 | 0.02162 | up |
| M_LUPF | (2R,4S)-4-Carbamimidamido-3-acetamido-2-((1R,2R)-2-hydroxy-1-methoxy-3-(octanoyloxy)propyl)-3,4-dihydro-2H-pyran-6-carboxylic acid | pos | C21H36N4O8 | 3.4938 | 1.3188 | 5.92E-07 | up |
| M_LUPF | Isopropyl apiosylglucoside | pos | C14H26O10 | 3.4877 | 1.3088 | 1.07E-07 | up |
| M_LUPF | Pregnenolone sulfate | neg | C21H32O5S | 3.4536 | 1.4025 | 1.38E-07 | up |
| M_LUPF | Loganate | pos | C16H23O10- | 3.4267 | 1.4722 | 2.62E-10 | up |
| M_LUPF | Furohyperforin | pos | C35H52O5 | 3.4143 | 1.2579 | 1.72E-10 | up |
| M_LUPF | Amikacin | pos | C22H43N5O13 | 3.4026 | 1.3588 | 2.92E-09 | up |
| M_LUPF | Isomaltoside | neg | C14H26O10 | 3.3905 | 1.373 | 1.09E-07 | up |
| M_LUPF | Gamma-Glutamylcysteinylserine | pos | C11H19N3O7S | 3.3525 | 1.7139 | 0.009988 | up |
| M_LUPF | N-Succinyl-L,L-2,6-diaminopimelate | neg | C11H18N2O7 | 3.3343 | 1.795 | 0.01144 | up |
| M_LUPF | Pegvaliase | neg | C15H30N2O5 | 3.3211 | 0.5953 | 0.01073 | down |
| M_LUPF | 4-Tert-butyl-2-[(tert-butylamino)methyl]-6-(4-chlorophenyl)phenol | pos | C21H28ClNO | 3.3125 | 0.6519 | 0.00873 | down |
| M_LUPF | Phosphonol | pos | C6H17N2O3PS | 3.3069 | 1.5843 | 0.0002425 | up |
| M_LUPF | 2'-Deoxymugineic acid | neg | C12H20N2O7 | 3.3009 | 2.2821 | 0.00803 | up |
| M_LUPF | Forodesine | pos | C11H14N4O4 | 3.2818 | 1.3377 | 3.28E-06 | up |
| M_LUPF | 4-N-Methyllyaloside | pos | C28H33N2O9+ | 3.2566 | 2.376 | 0.04225 | up |
| M_LUPF | 12alpha-Hydroxy-13,18-dehydroparain | pos | C21H28O6 | 3.2483 | 0.6986 | 5.89E-05 | down |
| M_LUPF | S-Adenosylmethionine | pos | C15H23N6O5S+ | 3.2274 | 1.3498 | 0.0007028 | up |
| M_LUPF | DG(PGD2/8:0/0:0) | neg | C31H52O8 | 3.2134 | 0.721 | 0.0004661 | down |
| M_LUPF | Estriol-16-Glucuronide | pos | C24H32O9 | 3.2016 | 0.7275 | 0.008149 | down |
| M_LUPF | 3-Carboxy-4-methyl-5-propyl-2-furanpropionic acid | pos | C12H16O5 | 3.1718 | 1.4706 | 0.00907 | up |
| M_LUPF | 2-[1-[(2S)-2-[[4-[(E)-N'-Hydroxycarbamimidoyl]benzoyl]amino]propanoyl]piperidin-4-yl]oxyacetic acid | neg | C18H24N4O6 | 3.1322 | 1.2679 | 2.13E-05 | up |
| M_LUPF | Limonoate a-ring-lactone | pos | C26H32O9 | 3.1097 | 0.7334 | 0.0003484 | down |
| M_LUPF | Cyclo(Arg-Gly-Asp-D-Phe-Val) | neg | C26H38N8O7 | 3.0934 | 1.2645 | 4.72E-07 | up |
| M_LUPF | Benzoylaconine | neg | C32H45NO10 | 3.0893 | 1.2467 | 5.29E-08 | up |
| M_LUPF | Bisdemethoxycurcumin | neg | C19H16O4 | 3.0861 | 0.6951 | 0.01036 | down |
| M_LUPF | (3s)-3-(Benzyloxy)-L-Aspartic Acid | pos | C11H13NO5 | 3.0503 | 1.4543 | 0.007127 | up |
| M_LUPF | Coformycin | neg | C11H16N4O5 | 3.0259 | 1.3522 | 0.0001109 | up |
| M_LUPF | Glycerol 1-(5-hydroxydodecanoate) | pos | C15H30O5 | 3.0256 | 2.7792 | 0.04206 | up |
| M_LUPF | N-acetyl-S-(3-oxo-3-carboxy-n-propyl)cysteine | pos | C9H13NO6S | 3.0014 | 0.6929 | 0.00642 | down |
| M_LUPF | 7,8-dihydroneopterin 3'-phosphate | neg | C9H12N5O7P-2 | 2.9718 | 0.5846 | 0.04197 | down |
| M_LUPF | Clofibryl glucuronide | neg | C16H19ClO9 | 2.9599 | 1.6868 | 0.01627 | up |
| M_LUPF | 3-Sulfopropyl methacrylate | neg | C7H12O5S | 2.9542 | 1.6535 | 0.01429 | up |
| M_LUPF | N-Methylphenylalanyl-prolyl-arginine | pos | C21H32N6O4 | 2.9522 | 1.2716 | 7.31E-06 | up |
| M_LUPF | 6-Chloro-5-(4-(1-hydroxycyclobutyl)phenyl)-1H-indole-3-carboxylic acid | neg | C19H16ClNO3 | 2.9453 | 1.3294 | 0.02088 | up |
| M_LUPF | 5-Acetyl-3,4-dihydro-2H-pyrrole | neg | C6H9NO | 2.934 | 1.6347 | 0.008333 | up |
| M_LUPF | 2,6-Dimethoxy-1,4-benzoquinone | pos | C8H8O4 | 2.9325 | 1.8042 | 0.02384 | up |
| M_LUPF | Glycylprolylhydroxyproline | pos | C12H19N3O5 | 2.9259 | 1.7266 | 0.04772 | up |
| M_LUPF | Ethyl 2-furanyl diketone | pos | C8H8O3 | 2.8925 | 1.2916 | 0.0003615 | up |
| M_LUPF | DG(2:0/20:3(8Z,11Z,14Z)-2OH(5,6)/0:0) | pos | C25H42O7 | 2.8785 | 1.2911 | 0.001005 | up |
| M_LUPF | Gravacridonol | pos | C19H17NO4 | 2.8674 | 1.2925 | 0.0001059 | up |
| M_LUPF | Piromidic acid | neg | C14H16N4O3 | 2.8458 | 1.259 | 5.86E-06 | up |
| M_LUPF | Bakers yeast extract | pos | C19H14O2 | 2.8162 | 1.3141 | 2.97E-06 | up |
| M_LUPF | Prostaglandin PGE2 1-glyceryl ester | neg | C23H38O7 | 2.8103 | 1.3269 | 3.09E-05 | up |
| M_LUPF | Arginine ornithine | pos | C11H24N6O3 | 2.8096 | 0.7941 | 0.0001194 | down |
| M_LUPF | 3-Hydroxyocta-2,5-dienoylcarnitine | pos | C15H25NO5 | 2.7969 | 1.2284 | 2.87E-05 | up |
| M_LUPF | Gibberellin A5 | neg | C19H22O5 | 2.7846 | 1.7377 | 0.03145 | up |
| M_LUPF | N-(N-(3-Amino-3-carboxypropyl)-3-amino-3-carboxypropyl)azetidine-2-carboxylic acid | neg | C12H21N3O6 | 2.7574 | 0.7693 | 0.001516 | down |
| M_LUPF | Testosterone phenylpropionate | neg | C28H36O3 | 2.7568 | 0.7294 | 0.006525 | down |
| M_LUPF | Inhibitor 50 | neg | C18H17N3O8S | 2.729 | 0.7764 | 0.004347 | down |
| M_LUPF | Deoxyloganin | pos | C17H26O9 | 2.7275 | 1.2756 | 0.0004854 | up |
| M_LUPF | 4H-Thieno[3,2-b]pyrrole-5-carboxylic acid | pos | C7H5NO2S | 2.7256 | 1.2892 | 0.006138 | up |
| M_LUPF | RUCAPARIB | neg | C19H18FN3O | 2.6924 | 1.2452 | 0.0008448 | up |
| M_LUPF | 4-amino-4-deoxychorismate | pos | C10H11NO5 | 2.6903 | 1.2869 | 0.003996 | up |
| M_LUPF | Tryptophyl-Glutamine | neg | C16H20N4O4 | 2.6886 | 1.3739 | 0.0144 | up |
| M_LUPF | Antiarrhythmic peptide | neg | C19H30N6O8 | 2.6805 | 0.7797 | 0.005959 | down |
| M_LUPF | Phenmetrazine | neg | C11H15NO | 2.6721 | 1.3599 | 0.008214 | up |
| M_LUPF | Gamma-Aminobutyric acid glutamate | neg | C9H16N2O5 | 2.6649 | 1.2858 | 0.001198 | up |
| M_LUPF | Chrysaloin | neg | C21H22O8 | 2.6325 | 0.7569 | 0.01569 | down |
| M_LUPF | Sobetirome | pos | C20H24O4 | 2.5998 | 1.3771 | 0.01401 | up |
| M_LUPF | Seryltryptophan | neg | C14H17N3O4 | 2.5955 | 1.2293 | 1.63E-05 | up |
| M_LUPF | Tryptophyl-Glutamate | pos | C16H19N3O5 | 2.5861 | 1.2037 | 0.0003367 | up |
| M_LUPF | (Z)-Resveratrol 3-(4''-sulfoglucoside) | neg | C20H22O11S | 2.57 | 0.7562 | 0.006414 | down |
| M_LUPF | Dihydrodiethylstilbestrol | pos | C18H22O2 | 2.5451 | 0.8281 | 1.83E-05 | down |
| M_LUPF | 9S-hydroxy-11,15-dioxo-5Z,13E-prostadienoic acid | neg | C20H30O5 | 2.5337 | 1.2654 | 0.01218 | up |
| M_LUPF | Lamivudine | pos | C8H11N3O3S | 2.4912 | 1.2207 | 0.001455 | up |
| M_LUPF | 3-O-Methyl-a-methyldopa | neg | C10H13NO4 | 2.487 | 1.3574 | 0.002149 | up |
| M_LUPF | 10-Piperazinylpropylphenothiazine | neg | C19H23N3S | 2.4806 | 1.5024 | 0.01187 | up |
| M_LUPF | Dimethylbenzimidazole | pos | C9H10N2 | 2.4736 | 1.3754 | 0.04392 | up |
| M_LUPF | Laninamivir | pos | C13H22N4O7 | 2.4632 | 1.3102 | 0.01621 | up |
| M_LUPF | 5,8-Epoxy-5,8-dihydro-3-hydroxy-8'-apo-b,y-carotenal | neg | C30H40O3 | 2.4613 | 0.7381 | 0.01987 | down |
| M_LUPF | Methysticin | pos | C15H14O5 | 2.4588 | 1.3153 | 0.03221 | up |
| M_LUPF | 2-(Methylthio)ethyl glucosinolate | neg | C10H19NO9S3 | 2.4496 | 0.7848 | 0.022 | down |
| M_LUPF | Zatebradine | pos | C26H36N2O5 | 2.4409 | 0.8028 | 0.04707 | down |
| M_LUPF | N-formimidoyl-glutamic acid | neg | C6H10N2O4 | 2.4369 | 0.7135 | 0.02875 | down |
| M_LUPF | 7alpha-hydroxyestradiol | pos | C18H24O3 | 2.408 | 0.7401 | 0.02093 | down |
| M_LUPF | 4-(3,4-Dihydroxyphenyl)-2,3-dihydro-2,3-dihydroxy-1H-phenalen-1-one | neg | C19H14O5 | 2.4035 | 1.2057 | 4.78E-05 | up |
| M_LUPF | (-)-Morphine | pos | C17H19NO3 | 2.3829 | 0.7897 | 0.001923 | down |
| M_LUPF | Difluprednate | pos | C27H34F2O7 | 2.3665 | 0.7643 | 0.01301 | down |
| M_LUPF | Darexaban | neg | C27H30N4O4 | 2.3555 | 0.7553 | 0.04689 | down |
| M_LUPF | Pyridin-4-ylmethyldiazene | pos | C6H7N3 | 2.3532 | 1.281 | 0.02373 | up |
| M_LUPF | 1-[5-(Thiophen-2-ylmethoxy)-1H-indol-3-yl]propan-2-amine | neg | C16H18N2OS | 2.3511 | 0.7957 | 0.02165 | down |
| M_LUPF | N(6)-6(R,S)-lipoyl-L-lysine | neg | C14H26N2O3S2 | 2.3475 | 1.2478 | 0.005696 | up |
| M_LUPF | Epinephrine | pos | C9H13NO3 | 2.3462 | 1.229 | 0.004711 | up |
| M_LUPF | Dukunolide E | neg | C26H28O9 | 2.3305 | 0.7793 | 0.04603 | down |
| M_LUPF | N-Acetyl-1,6-diaminohexane | pos | C8H18N2O | 2.3225 | 0.8281 | 0.004677 | down |
| M_LUPF | Gibberellin A86 | neg | C19H24O8 | 2.3064 | 1.2314 | 0.01228 | up |
| M_LUPF | 2-Aminoethyl hydrogen sulfate | neg | C2H7NO4S | 2.305 | 1.3615 | 0.007544 | up |
| M_LUPF | Tyr-Gly-Gly-Trp-Leu | pos | C30H38N6O7 | 2.3012 | 0.8185 | 0.03179 | down |
| M_LUPF | Cadabicine | pos | C25H29N3O4 | 2.2958 | 0.8322 | 0.04012 | down |
| M_LUPF | 4-(Glutamylamino) butanoate | neg | C9H16N2O5 | 2.2852 | 1.2351 | 0.01321 | up |
| M_LUPF | Aloe-Emodin | pos | C15H10O5 | 2.2751 | 0.8231 | 0.0154 | down |
| M_LUPF | Ginkgolide A | neg | C20H24O9 | 2.2715 | 0.8017 | 0.008107 | down |
| M_LUPF | Esmolol | pos | C16H25NO4 | 2.2543 | 1.2771 | 0.03696 | up |
| M_LUPF | Artemidinol | pos | C13H12O3 | 2.254 | 0.7925 | 0.008743 | down |
| M_LUPF | Xanthotoxol glucoside | neg | C17H16O9 | 2.2503 | 0.8074 | 0.01914 | down |
| M_LUPF | Mauritine A | pos | C32H41N5O5 | 2.2044 | 1.2379 | 0.01301 | up |
| M_LUPF | Oxolinic acid | neg | C13H11NO5 | 2.1972 | 1.3469 | 0.03697 | up |
| M_LUPF | Glutathione episulfonium ion | pos | C12H20N3O6S+ | 2.1923 | 0.8177 | 0.04536 | down |
| M_LUPF | Arginyl-glycyl-glutamyl-serine | pos | C16H29N7O8 | 2.1545 | 1.2709 | 0.04326 | up |
| M_LUPF | Indacrinone | neg | C18H14Cl2O4 | 2.1535 | 1.2744 | 0.01692 | up |
| M_LUPF | P-Methylhippuric acid | pos | C10H11NO3 | 2.1472 | 1.3437 | 0.0439 | up |
| M_LUPF | O-Desmethyltramadol glucuronide | pos | C21H31NO8 | 2.1367 | 1.2608 | 0.0175 | up |
| M_LUPF | 2-(4-Hydroxyphenylazo)benzoic acid | neg | C13H10N2O3 | 2.1143 | 0.8211 | 0.04749 | down |
| M_LUPF | 5-Acetyl-2,3-dihydro-1H-pyrrolizine | neg | C9H11NO | 2.1135 | 1.3265 | 0.007385 | up |
| M_LUPF | Cephalexin | pos | C16H17N3O4S | 2.051 | 1.3665 | 0.04593 | up |
| M_LUPF | 2-Naphthoic acid | pos | C11H8O2 | 2.046 | 0.6596 | 0.01121 | down |
| M_LUPF | Deoxycholic acid 3-glucuronide | pos | C30H48O10 | 2.0405 | 0.8328 | 0.04398 | down |
| M_LUPF | Bendiocarb | neg | C11H13NO4 | 2.0007 | 1.211 | 0.02318 | up |
| M_LUPF | 2-(1,2,3,4-Tetrahydroxybutyl)thiazolidine-4-carboxylic acid | pos | C8H15NO6S | 1.9667 | 1.2402 | 0.04552 | up |
| M_LUPF | Maleylacetoacetic acid | pos | C8H8O6 | 1.8528 | 1.2172 | 0.03587 | up |
| M_LUPF | 4-Hydroxy-alprenolol | neg | C15H23NO3 | 1.8137 | 1.2935 | 0.02785 | up |
| M_LUPF | Alginic acid | neg | C12H16O12P2 | 1.783 | 1.2246 | 0.04817 | up |
| M_LUPF | Plumbagin | neg | C11H8O3 | 1.5626 | 1.2487 | 0.03119 | up |
| M_LUPF | Beta-L-Fucose | neg | C6H12O5 | 6.2006 | 1.9672 | 4.66E-06 | up |
| M_LUPF | 15-Keto-prostaglandin E2 | pos | C20H30O5 | 5.782 | 4.58 | 0.008463 | up |
| M_LUPF | Sivifene | neg | C19H14N4O6 | 5.7814 | 0.5254 | 6.63E-08 | down |
| M_LUPF | N-Ethylnorcotinine | neg | C11H14N2O | 5.6464 | 1.9617 | 3.93E-06 | up |
| M_LUPF | (1R,3As,4S,6aS)-1,4-di(benzo[d][1,3]dioxol-5-yl)hexahydrofuro[3,4-c]furan | neg | C20H18O6 | 5.4873 | 0.5467 | 2.66E-07 | down |
| M_LUPF | Hetacillin | neg | C19H23N3O4S | 5.1488 | 0.4666 | 0.01283 | down |
| M_LUPF | 1,3,7-Trimethyluric Acid | pos | C8H10N4O3 | 4.873 | 1.5756 | 0.000238 | up |
| M_LUPF | S-(2,2-Dichloro-1-hydroxy)ethyl glutathione | neg | C12H19Cl2N3O7S | 4.864 | 0.6517 | 6.29E-10 | down |
| M_LUPF | 7,8-Dihydropteroic acid | pos | C14H14N6O3 | 4.824 | 0.6192 | 3.12E-09 | down |
| M_LUPF | 4-Deacetylneosolaniol | pos | C17H24O7 | 4.8124 | 1.8204 | 0.00348 | up |
| M_LUPF | N-Succinyl-L,L-2,6-diaminopimelate | neg | C11H18N2O7 | 4.6939 | 1.8865 | 0.01301 | up |
| M_LUPF | Formyl-5-hydroxykynurenamine | pos | C10H12N2O3 | 4.6585 | 0.4415 | 0.02839 | down |
| M_LUPF | (8R,9R,10R,13S,14S)-10,13-Dimethyl-2,3,4,7,8,9,11,12,14,15,16,17-dodecahydro-1H-cyclopenta[a]phenanthrene-3,7,17-triol | pos | C19H30O3 | 4.537 | 2.4317 | 0.01439 | up |
| M_LUPF | Mammeisin | pos | C25H26O5 | 4.4975 | 1.7892 | 0.003806 | up |
| M_LUPF | Biliverdin | neg | C33H34N4O6 | 4.4614 | 1.7675 | 0.02472 | up |
| M_LUPF | 1-Hydroxyanthraquinone | neg | C14H8O3 | 4.4527 | 1.5051 | 8.86E-05 | up |
| M_LUPF | Indoxyl Sulfate | neg | C8H7NO4S | 4.3877 | 0.5677 | 0.04208 | down |
| M_LUPF | 1-[(4-Amino-3-methylphenyl)methyl]-5-(2,2-diphenylacetyl)-6,7-dihydro-4H-imidazo[4,5-c]pyridine-6-carboxylic acid | pos | C29H28N4O3 | 4.3574 | 1.7211 | 0.02256 | up |
| M_LUPF | Ethyl 2-hydroxy-3-(3-indolyl)propanoate glucoside | pos | C19H25NO8 | 4.2498 | 1.7898 | 0.003613 | up |
| M_LUPF | 1,2-Cyclohexanediol | pos | C6H12O2 | 4.227 | 0.7146 | 0.0001193 | down |
| M_LUPF | Kanzonol J | neg | C26H30O5 | 3.9803 | 0.6855 | 0.0004058 | down |
| M_LUPF | Tyr-Gly-Gly-Phe-Gly-OH | pos | C24H29N5O7 | 3.8929 | 0.743 | 0.0004921 | down |
| M_LUPF | 5-p-Coumaroylquinic acid | neg | C16H18O8 | 3.8221 | 1.4307 | 0.004626 | up |
| M_LUPF | Tfllr-NH2 | pos | C31H53N9O6 | 3.8027 | 0.7594 | 0.02361 | down |
| M_LUPF | Benzamide, 4-chloro-N-(2-(3-oxo-4-morpholinyl)ethyl)- | neg | C13H15ClN2O3 | 3.7189 | 1.7886 | 0.007188 | up |
| M_LUPF | Glycocholenate sulfate | neg | C26H41NO9S | 3.6781 | 1.2857 | 0.006351 | up |
| M_LUPF | Actarit | pos | C10H11NO3 | 3.6317 | 0.7966 | 0.0002664 | down |
| M_LUPF | PA(14:1(9Z)/20:5(6E,8Z,11Z,14Z,17Z)-OH(5)) | pos | C37H61O9P | 3.5652 | 0.7831 | 0.005295 | down |
| M_LUPF | Arginyl-glycyl-glutamyl-serine | pos | C16H29N7O8 | 3.519 | 1.4884 | 0.03515 | up |
| M_LUPF | Dimethylbenzimidazole | pos | C9H10N2 | 3.406 | 1.4138 | 0.03583 | up |
| M_LUPF | Cichorioside F | pos | C21H28O10 | 3.3619 | 0.802 | 0.004765 | down |
| M_LUPF | Gentamicin C | neg | C19H39N5O7 | 3.3274 | 1.3521 | 0.04219 | up |
| M_LUPF | 2-Naphthoic acid | pos | C11H8O2 | 3.224 | 0.5648 | 0.000551 | down |
| M_LUPF | Amastatin | pos | C21H38N4O8 | 3.2188 | 0.8196 | 0.003195 | down |
| M_LUPF | 2-Aminoethyl hydrogen sulfate | neg | C2H7NO4S | 3.1325 | 1.3298 | 7.26E-05 | up |
| M_LUPF | Myosmine | pos | C9H10N2 | 3.1147 | 1.2217 | 0.01356 | up |
| M_LUPF | N-(N-(3-Amino-3-carboxypropyl)-3-amino-3-carboxypropyl)azetidine-2-carboxylic acid | neg | C12H21N3O6 | 3.1122 | 0.8122 | 0.01438 | down |
| M_LUPF | 2,3-Dihydroxy-9,10,11-trimethoxy-5,8,13,13a-tetrahydroxy-6H-dibenzo(a,g)chinolysin | neg | C20H23NO5 | 3.1013 | 1.3884 | 0.04565 | up |
| M_LUPF | Piperyline | pos | C16H17NO3 | 2.9695 | 0.8236 | 0.02192 | down |
| M_LUPF | 4-Methylthiobenzamide-S-oxide | neg | C8H9NOS | 2.3647 | 1.2253 | 0.006231 | up |
